# Abstracts of the International Research Society on Spinal Deformities (IRSSD) Meeting 2018: Utrecht, the Netherlands. 14-16 June 2018

**DOI:** 10.1186/s13013-018-0154-2

**Published:** 2018-06-11

## O1 Health care professionals recognize adolescent idiopathic scoliosis in earlier stage compared to untrained adults

### Charlotte de Groot, Johan L. Heemskerk, Mark C. Altena, Diederik H. R. Kempen

#### OLVG, JointResearch, Amsterdam, The Netherlands

**Introduction/ background**

In the Netherlands, routine mandatory school screening for scoliosis was abandoned in 2010 because the effectiveness and efficiency of the screening program was questioned. Due to canceling of the scoliosis screening program, detection of the scoliosis now depends on the ability of parents or acquaintances of the child to recognize their deformity. For trained health care workers, the sensitivity and specificity of the bending test varies from 46 to 84% and 78 to 93%, respectively. However, the ability to detect a scoliosis for untrained adults is unknown. Therefore, the aim of this study is to evaluate the ability to detect a scoliosis and determine the sensitivity of the bending test of a selected population in untrained adults.

**Objectives**

The primary aim of this study is to evaluate the ability of untrained adults to recognize a scoliosis in a selected population of scoliotic and non-scoliotic patients. We hypothesize that the external curve characteristics (Cobb angle or lumbar vs thoracic curves) influence the ability to recognize the scoliosis.

**Method**

The study is an observational cohort study. After obtaining informed consent, standardized photographs of scoliotic and non-scoliotic children were obtained in the upright and the Adam’s forward bending test position. These pictures were used for a questionnaire including 28 pictures with different curve sizes and locations of the curve. Subsequently, 100 untrained adults and 120 health care professionals between 25-55 years were approached to complete the questionnaire. Statistical analysis was done using an independent-samples t-test.

**Results**

Untrained adults are less likely to recognize scoliosis then health care professionals. (62% vs. 72% p<0,01) Sensitivity in curves from 10- 20 degrees is 49% and 64% (p<0,01) for untrained adults and professionals , respectively. When the curves sizes are bigger the sensitivity increases; 65% vs. 73% (p<0,01) for curves between 20-40 degrees and 75% vs. 83% (p<0,01)for curves above 40 degrees. See Fig. 1. Thoracic curves above 40 degrees are all recognized, sensitivity is 99% vs. 100% for adults and professionals, respectively. Specificity is almost equal for untrained adults and health care professionals; 63% vs. 65%. False positives ratio is 36,75% vs. 35,215%.

**Conclusion**

Based on our findings untrained adults are less likely to recognize a scoliosis than health care professionals. Professionals are better able to recognize curves with a Cobb angle below 40 degrees compared to untrained adults. Consequently, scoliotic deformities in children are more likely to be detected in an advanced stage in the absence of a screening program.

**Fig. 1 (abstract O1).**
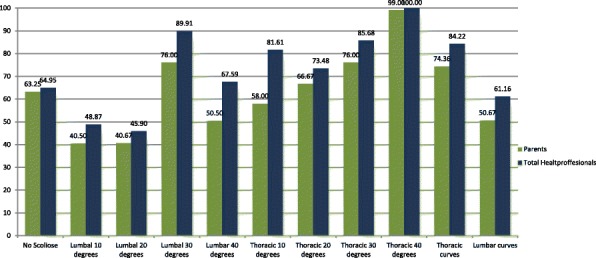
Sensitivity of the curves (%)

## O2 What is relative anterior spinal overgrowth of the adolescent idiopathic scoliotic spine?

### Rob C. Brink^1^, Tom P. C. Schlösser^1^, Marijn van Stralen^2^, Koen L. Vincken^3^, Moyo C. Kruyt^1^, Steve C. N. Hui^4^, Max A. Viergever^3^, Winnie C. W. Chu^4^, Jack C. Y. Cheng^5^, René M. Castelein^1^

#### ^1^Department of Orthopaedic Surgery, University Medical Center Utrecht, Utrecht, The Netherlands; ^2^Imaging Division, University Medical Center Utrecht, Utrecht, The Netherlands; ^3^Image Sciences Institute, University Medical Center Utrecht, Utrecht, The Netherlands; ^4^Department of Imaging & Interventional Radiology, Prince of Wales Hospital, The Chinese University of Hong Kong; ^5^Department of Orthopaedics and Traumatology, Prince of Wales Hospital, The Chinese University of Hong Kong, Shatin, Hong Kong

**Introduction**

One of the often reported observations in adolescent idiopathic scoliosis (AIS) is that the thoracic spine is longer anteriorly than posteriorly, most pronounced around the apex and the discs contribute more than the vertebral bodies. This so called relative anterior spinal overgrowth, or RASO, has been suggested as part of the etiologic mechanism of AIS. However, the role of the posterior spinal column and the lamina has so far not been elucidated, and, therefore, it remained unclear whether this anterior-posterior length discrepancy is the result of relative anterior lengthening or relative posterior shortening.

**Objective**

To define the discrepancy of the three-dimensional CT measured anterior-posterior length of the spinal column in idiopathic scoliosis versus controls.

**Methods**

IRB approval and patient consent was obtained. Consecutive series of high-resolution pre-operative CT scans of 80 moderate to severe AIS patients (Cobb angle: 46-109°) and scans of 30 non-scoliotic age-matched controls were analyzed. The height of the anterior and posterior vertebral bodies and discs, as well as the height of the laminae, interlaminar spaces, spinous processes and interspinous spaces of the thoracic curve, and corresponding levels in controls, were measured semi-automatically in the true mid sagittal plane of each individual vertebra.

**Results and discussions**

In AIS, the anterior height of the vertebral bodies and discs of the thoracic curve was 3.6±2.8% longer as compared to the posterior height, 2.0±6.1% longer than the length along the laminae and 8.7±7.1% longer than the length along the spinous processes and differed from controls (controls: -2.7±2.4%, -7.4±5.2% and +0.7±7.8%; p<0.001), with the non-osseous structures contributing significantly more to the length discrepancies. In absolute lengths, the anterior side of the disc of the thoracic curve was higher in AIS (5.4±0.8 mm) than controls (4.8±1.0 mm; p<0.001), whereas the interspinous space was smaller in AIS (12.3±1.4 mm versus 14.0±1.6 mm; p<0.001) and the absolute lengths of the osseous parts differed not significantly between AIS and controls.

**Conclusions and significance**

The true three-dimensional anterior-posterior length discrepancy of the scoliotic thoracic curves occur through both anterior column lengthening as well as posterior column shortening, with the facet joints as the fulcrum. In moderate to severe AIS, the vertebral bodies contribute partly to the anterior-posterior length discrepancy accompanied by more significant and possibly secondary increased anterior intervertebral discs height.

## O3 Association of circulating YKL-40 levels and *CHI3L1* gene variants with the risk of spinal deformity progression in adolescent idiopathic scoliosis

### Dina Nada^1,2^, Cédric Julien^1^, Pierre H. Rompré^3^, Marie-Yvonne Akoume^1^, Kristen F. Gorman^1,4^ Mark E. Samuels^5,6^, Emile Levy^5,7^, Dawei Li^8,9^, Alain Moreau^1,2,10,11^

#### ^1^Viscogliosi Laboratory in Molecular Genetics of Musculoskeletal Diseases, Sainte-Justine University Hospital, Research Center, Montreal, QC, Canada; ^2^Program of Biomedical Sciences, Faculty of Medicine, Université de Montréal, Montreal, QC, Canada; ^3^Faculty of Dentistry, Université de Montréal, Montreal, QC, Canada; ^4^Department of Biological Sciences, California State University, Chico, CA, USA; ^5^Sainte-Justine University Hospital Research Center, Montreal, QC, Canada; ^6^ Department of Medicine, Faculty of Medicine, Université de Montréal, Montreal, QC, Canada; ^7^Department of Nutrition, Faculty of Medicine, Université de Montréal, Montreal, QC, Canada; ^8^ Department of Microbiology and Molecular Genetics, University of Vermont, Burlington, VT, USA; ^9^ Department of Computer Science, Neuroscience, Behavior, and Health Initiative, University of Vermont, Burlington, VT, USA; ^10^Department of Biochemistry and Molecular Medicine, Faculty of Medicine, Université de Montréal, Montreal, QC, Canada; ^11^ Department of Stomatology, Faculty of Dentistry, Université de Montréal, Montreal, QC, Canada.

##### **Correspondence:** Alain Moreau (alain.moreau@recherche-ste-justine.qc.ca)

**Introduction**

The cellular and molecular mechanisms underlying spinal deformity progression in adolescent idiopathic scoliosis (AIS) remains poorly understood.

**Objectives**

To assess the contribution of circulating YKL-40 levels, a secreted glycoprotein encoded by chitinase 3-like 1 (CHI3L1) gene in AIS pathogenesis.

**Methods**

Plasma YKL-40 levels were determined by ELISA and SNPS were analyzed by multiplex polymerase chain reaction and genotyping. We genotyped 787 French-Canadian patients and 239 controls for 12 SNPs located in the CHI3L1 gene or its promoter. The occurrence of single polymorphisms, haplotypes and SNP-SNP interactions were statistically analyzed for association with disease risk, scoliosis severity phenotypes and endophenotypes as well as circulating YKL-40 levels. Functional effects of YKL-40 were tested on primary osteoblasts obtained from AIS patients by cellular dielectric spectroscopy assay.

**Results and discussion**

Plasma YKL-40 levels inversely correlates with the risk of spinal deformity progression in AIS. We observed a significant reduced risk of disease progression between eight SNPs (rs55700740, rs946259, rs880633, rs1538372, rs4950881, rs946261, rs946262 and rs10920576) associated with higher plasma YKL-40 levels. Circulating YKL-40 levels were also significantly increased in males classified in the first biological endophenotype (FG1) and associated with a non-severe scoliosis phenotype (< 40°). Treatment with YKL-40 rescued Gi-coupled receptor signaling dysfunction observed in AIS osteoblasts.

**Conclusion and significance**

Collectively, our findings reveal a novel role for YKL-40 in AIS pathogenesis and provide a first molecular mechanism by which this glycoprotein could interfere with spinal deformity progression.

## O4 POC5 and cilia anomalies in adolescent idiopathic scoliosis

### Amani Hassan, Stefan Parent, Charlotte Zaouter, Molidperee Sirinard, Soraya Barchi, Isabelle Villemure, Pierre Drapeau, Shunmoogum Patten, Florina Moldovan

#### Research Center of CHU Sainte-Justine, Faculty of Dentistry Université de Montréal, École Polytechnique and INRS–Institut Armand-Frappier, Montreal, Canada

**Introduction**

The etiology of adolescent idiopathic scoliosis (AIS) is largely unknown, but clinical observations revealed the role of hereditary and rapid growth in the development of this condition. More recently, several genes were suspected to cause or contribute to AIS. Our group identified gene variants of POC5 centriolar protein in a French and French-Canadian families with multiple members affected with AIS. We sought to expand on this study and to investigate for the role of POC5 gene and mutated protein.

Study design: The potential pathogenic effect of mutated POC5 was investigated in vitro (cell culture) and in vivo in a zebrafish animal model.

**Methods**

To investigate the role of POC5 in AIS, we investigated subcellular localization of POC5 with respect to cilia in cells overexpressing wt or POC5 variants (C1286T, A429V) and in human osteoblasts from scoliotic patients carrying these POC5 variants and normal control cells (in vitro study) . We also created a loss-of-function model in zebrafish (in vivo study). The role of POC5 was investigated by: 1) mass spectroscopy analysis and co-immunoprecipitation to identify differences in binding partners between the wild-type (wt POC5 and mut POC5 protein; 2) immunolocalization of POC5 wt and mut proteins at the cellular level; 3) histology and immunohistochemistry performed on tissues from wt (control) and scoliotic (poc5 mut) zebrafish.

**Results**

Our work identified several interacting partners with POC5, and documented functional connections with respect to cilia and centrosome dysfunction. A number of ciliary proteins were identified to be interacting with wt POC5 but not mut POC5. At the cellular level, localization and co-localisation of wt POC5 and mut POC5 protein with alpha acetylated tubulin (cilia marker), confirmed the consequence of the mutation on subcellular location with respect to cilium structure, length and staining intensity of cilia. In vivo, several defects in the retina were identified in mut poc5 zebrafish compared wt zebrafish. Finally, using different markers for retinal layers and acetylated tubulin, the defects were localized in ganglion cell layer and cones of the retina.

**Conclusion**

Our findings confirm the involvement of POC5 in scoliosis. A role of POC5 with respect to the primary cilia was attributed. These findings open new avenues for the understanding the primary causes of AIS at the molecular and physiological levels.

**Acknowledgments**

This study is supported by the Yves Cotrel Fondation-Institut de France, Université de Montréal and Scoliosis Research Society (SRS).

## O5 Diurnal variation of body height in children with idiopathic scoliosis

### Dariusz Czaprowski^1^, Marcin Tyrakowski^2^, Justyna Bloda^1^, Jakub Waś^1^, Anna Dembińska^3^, Tomasz Kotwicki^4^

#### ^1^Department of Physiotherapy, Józef Rusiecki University College, Olsztyn, Poland; ^2^Department of Orthopedics, Pediatric Orthopedics and Traumatology, Centre of Postgraduate Medical Education, Otwock, Poland; ^3^Department of Rehabilitation, Voivodeship Rehabilitation Hospital for Children, Olsztynek, Poland; ^4^Department of Spine Disorders and Pediatric Orthopedics, University of Medical Sciences, Poznan, Poland

**Introduction**

The measurement of body height (BH) is a part of clinical evaluation of children with idiopathic scoliosis (IS) as its progression is defined based on the observation of the growth spurt. The reliability of the height assessment may be limited by the measurement error. One of the sources of measurement error could be physiological diurnal variation of body height.

**Objective**

The aim of the study was to assess diurnal variation of body height in children with idiopathic scoliosis.

**Methods**

The study included 98 consecutive non-operatively treated patients with IS (82 females, 16 males, age 9-18 years, mean 14.4±1.8, Cobb 10°-52°, mean 21.2°±9.9). There were 22 patients with right thoracic (T), 35 with left thoracolumbar (TL), 20 with left lumbar (L) and 21 with double-curve thoracic and lumbar (DTL) scoliosis. BH was measured using a wall-mounted stadiometer in standardized standing and sitting positions. The measurements were performed 4 times a day: (1) just after getting-up between 7:00 and 8:00 - M1; (2) between 11:00 and 12:00 - M2; (3) between 15:00 and 16:00 - M3 and (4) between 19:00 and 20:00 - M4. All measurements were performed by the same observer. The subjects did not participate in any type of therapy or physical activity during the day of measurements.

**Results**

A significant BH decrease was observed for the entire group of patients: 164.7 ±10.7 cm (M1) vs. 164.2 ±10.7 cm (M2) vs. 164.1 ±10.6 cm (M3) vs. 164.0 ±10.7 cm (M4), p<0.001 and 85.9 ±5.6 cm (M1) vs. 85.5 ±5.7 cm (M2) vs. 85.3 ±5.6 cm (M3) vs. 85.2 ±5.5 cm (M4), p<0.001 for the standing and sitting position, respectively. The daily differences in BH ranged from +3.0 cm to -4.0 cm and from +2.5 cm to -1.5 cm, for standing and sitting, respectively. The highest BH decrease was observed between measurements performed just after getting-up (7:00-8:00) and measurements carried out in the evening (19:00-20:00). For standing, the mean height loss was 0.72 cm (±0.7), which equals 0.43% of initial standing height, while for sitting the mean height decrease was 0.69 cm (±0.7) which stated 0.79% of initial sitting height. The same pattern of BH changes was observed for T, TL, DTL (standing and sitting, p<0.001) and L (standing, p<0.001). Only BH measured in sitting position in patients with L the differences were insignificant (86.4 ±6.3 cm vs. 86.1 ±6.4 cm vs. 85.9 ±6.4 cm vs. 86.1 ±6.4 cm, p=0.09).

**Conclusions and significance**

The body height decreased in children and adolescents with IS during daytime. Due to diurnal body height variation the time of the day should be recorded when measuring patients with idiopathic scoliosis.

## O6 Cervical vertebral maturation stage in adolescent idiopathic scoliosis: Is it an alternative option in determining peak height velocity?

### Zezhang Zhu, Hongda Bao, Shibin Shu, Qi Gu, Yuancheng Zhang, Zhen Liu, Yong Qiu

#### Nanjing Drum Tower Hospital, Nanjing, 210008, China

**Introduction**

Commonly used clinical or radiographic methods including Risser sign, digital skeletal age (DSA) score, TW3 score, are inadequate or too complex for rapid application in a busy clinic setting. CVM stage (Fig. 1) is commonly used in Orthodontics but was less acknowledged in studies of spinal growth.

**Objective**

To investigate if the CVM stages could be used as an alternative option compared to Risser sign in determining PHV.

**Methods**

This is a retrospective longitudinal study. AIS patients were included with the following inclusion criteria: female, age between 9 to 16 y/o, have full spine images with clear visibility of cervical spine. AIS patients for growth validation also need more than 4 follow-ups with interval of at least 6 months Patients with clinical evidence of neurological abnormality or unclear Risser sign were excluded. Reliability test of CVM stage was performed at first with a fellow and a resident. The relationship between CVM and Risser sign was analyzed. The stature, arm span, spinal length, spinal height and pelvic height were measured at each follow-up, the growth velocity of each parameter were also calculated. The velocity at each CVM stage was compared.

**Results**

170 AIS patients were included for the first analysis (mean age 10.62 y/o). The distribution of CVM stage and Risser sign is shown in Fig. 1. The CVM stages were found to correlate strongly with Risser sign (r=0.85, p<0.01). In Risser 0 patients with open triradiate cartilage (TC), 39% was CVM 2 and 22% CVM 3; In Risser 0 patients with closed TC, 71% was CVM 3. 32 patients were included for growth validation study. The growth velocity of stature averaged 5.4 cm/yr in CVM 2 patients and 6.3cm/yr in CVM 3 patients, significantly larger than that in CVM 4 patients (3.3cm/yr, both p<0.001); similarly, the growth velocity of arm span and spinal length were also significantly higher in CVM 3 patients (6.2cm/yr and 4.0cm/yr). 68% patients showed CVM 3 at the time of PHV. The higher ratio of spinal length vs. pelvic height in CVM 3 also indicated the high growth velocity.

**Conclusion**

The new CVM stage could provide an alternative option for the assessment of skeletal maturity

of subjects with idiopathic scoliosis. The index needs to be subjected to further multicenter validation in different ethnic groups.

**Significance**

This study revealed a new parameter to assess the maturity status in adolescents.

**Fig. 1 (abstract O6).**
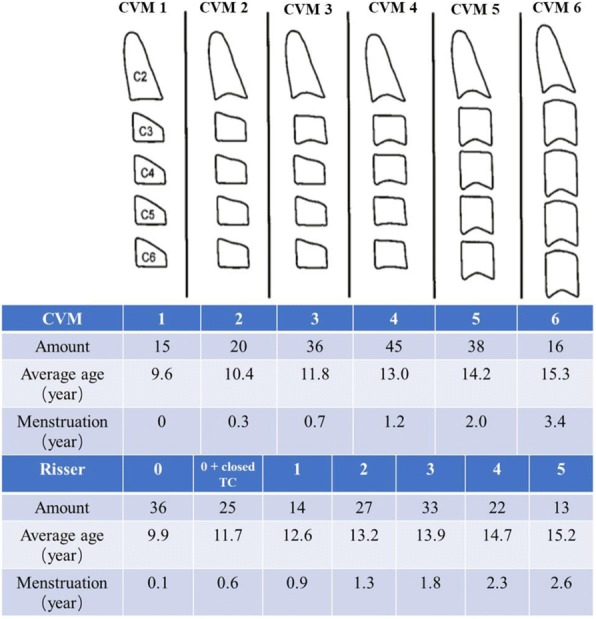
Schematic of CVM stage and the distribution of CVM/Risser Stage in the cohort

## O7 Serum bone metabolic markers in adolescent idiopathic scoliosis

### Yu-Jia Wang^1,2,3^, Jia-Jun Zhang^1,2,3^, Ka-Yee Cheuk^1,2,3^, Carol Ka-Lo Cheng^1,2,3^, Tsz-Ping Lam^1,2,3^, Yong Qiu^3,4^, Jack Chun-Yiu Cheng^1,2,3^, Wayne Yuk-Wai Lee^1,2,3^

#### ^1^Department of Orthopaedics and Traumatology, The Chinese University of Hong Kong, 999077, Hong Kong; ^2^SH Ho Scoliosis Research Laboratory, Faculty of Medicine, The Chinese University of Hong Kong, 999077, Hong Kong; ^3^Joint Scoliosis Research Center of the Chinese University of Hong Kong and Nanjing University, The Chinese University of Hong Kong, 999077, Hong Kong; ^4^Spine Surgery, Nanjing Drum Tower Hospital, Nanjing University, Nanjing, 210000, China

**Introduction**

Adolescent idiopathic scoliosis (AIS) is a three-dimensional spinal deformity mostly affecting girls during pubertal growth without clear etiology and pathogenesis. More than 30% of AIS patients have systemic low bone mass which is a prognostic factor for curve progression. Cumulative evidences have suggested systemic low bone mass as one of the potential pathomechanisms. Therefore, it is reasonable to speculate that there is a cross talk between curve progression and bone metabolism. Current understanding is mainly from the case-control study between control subjects and AIS patients as a whole. In this study, we hypothesize that AIS patients with mild and severe curve severities may also exhibit different serum bone metabolic profile.

**Objectives**

In this cross-sectional study, mild and severe cases were recruited to compare their serum bone metabolic markers levels and to study their correlation profile among anthropometric parameters and serum markers respectively.

**Methods**

33 mild and 50 severe AIS girls (age of 15.00 ± 1.04, 14.96 ± 1.81) were recruited. Patients in the cohort were ethnic Chinese and confirmed AIS excluding other known metabolic bone diseases. Anthropometric parameters were measured with standard stadiometry techniques. Six selective serum markers including bone lining cells activator parathyroid hormone, osteogenesis inhibitor sclerostin and dickkopf-1, osteoclastogenesis regulator osteoprotegerin and osteopontin, osteoblast marker osteocalcin were measured with Multiplex assay. Comparison study between groups and partial correlation analysis within each group were performed with SPSS.

**Results**

Mild and severe AIS subjects (Cobb angle of 24.64 ± 5.36°, 62.56 ± 12.95°) presented similar body weight, body mass index and Z-score of femoral neck BMD. Mild group had significantly lower level (p-value<0.05) in OPG and PTH compared with the severe group. Severe group presented significant negative correlation pattern between anthropometric parameters and serum markers (e.g. BMD and OCN, BMD and OPN) which were not observed in mild group. Consistently, significant correlations (p-value<0.01) between pairwise serum markers (e.g. DKK1 and OPG, SOST and OPG, PTH and OCN) were only found in the severe group.

**Conclusions and significance**

This is the first study demonstrating different serum bone metabolic markers and inconsistent correlations among anthropometric parameters and serum markers in AIS with stratification on curve severity. The different bone metabolic profiles between mild and severe groups suggest different bone metabolic status in AIS at later progression phase and point to the speculation that such difference may occur at early progression phase or even earlier. Further investigation with longitudinal dataset and larger sample size is warranted to delineate the dynamic change of bone metabolic profile in progressive and non-progressive groups, which will provide more insights into the pathomechanism.

## O8 Immediate tridimensional changes following anterior vertebral body growth modulation in adolescents with idiopathic scoliosis

### Olivier Turcot^1^, Marjolaine Roy-Beaudry^1^, Isabelle Turgeon^1^, Christian Bellefleur^2^, Stefan Parent^1**,**3^

#### ^1^CHU Sainte-Justine, Montréal, Québec, H3T 1C5, Canada; ^2^Polytechnique Montréal, Montréal, Québec, H3T 1J4, Canada; ^3^Université de Montréal, Montréal, Québec, H3T 1J4, Canada

**Introduction**

Anterior Vertebral Growth Modulation (AVBGM) aims to gradually correct scoliosis, using the patient’s growth, while preserving spine motion. One of the concerns is the risk of creating kyphosis.

**Objectives**

The first objective was to evaluate the 3D correction of scoliosis immediately after surgery to determine if 3D correction was achieved. The second objective was to characterize and analyse perioperative data. We hypothesize that 3D correction can be achieved without significant changes in the sagittal plane.

**Methods**

In this prospective developmental study we reviewed the clinical, perioperative and radiological prospectively collected data of the first 59 patients who received the AVBGM at our institution. The preoperative and 1^st^ erect visit (FE) data were analyzed. Computerized measurements were done on reconstructed 3D spine radiographs. Means, standard deviation and paired t test of specific parameters were calculated.

**Results and discussions**

All 59 patients were skeletally immature (mean age 11.8 yo). Mean operative time was 178 min with an EBL of 215 ml. Tethering was done on an average of 7.4 vertebral levels. Cobb angle was 49.7°±10.4° preoperatively and 29.6°±10,5° at the FE visit. In the sagittal plane, kyphosis was unchanged (27.7°±15.3° preoperatively and 28.2°±13.8° at the FE visit (p=0.743). The mean segmental derotated kyphosis (TrueKyphosis) of T5-T12 was 5.8°±11.3° preoperatively and 12.1°±11.7° at the FE visit (p<0.001). In the transverse plane, apical vertebral rotation of 13.0°±4.9° was corrected to 10.1°±6.6° postoperatively (p<0.001). The scoliosis research society-30 self-reported outcome questionnaire (SRS-30) showed an increased satisfaction with the management, even if pain was increased and function diminished.

**Conclusions and significance**

AVBT offers a significant correction in the coronal and transverse planes immediately post-op. Although the correction was achieved through an anterior compression approach, there was no impact on the kyphosis of the patient. As expected, changes in the segmental TrueKyphosis are probably related to the coupling effect of derotation and coronal correction of the deformity more than actual kyphosis generation. A long-term follow-up of this population will be needed to appreciate this technique’s potential.

## O9 Numerical simulation to evaluate 3D correction of pediatric scoliosis over 2 years with Anterior Vertebral Body Growth Modulation fusionless device

### Nikita Cobetto^1,2^, Stefan Parent^2,3^, Carl-Éric Aubin^1,2,3^,

#### ^1^Department of Mechanical Engineering, Polytechnique Montréal, Montreal, Quebec, H3C 3A7 Canada; ^2^Research Center, Sainte-Justine University Hospital Center, Montreal, Quebec, H3T 1C5 Canada; ^3^Faculty of Medicine, Department of Surgery, Université de Montréal, Montréal, Quebec, H3C 3J7 Canada

**Introduction**

Anterior Vertebral Body Growth Modulation (AVBGM) is a recent fusionless technique aiming to correct scoliosis during growth. Vertebrae are instrumented on the curve’s convex side using a flexible cable attached to vertebral screws to apply compressive forces, modulating growth. Surgical planning remains empirical regarding instrumented levels and cable tensioning. A patient-specific finite element model (FEM) of pediatric scoliosis integrating growth was previously developed to simulate the AVBGM installation, growth and growth modulation effect, and was tested on a few cases to assess the feasibility.

**Objectives**

To further assess the FEM, and biomechanically evaluate 3D correction of AVBGM, as well as the simulated stresses applied on growth plates.

**Methods**

75 patients instrumented with AVBGM were consecutively recruited. A patient-specific FEM was generated from the patient’s 3D reconstruction using calibrated bi-planar radiographs. The FEM allows the simulation of the AVBGM device installation, as well as the growth and growth modulation effect due to gravitational and AVBGM compressive forces. Intra-operative lateral decubitus position was simulated to evaluate the intra-operative positioning relative contribution on immediate post-operative correction. Stresses applied on growth plates and intervertebral discs were computed and correction indices were measured in the coronal, sagittal and transverse planes for immediate, 1 year and 2 years’ post-operative results (Cobb angles, kyphosis/lordosis angles, apical axial rotation, spinal height T1-L5).

**Results**

The simulated lateral decubitus reduced Cobb angles on average by 30% over an average total correction of 43% for immediate post-operative correction, showing that the majority of curve correction was achieved by intra-operative positioning. Immediate post-operative Cobb angles were simulated within 4° compared to actual results, and 5° for kyphosis/lordosis angles and axial apical rotation. The difference between convex and concave sides forces applied on the apical vertebra was decreased on average by 39% on growth plates and 46% on intervertebral discs in asymmetric loadings. For patients with 1 year (51 patients) and 2 years (40 patients) follow-up, correction results were predicted within 5° for Cobb angles, 5° for kyphosis/lordosis angle, 4° for apical axial rotation and <8 mm (± 2%) for spinal height.

**Conclusions and significance**

This study demonstrated the clinical usefulness of numerical simulations for the AVBGM fusionless device to simulate its 3D corrective effect with clinically relevant accuracy and within the precision range of clinical measurements. By allowing pre-operative simulation on the AVBGM corrective effects on immediate and 2 years post-surgery correction, the FEM could be used to rationalize surgical planning.

## O10 Sagittal pelvic morphology during human evolution: A perspective on different hominoidae

### Tom P. C. Schlösser^1^, Michiel M. A. Janssen^1^, Tom Hogervorst^2^, Tomaž Vrtovec^3^, John de Vos^4^, F. Cumhur Öner^1^, René M. Castelein^1^

#### ^1^Department of Orthopaedic Surgery, University Medical Center Utrecht, Utrecht, the Netherlands; ^2^Department of Orthopaedic Surgery, Haga Hospital, The Hague, the Netherlands; ^3^Faculty of Electrical Engineering, University of Ljubljana, Ljubljana, Slovenia; ^4^Naturalis Biodiversity Center, Leiden, the Netherlands

**Introduction**

The importance of sagittal spino-pelvic alignment for posture and upright human spinal biomechanics, and its role in the etio-pathogenesis of different spinal pathologies is well recognized. In human evolution, morphological changes of the pelvis are believed to be a crucial step forward towards pertinent bipedalism. Objective: To provide a comparison of sagittal pelvic morphology within the superfamily of *Hominoidea*.

**Methods**

Computed tomographic images of four human-like primates from the superfamily of *Hominoidae* (apes) and two early *Hominins* (*Australopithecus afarensis* and *Homo ergaster*) were acquired and compared to modern *Homo sapiens*. Semi-automatic analyses were performed, using previously validated image processing techniques, to measure the ischio-iliac angle and the pelvic incidence.

**Results**

Ischio-iliac angle and pelvic incidence increased from 1° and 27° in human-like primates, to 22° and 33° in early *Hominins*, to 19° and 39° in modern human children and 26° and 48° in modern human adults, respectively.

**Conclusion and significance**

By comparison of sagittal pelvic morphology within the superfamily of *Hominoidea,* we have the perspective that in the course of human evolution, development of a lordosis between the ischium and ilium, and the consequent increase in pelvic incidence allowed for ambulation in a fully upright position while maintaining the lever arm of the ischial musculature*.*

## O11 A novel scoliosis instrumentation using special superelastic nickel-titanium shape memory alloy spinal rods can result in equivalent correction as conventional rods but with less stress at bone-implant interface: A biomechanical evaluation through simulations

### Xiaoyu Wang^1,2^, Kelvin Yeung^3^, Jason Pui Yin Cheung^3^, Johnson Lau^3^, Weichen Qi^3^, Kenneth Man-Chee Cheung^3^, Carl-Eric Aubin^1,2^

#### ^1^Department of Mechanical Engineering, Polytechnique Montreal, Montreal (Quebec), Canada; ^2^Sainte-Justine University Hospital Center, Montreal (Quebec), Canada; ^3^Department of Orthopaedics and Traumatology, The University of Hong Kong, Hong Kong SAR, China

##### **Correspondence:** Kenneth Man-Chee Cheung (cheungmc@hku.hk); Carl-Eric Aubin (carl-eric.aubin@polymtl.ca)

**Introduction**

A nickel-titanium (SNT) rod was engineered to be highly malleable below 20°C and maximally superelastic after austenite phase transformation at 37°C. Its shape-memory properties enable progressive correction during austenite phase transformation. Its strain-stress curve has a wide superelastic plateau enabling relatively constant corrective forces and post-instrumentation correction improvement from tissues relaxation. A pilot clinical trial using SNT rod in AIS instrumentation has been completed with comparable efficacy results to conventional titanium rods, but little biomechanical investigation has been done on how to maximally utilize the shape-memory and superelastic properties for deformity correction.

**Objectives**

To investigate the correction and stress level at the bone-implant interface and in the shape-memory SNT rod in AIS instrumentation.

**Methods**

The computational study was performed using biomechanical models of 7 thoracic AIS patients. The main thoracic Cobb angles (MT) were 43°–76° and thoracic kyphosis (TK) 7°–12°. We alternately simulated instrumentations using pedicle screws and SNT (at 18°C and 37°C), titanium (Ti) and cobalt-chrome (CoCr) rods. The independent variables were the rod diameter (5.5 and 6 mm), pre-designed rod bending angle (30° and 40°) and rod derotation angle (90° and 110°). The simulated correction maneuvers were rod reduction and rod derotation. For a total of 224 virtual instrumentations, the resulting MT and forces in the rods and at bone-screw interfaces were computed and compared.

**Results**

Simulated MT correction (curve reduction) by SNT rod was on average -12° (before the austenite phase transformation) and improved to -26° (after the phase transformation), vs. -31° (Ti rod) and -34° (CoCr rod). The associated bending moments throughout the rods were 1.4, 3.4, 4.6, and 6 Nm on average, while the average bone-screw forces were 19, 45, 62 and 74 N. Larger SNT rod diameter or pre-designed bending angle increased TK by about 5° without changing MT correction, but larger SNT rod derotation improved the MT correction by -5°. Peak bending moments in SNT rods were 7–16 Nm; only in SNT rods of larger diameter, rod bending and derotation angles, were peak bending moments (11–16 Nm) above the threshold bending (8 and 10 Nm for 5.5 and 6 mm diameter SNT rods) for the SNT rod to work in the superelastic domain.

**Conclusions and significance**

This study elucidated scoliosis correction mechanisms using SNT rods and demonstrated that SNT rods allowed lower forces during initial rod reduction and derotation, and provided *in situ* progressive correction through the subsequent austenite phase transformation. With the selection of certain SNT rod parameters, it is possible to achieve similar MT correction as with Ni and CoCr rods, but with the advantage to work in the superelastic plateau of their strain-stress curve, which could possibly provide further post-instrumentation correction after tissue relaxation.

## O12 Unique correlation pattern between bone qualities and handgrip strength in adolescent idiopathic scoliosis (AIS) girls

### Ka-Yee Cheuk^1,2,3^, Vivian WY Hung^1,4^, Fiona WP Yu^1,2,3,4^, Lyn LN Wong^1,2,3,4^, Wayne YW Lee^1,2,3^, Jack CY Cheng^1,2,3,4^, Tsz-Ping Lam^1,2,3,4^

#### ^1^Department of Orthopaedics and Traumatology, The Chinese University of Hong Kong, Hong Kong; ^2^SH Ho Scoliosis Research Laboratory, Faculty of Medicine, The Chinese University of Hong Kong, Hong Kong; ^3^Joint Scoliosis Research Center of the Chinese University of Hong Kong and Nanjing University, The Chinese University of Hong Kong, Hong Kong; ^4^Bone Quality and Health Centre, Department of Orthopaedics and Traumatology, The Chinese University of Hong Kong, Hong Kong

**Introduction**

Previous studies reported the association between adolescent idiopathic scoliosis (AIS) and low bone mass and poor bone health. Cross-talk between bone and muscle was recently described receiving wide attention and can potentially shed lights on the nature of poor bone health and its role in the etiopathogenesis of AIS. In spite of this and to the best of our knowledge, studies on both bone and muscle parameters and their correlation in AIS have not been reported in the literature.

Objectives: To investigate the correlation between handgrip strength and bone qualities (volumetric bone mineral density (vBMD), bone geometry, trabecular bone micro-architecture) and bone mechanical properties in girls with AIS versus age- matched normal girls.

**Methods**

142 AIS girls and 247 non-AIS girls aged 12-14 years old were recruited. Maximum handgrip strength was measured with a standard dynamometer and bone qualities of non-dominant distal radius were measured by high-resolution peripheral quantitative computed tomography (HR-pQCT). Bone mechanical properties were assessed by Finite Element Analysis (FEA). Partial correlation was used to control confounding from age, arm span and weight in data analysis.

**Results and discussions**

In non-AIS girls and after adjustment for confounders, handgrip strength was positively correlated with bone area (R = 0.17, p = 0.01 in cortical area and R = 0.24, p < 0.001 in trabecular area) and bone mechanical properties (R = 0.25 in stiffness and R = 0.24 in failure load, both p < 0.001), but not correlated with vBMD and trabecular bone micro-architecture. In contrast for AIS girls, handgrip strength was positively correlated with cortical area (R = 0.22, p < 0.01), cortical thickness (R = 0.19, p=0.03), and bone mechanical properties (stiffness, failure load and apparent modulus, R = 0.22 – 0.33, all p < 0.05) but negatively correlated with structure model index (R = -0.18, p = 0.04), which reflects the trabecular plate-rod ratio.

**Conclusions and significance**

This study indicated unique correlation patterns between bone qualities and handgrip strength in AIS as distinct from controls. This finding suggests the characteristic bone-muscle cross talk in AIS could play an important role in the etiopathogenesis of AIS. Further longitudinal studies to investigate if handgrip strength can be used as a surrogate for bone parameters and the role of handgrip strength in predicting curve progression in AIS are warranted.

**Acknowledgement**

This study is supported by RGC of HKSAR (468809 & 468411).

## O13 Does Walter Reed Visual Assessment Scale reflect actual scapular positional fault?

### Gozde Yagci^1^, Elif Camci^1^, Yavuz Yakut^2^

#### ^1^ Physiotherapy and Rehabilitation Department, Hacettepe University, Ankara, Turkey; ^2^ Physiotherapy and Rehabilitation Department, Hasan Kalyoncu University, Gaziantep, Turkey

**Introduction**

In Idiopathic scoliosis, improper mechanical forces acting on the spine lead to biomechanical and physiological alterations along the trunk segment. Two or three dimensional surface topographic methods or visual assessment scales based on the projection and observation of the back are commonly used methods for evaluating various aspects of the scoliotic deformity. But there is no clear evidence that the observational trunk deformity scales actually reflect the deformity, itself.

**Objectives**

The main objective of this prospective investigation was to evaluate the ability of the Walter Reed Visuel Assessment Scale (WRVAS) to reflect scapular positional fault in patients with scoliosis.

**Methods**

Sixty idiopathic scoliosis patients with a mean age of 14.5 (range, 10 to 17 years) were included in the study. The patients divided into four groups (15 person for each) represent degrees of severity of the scapular rotations on WRVAS: level 2, level 3, level 4 and level 5 (maximum severity). Scapular rotations on the WRVAS scale were compared with three-dimensional scapular kinematics assessment by electromagnetic tracking system. The position and orientation of the scapula including internal/external rotation, posterior/anterior tilting, and downward/upward rotation was evaluated. Kruskal Wallis and Bonferonni-Dunn post hoc tests were performed for statistical comparisons.

**Results and discussion**

The mean magnitudes of the thoracic curve and lumbar curve were 31.1° ± 8.9 and 28.5° ± 9.1, respectively. The main findings are that convex scapular internal/external rotation showed changes between level 2 and level 4 groups (p=0.001). Concave scapular upward/downward rotation showed changes between both level 2 and level 4 (p<0.001), and level 2 and level 5 (p<0.001) groups. There was no difference between any two groups for other scapular parameters.

**Conclusion and significance**

The present results shows that measurement of scapular rotation with WRVAS may not reflect actual scapular positional fault. One thing is that the scale seems to include only convex side scapular rotation assessment, however altered scapular position was obtained for concave side, as well. Other thing that there was no difference obtained between groups in terms of three-dimensional scapular kinematics in resting, except for level 2 and level 4 severity groups. Our data suggest that the subjectively derived WRVAS may not be used as a surrogate for the objectively determined scapular positional fault with three-dimensional kinematic analysis in idiopathic scoliosis.

## O14 A pilot MR study of thoracic vertebral morphology in IS

### Ayesha Maqsood, Sohaib Hashmi, Matthew Hartwell, John F. Sarwark^4^

#### Department of Surgery, Ann & Robert H. Lurie Children’s Hospital of Chicago, Chicago, IL, 60611, USA

##### **Correnspondence:** John F. Sarwark (jsarwark@luriechildrens.org)

**Introduction**

Idiopathic scoliosis (IS) is a structural deformity of the human spine. Males and females are affected by the disease but the classic case is an adolescent female during puberty, who is healthy with normal development until the induction of scoliosis. It is theorized that the immature developing spine is susceptible to deformation during growth.^1^ This study evaluates early biomechanical factors in the induction phase of IS.

**Objective**

The main hypothesis of this pilot study is: there will be observable differences in sagittal harmony and vertebral morphology between normal and early IS affected vertebra on MR imaging.

**Methods**

The criteria for controls and those with early onset IS were established in order to conduct a search for prospective patients. IS group: Inclusion criteria- subjects with early on-set scoliosis diagnosis (diagnosed at or before the age of 12 years), a Cobb angle under 40° and no neurosurgical abnormality. Control group: Inclusion criteria- no known musculoskeletal disorders and age matched to patients in the IS group. After the search was conducted, study team members screened the patient’s medical records and imaging. Twenty patients, ten for the IS and ten for the control groups were identified and their XR and MR images were qualitatively analyzed. A record of the sagittal balance, sagittal harmony and vertebral morphology were made for IS affected and control patients.

**Results and conclusions**

The sagittal balance is negative in nearly all IS patients; it is consistently positive in control patients. In the IS group, spinal harmony is consistently lost; it is consistently maintained in the control group. Sagittal wedging was not seen in the control group but was observed in two patients in the IS affected group; aged 8 and 12 years old. In all cases of control patients, the vertebral corners were perfectly symmetrical on coronal and sagittal images. In IS patients the vertebral morphology remained intact until about 5 years of age and was changed from 6 years up to 12 years old. Vertebral corner morphology and sagittal wedging indicate that the observed phenomena may not present at an early age but is seen later in IS progression. The deformities observed in the patients with IS are regional and not global across the spine.

**Significance**

We propose a Heuter-Volkmann effect in the induction phase of early IS. In susceptible individuals, the hypothesis that stress from vertical loading forces on the middle column vertebral end plates results in growth irregularities is supported.

**References**

1. Kouwenhoven JW, Smit TH, van der Veen AJ, Castelein RM, et al. Effects of dorsal versus ventral shear loads on the rotational stability of the thoracic spine: a biomechanical porcine and human cadaveric study. Spine. 2007;32(23):2545-50.

## O15 The impact of scapular dyskinesis on upper extremity functions in idiopathic scoliosis

### Gozde Yagci^1^, Çiğdem Ayhan^1^, Yavuz Yakut^2^

#### ^1^Physiotherapy and Rehabilitation Department, Hacettepe University, Ankara, Turkey; ^2^ Physiotherapy and Rehabilitation Department, Hasan Kalyoncu University, Gaziantep, Turkey

**Introduction**

Proper scapular biomechanics have been shown to be necessary for normal shoulder function during functional activities. Altered scapular kinematics was reported in patients with idiopathic scoliosis (IS) in several studies; however, there is no study about the relationships between scapular dyskinesis and upper extremity functions in individuals with IS.

**Objectives**

The aim of this study was to investigate upper extremity functions in IS patients, who have scapular dyskinesis.

**Methods**

Adolescents with moderate IS were evaluated with Observational Clinical Assessment of Scapular Dyskinesis method during shoulder elevation by a blinded evaluator, first. The evaluator categorized the scapular motion of all subjects to determine the presence of dyskinesis and 60 adolescents with scapular dyskinesis (mean age: 14.1 ± 1.8 years) included in this study. Disabilities Arm Shoulder and Hand (Quick-DASH) questionnaire was used for the assessment of upper limb functions.

**Results and discussion**

Among those patients with scoliosis 23 had double curves (38.35%) and 37 had single curves (61.7%). Average Cobb angle was 27.6° ± 6.5° (20° to 45°) for thoracic region, 25.2° ± 6.5° (10° to 40°) for lumbar region and 28.1° ± 7° (20° to 41°) for TL region. The For the DASH questionnaire, the mean score was 9.5 ± 7.3 points out of 100 (0 to 32.0). According to DASH cut off scores to determine the severity of upper extremity functional limitation, 44 patients (73.3%) had no difficulties in activities of daily living, whereas 16 patients (26.7%) had some. The difficulties were primarily in carrying weight or a backpack, washing back, studying and recreational activities, which require little effort.

**Conclusion and significance**

This study shows that IS patients, who have scapular dyskinesis are not likely to have upper extremity functional limitations during daily life. Further studies are warranted to determine whether observed scapular kinematic alterations in IS affect upper extremity functions in long term, such as decades.

## O16 Calcium and vitamin D for controlling curve progression in adolescent idiopathic scoliosis – further review with Finite Element Analysis (FEA) for a randomized double-blinded placebo-controlled trial

### Tsz Ping Lam^1^, Benjamin Hon Kei Yip^2^, Fiona Wai Ping Yu^1^, Nelson Leung Sang Tang^3^, Kenneth Kin Wah To^4^, Kwong Man Lee^5^, Wayne YW Lee^1^, Bobby Kin Wah Ng^1^, Alec Lik Hang Hung^1^, Yong Qiu^6^, Jack Chun Yiu Cheng^1^

#### ^1^SH Ho Scoliosis Research Laboratory, Joint Scoliosis Research Center of the Chinese University of Hong Kong and Nanjing University, Department of Orthopaedics and Traumatology, The Chinese University of Hong Kong, Prince of Wales Hospital, Shatin, Hong Kong SAR, China; ^2^ The Jockey Club School of Public Health and Primary Care, The Chinese University of Hong Kong, Hong Kong SAR, China; ^3^ Department of Chemical Pathology and School of Biomedical Sciences, Faculty of Medicine, The Chinese University of Hong Kong, Hong Kong SAR, China; ^4^ School of Pharmacy, The Chinese University of Hong Kong, Hong Kong SAR, China; ^5^ Lee Hysan Clinical Research Laboratories, The Chinese University of Hong Kong, Shatin, Hong Kong SAR, China; ^6^ Joint Scoliosis Research Center of the Chinese University of Hong Kong and Nanjing University, Spine Surgery, The Affiliated Drum Tower Hospital of Nanjing University Medical School, Nanjing, China

**Introduction**

Adolescent Idiopathic Scoliosis (AIS) is associated with low bone mass which has been reported to be an independent and significant prognostic factor for curve progression in AIS. Given that AIS subjects have low serum 25(OH)Vit-D levels and low dietary calcium (Ca) intake, it will be logical to evaluate whether Ca plus Vit-D (Ca+Vit-D) supplementation could improve bone health and prevent curve progression in AIS.

**Objective(s)**

To evaluate if Ca+Vit-D supplementation could improve bone strength and prevent curve progression in AIS.

**Method(s)**

This was a randomized double-blinded placebo-controlled trial on 330 AIS girls (11-14 years old, femoral neck aBMD Z-scores<0 and Cobb angle>15°) randomized to Group1 (placebo), Group2 (600mg Calcium+400IUVit-D3/day) and Group3 (600mg Calcium+800IUVit-D3/day). Treatment duration was 2-yr. At baseline and 24-month, Finite Element Analysis(FEA) on HR-pQCT parameters, serum 25(OH)Vit-D and dietary calcium intake were assessed. Evaluation of curve progression defined as Cobb increase ≥ 6° was conducted according to SRS guidelines at the Latest Follow-up.

**Result(s) and discussions**

270(81.8%) subjects completed the study. The increase in FEA bone strength parameters (stiffness (kN/mm), failure-load (N) and apparent-modulus (MPa)) at 24-month were significantly greater in the Treatment Group (p<0.05). At the Latest Follow-up (N=132), 21·7% in Group3 and 24·4% in Group2 had curve progression ≥ 6° as compared with 46·7% in Group1 (p<0.05). Increase in FEA parameters, namely failure load and apparent modulus, were significant protective factors against curve progression (p<0.05). Ca+Vit-D supplementation was more effective in preventing curve progression for those with low baseline serum 25(OH)Vit-D levels ( ≤ 50nmol/L).

**Conclusion(s) and significance**

The results provide strong evidences that daily 600mg calcium+400/800IUVit-D3 improves bone strength and prevents curve progression in female AIS subjects with low bone mass especially for those with low baseline 25(OH)Vit-D levels and hence can be considered as a therapeutic option for preventing curve from progression to bracing or surgical thresholds.

This study was partially funded by an Investigator-initiated Research Grant from Pfizer Inc.

## O17 Comparison of the change in isometic trunk rotation torque from morning to evening in adolescents with and without idiopathic scoliosis

### Fatemeh Aslanzade, Eric Parent, Brian MacIntosh, Jalal Abodarda

#### University of Alberta, Edmonton, Canada

**Introduction**

The etiology of Adolescent Idiopathic Scoliosis (AIS) remains mostly unknown which limits our ability to develop treatments targeting the cause. Nevertheless, there are numerous theories regarding the pathogenesis of AIS or curve progression. A rotational strength weakness in rotation to the concave side has been shown in patients with AIS but it is unknown if a difference in fatigability exists between patients and healthy controls.

The objective of this study was to compare the change in trunk rotation torque toward the convex and concave side from the morning to the evening between adolescents with AIS and matched healthy controls.

**Methods**

Nine consecutive healthy adolescent females with AIS were recruited from the Edmonton Scoliosis clinic. Nine consecutive healthy adolescent females responding to recruitment ads were matched as controls to participants with scoliosis for age (1±year) and BMI (±5kg/m^2^). Approval was obtained from the local ethics board and consent from the participants. Two 5-seconds maximal voluntary isometric trunk rotation strength efforts in neutral sitting position turning toward the convex and concave sides were recorded using a Biodex Multi-joint System with the trunk rotation attachment. Testing was repeated in the morning and the evening. The peak rotation torque value for each of the two trials at each time point were extracted using Labchart and averaged. Evening values were substracted from morning values to calculate MVC change. Changes in rotation torque values were compared using a mixed-model ANOVA using group as between-subject with side (convex vs concave) as within-subject factors. LSD tests were used for post-hoc comparisons. SPSS 24.0 was used with an alpha level at p < 0.05.

**Result and discussion**

Seven participants with AIS had a right thoracic and two a left lumbar major curve. Their mean Cobb angle was 23±7^o^. Matched participants presented a mean age of 14±2.6 years, and a mean BMI of 48±6.7 kg/m^2^. The mean difference between the morning and evening session for the trunk rotation Torque towards the convex side was an increase of 2.99±5.61Nm/Kg for scoliosis group and of .45±3.77Nm/Kg for controls. The mean morning to evening difference in trunk rotation torque towards the concave side was an increase of 2.08±4.90 Nm/Kg and 1.10±4.11Nm/Kg for the scoliosis and control groups, respectively.

Changes in MVC from morning to evening were significantly different between concave and convex sides in the scoliosis group (Group by Side interaction Greehouse-Geisser F 1,9.21=9.21, p<.05). No other pairwise comparisons were significant.

**Conclusion**

Participants with AIS became weaker when rotating toward their concave side from the morning to the evening while the opposite occured on the convex side. Results indicate that scoliosis subjects had symmetrical strength in the morning when they were rotating toward their concave or convex side, but, at the end of day they were significantly weaker while rotating toward concave side. These findings are in agreement with Mooney’s finding.

## O18 Cheneau brace treatment: comparison of compliant versus non compliant patients

### Krzysztof Korbel, Piotr Janusz, Malgorzata Kotwicka, Tomasz Kotwicki

#### Department of Spine Disorders and Pediatric Orthopedics, University of Medical Sciences, Poznan, Poland

**Introduction**

Rigid brace combined with specific physiotherapy is an accepted standard treatment for moderate idiopathic scoliosis (IS). Results of scoliosis brace treatment depend on (1) indication, (2) brace quality and (3) patient’s compliance. Noncompliance during brace wearing worsens the outcome.

**Objective**

To analyse the outcome of Cheneau brace treatment for IS according to patients’ compliance level.

**Methods**

Forty-eight consecutive girls with IS were included, all fulfilling SRS brace study criteria, all treated by one physician and braces produced by the same manufacturer. They were divided into compliant and noncompliant group. The compliant group comprised fulltime brace wearing patients (at least 20/24h including brace wearing at school). The noncompliant group comprised patients who were not wearing the brace at school however, the brace using time was at least 12/24h. The compliance of each patient was assessed consecutively during the whole treatment course. The outcome of compliant versus noncompliant group was compared at two years after brace weaning.

**Results**

There were 31 girls in the compliant group versus 17 girls in the noncompliant group. At start the groups were comparable according to: age (12.5 years vs. 12.1 years), age of menarche (12.8 years vs. 12.5 years), primary curve Cobb angle (30.1º vs. 32.2º), age at the end of brace wear (16.5 years vs. 16.1 years), and time of brace wear (4.0 years vs. 4.0 years)**.** The compliant group revealed significantly bigger angle of trunk rotation (ATR) at the primary curve level: 11.1º vs. 8.8º, p=0.0371.

The final primary curve Cobb angle was significantly smaller in the compliant group (34.9º vs. 42.2º, p=0.0197). The Cobb angle progression was 4.8º in the compliant versus 10.0º in the noncompliant group. Cobb angle progression of more than 5º was observed in 35.5% versus 58.8% of patients, respectively. At the final follow up, the ATR was comparable in both groups (9.4º vs. 10.6º), however due to initial values, there was a significant ATR improvement in the compliant group versus worsening in the noncompliant group. Surgery was recommended in 12.9% of compliant versus 35.3% of noncompliant patients.

**Conclusion**

Patients presenting similar deformity and receiving similar Cheneau brace treatment revealed significant difference of outcome depending on the compliance level. Compliant patients revealed smaller progression of Cobb angle than noncompliant patients. Compliant patients revealed ATR improvement, whereas noncompliant patients revealed ATR increase. Surgical indication was almost three times more frequent in the noncompliant patients.

**Significance**

The study confirms importance of compliance during brace treatment for idiopathic scoliosis.

## O19 Outcome of Cheneau brace treatment for idiopathic scoliosis in adolescents

### Krzysztof Korbel, Piotr Janusz, Paweł Główka, Tomasz Kotwicki

#### Department of Spine Disorders and Pediatric Orthopedics, Poznan University of Medical Sciences, Poznan, Poland

**Introduction**

Rigid brace treatment combined with specific physiotherapy is accepted as standard treatment for moderate idiopathic scoliosis (IS). Aims of conservative treatment are: to stop curvature progression, to reduce the curvature angle if possible, to reduce indications for surgical treatment, to improve respiratory function, to reduce pain if present, to improve the body posture aesthetics.

**Objective**

The aim of this study was to analyse the outcome of Cheneau brace treatment in IS patients at high progression risk according to Scoliosis Research Society (SRS) criteria.

**Methods**

Study design: retrospective analysis of prospectively collected database, consecutive cases, intend-to-treat analysis. SRS inclusion criteria according were used: females, diagnosis of IS, age > 10 years, Cobb 25-40 degrees, Risser 0-2, no previous treatment, girls not older than one year after menarche, at least two-year follow-up after brace weaning. Forty-eight girls were included. The mean age at the beginning of bracing was 12.3 ± 1.3 years and at the brace weaning 16.3 ± 1.4 years. The initial major curve Cobb angle was 31.3º ± 4.3, the initial angle of trunk rotation (ATR) was 10.3º ± 3.7 and the initial Risser sign median was 0. The patients were treated by one team supervised by one physician (last author) while the results were evaluated by one independent observer (first author). Recommendation of full time brace wearing (at least 20h per day) was given to all patients.

**Results**

The final examination performed two years after the brace weaning revealed as follows: the mean Cobb angle 37.5º ± 10.6, the mean ATR 9.8º ± 3.8, the Risser sign median 5. The comparison of the final examination to the initial examination revealed: Cobb angle decreased more than 5º in 6.3%, stabilized ± 5.0° in 50%, increased more than 5º in 43.7% and increased more than 10º in 29.2% of patients. The mean ATR decreased more than 3.0º in 18.8% of patients, stabilized ± 3.0° in 60.4%, and increased more than 3.0º in 20.8% of patients. 23% of patients reached the 45 degrees of Cobb angle while 16.7% of patients reached 50 degrees Cobb. Surgical recommendation received 20.8% of patients.

**Conclusion**

The natural history of idiopathic scoliosis at high risk of progression was modified by Cheneau brace treatment. However, 20.8% patients still received surgical recommendation, those with initially bigger curves or less compliant. More than half of patients revealed stabilization or improvement of spine curvature. Rigid brace treatment decreased or stabilized rib hump magnitude in more than 80% of patients.

**Significance**

The study confirms the effectiveness of treatment of progressive idiopathic scoliosis with a rigid brace.

## O20 Quality of Life of adult patients with idiopathic scoliosis with a curvature above 35 degrees

### Anne C. Brandwijk, Johan L. Heemskerk, Mark C. Altena, Diederik H. R. Kempen

#### Orthopedic Surgery, OLVG Hospital, Amsterdam, 1091AC, Netherlands

##### **Correspondence:** Anne C. Brandwijk (a.c.brandwijk@olvg.nl)

**Introduction**

Although many children with idiopathic scoliosis are asymptomatic during childhood, children with progressive scoliosis are treated to alter the natural history of the deformity and prevent future problems in adulthood. Previous studies on the natural history of moderate to severe idiopathic scoliosis (IS) show contradictory results. Whereas early studies reported an increased incidence in back pain and disability in severe curves, later studies reported no difference compared to age matched controls in adult patients. The results of these studies are based on questions regarding back function, quality of life and social life, since no modern quality of life (QoL) questionnaires were available at that time.

**Objective**

The aim of our study is to examine the QoL of adult patients with IS with a curvature above 35 degrees using modern QoL questionnaires

**Methods**

Patients with IS, born between 1945 and 1981 who have consulted OVLG Hospital during childhood with curves above 35 degrees were selected from the scoliosis database, traced and asked to participate. Patients were either untreated or treated with a brace. Patients willing to participate received the following questionnaires focussing on QoL; RAND- 36 Survey and EQ-5D-3L. Outcomes of the RAND-36 were compared with a nationwide reference cohort (N=1742) with a mean age of 47,6 years old. Significance has been defined as P<0.05.

**Results**

From the 210 eligible patients, 114 were traced and willing to participate. Their average age was 49.3±7.4. Of the 114 patients, 71.1% were treated with a brace and 28.9% were untreated. The mean Cobb angle at the last consultation was 45.1°±10.55. Although all scores of the eight RAND-36 subdomains were slightly lower in the scoliosis group, only the vitality (p<0.001) and mental health (p<0.019) subdomains were significantly different compared to the nationwide cohort. The eight subdomains of the RAND-36 can be divided into two domains (physical and mental). There was no significant correlation between the overall physical/mental domains and Cobb angle (Pearsons correlation coefficient of -0.178 and -0,114 respectively). A Pearsons correlation between the size of the Cobbs angle and total score of the EQ-5D-3L shows a weak correlation of -0.239 (p=0.01).

**Conclusion**

Despite lower scores on the eight subdomains of the RAND-36, IS patients with curves above 35 degrees only showed significantly lower scores on the vitality and mental health subdomains compared to a nationwide reference cohort. There was a weak correlation between the total score of the EQ-5D-3L and Cobb angle.

## O21 Sagittal spinal profile and pelvic parameters in adolescent idiopathic scoliosis before and after posterior spinal fusion: comparison with normal controls by EOS radiography

### Kwong Hang Yeung^1^, Zongshan Hu^1^, Tsz Ping Lam^2^, Bobby Kin Wah Ng^2^, Jack Chun Yiu Cheng^2^, Winnie Chiu Wing Chu^2^

#### ^1^Department of Imaging and Interventional Radiology, Faculty of Medicine, The Prince of Wales Hospital, The Chinese University of Hong Kong, Shatin, Hong Kong SAR; ^2^Department of Orthopaedics and Traumatology, Faculty of Medicine, The Prince of Wales Hospital, The Chinese University of Hong Kong, Shatin, Hong Kong SAR

**Introduction**

Adolescent idiopathic scoliosis is a 3D spinal deformity. Some patients with progressive curve despite bracing require surgical treatment. After operation, the correction of coronal deformity is conventionally assessed by reduction in Cobbs angles; surgical correction of sagittal profile in AIS patients however, is less emphasized and evaluated. Suboptimal sagittal alignment may cause long term complications such as pain, aggravated spinal degeneration or minor spinal fracture. It is therefore important to evaluate the sagittal spinal and pelvic alignment in AIS subjects too.

**Objectives**

(1) To identify the difference of sagittal profile and pelvic parameters between AIS and young healthy

subjects of similar age.

(2) To see whether there is improvement in sagittal and pelvic alignment in early post op and follow up period.

**Methods**

The study group consisted of 20 AIS with a primary thoracic curve requiring surgical treatment (all with Risser sign 4 to 5, indicating almost or already reached cessation of growth) and 20 healthy young subjects with the mean age of 18.3±5.0 and 24.7±2.8 years, respectively. All subjects underwent EOS biplanar radiographs at baseline. AIS subjects underwent EOS imaging at two additional time points: within one- month post op, and a follow up study within 3-12 months post op. Cobb angle and multiple sagittal spinopelvic parameters were measured, which included cervical lordosis (CL), thoracic kyphosis (TK), lumbar lordosis (LL), sacral slope (SS), pelvic incidence (PI), pelvic tilt (PT), sagittal vertical axis (SVA) and 2 pelvic obliquity (PO). Independent t-test/ Mann-Whitney test was used for AIS and non-AIS healthy groups comparison for data with/ without normal distribution. A pairwise comparison with the Post Hoc analysis of Bonferroni correction was used for intragroup comparison for AIS subjects at 3 different time points. Spinopelvic parameters were correlated with Cobb angle by pearson correlation test.

**Results & discussion**

There was significant difference (P<0.05) in all parameters (except pelvic tilt and sagittal vertical axis) between pre-op AIS and control groups. Cobb angles were significantly reduced after operation without rebound at follow up. For the sagittal and pelvic parameters, at post op 1-month assessment, lumbar lordosis (LL), pelvic incidence (PI) and sacral slope (SS) reversed to similar values comparable to those in controls without significant statistical difference. However, these parameters showed rebound and became significantly different from controls again at follow up. Despite improvement, the cervical lordosis (CL), thoracic kyphosis (TK) and pelvic obliquity (PO) remain significantly different from controls throughout the early post op and delayed follow up scanning. Significant correlation was found between Cobb angle with CL, TK and PO at baseline.

**Conclusion & significance**

Our results reaffirm the presence of significant reversed cervical lordosis, thoracic hypokyphosis, lumbar hyperlordosis in AIS when compared to normal subjects. Despite an excellent improvement in coronal profile, most of the sagittal spinopelvic parameters remain significantly different from normal controls after the surgery including cervical hypolordosis, thoracic hypokyphosis and pelvic obliquity. Some parameters showed initial improvement but rebound.

## O22 Comparison of spinal morphology in pre-operative adolescent idiopathic scoliosis patients who underwent simultaneous erect EOS and prone CT

### Kwong Hang Yeung^1^, Steve Cheuk Ngai Hui^1^, Babak Hassan Beygi^1^, Tsz Ping Lam^3^, Bobby Kin Wah Ng^2^, Rob C. Brink^4^, Man Sang Wong^3^, Jack Chun Yiu Cheng^2^, Winnie Chiu Wing Chu^2^

#### ^1^Department of Imaging and Interventional Radiology, Faculty of Medicine, The Prince of Wales Hospital, The Chinese University of Hong Kong, Shatin, Hong Kong SAR; ^2^Department of Orthopaedics and Traumatology, Faculty of Medicine, The Prince of Wales Hospital, The Chinese University of Hong Kong, Shatin, Hong Kong SAR; ^3^Department of Biomedical Engineering, The Hong Kong Polytechnic University, Hung Hom, Kowloon, Hong Kong, China; ^4^Department of Orthopaedic Surgery, University Medical Center Utrecht, Utrecht, The Netherlands

**Introduction**

CT provides accurate 3D reconstruction of a scoliotic spine but involves high radiation. A new biplanar radiography system (EOS) allow imaging of a scoliotic spine in physiological upright posture with significant lower radiation. However, EOS 3D reconstruction is based on statistical model with estimated axial profile. The reconstruction accuracy between two modalities should be tested. On the other hand, the 3D spinal parameters assessed by CT might be compromised by the prone positioning of the patients.

**Objectives**

1) To validate the accuracy of 3D measurement of various spinal parameters from EOS by parallel CT scanning using a scoliotic phantom.

2) To compare any difference in scoliotic parameters between erect and prone positions within the same AIS subjects, measured by EOS and CT, respectively.

**Methods**

A scoliotic phantom with a moderate-severe curve was scanned by both EOS and CT to validate the accuracy of axial reconstruction in EOS, which was automatically generated by the build-in software. 33 pre-op AIS subjects (18.0±4.0yrs) underwent CT in prone position and EOS in standing position on the same day. Scoliotic parameters included slenderness, axial intervertebral rotation, torsion, wedging of vertebrae, Cobb angle, kyphosis and lordosis acquired from simultaneous EOS and CT were compared using paired test. Parameters measured by EOS and CT were correlated using pearson correlation test.

**Results & discussion**

Results showed no significant difference in axial parameters measured by the two modalities for the phantom. In human subjects, significant difference (P<0.05) was observed between two different postures by CT and EOS respectively for lordosis and Cobb angles. No significant difference was found in kyphosis, slenderness, torsion, axial rotation and wedging. In general, measurement by EOS had higher statistical mean value than CT in all parameters. Strong linear correlations were found between CT and EOS for Cobbs angle, wedging of vertebrae, slenderness and torsion with the value of 0.924, 0.871, 0.860, 0.696, respectively.

**Conclusion & significance**

There is a reliable axial reconstruction of spinal structures by EOS as reflected by the rigid phantom. For human subjects in different postures, there is significant difference in Cobbs angle and lordosis but not in kyphosis, axial rotation and torsion. Latter three can be explained by restricted flexibility of the severe scoliotic curve at the thoracic levels by rib cage. The 3D deformity of the scoliotic spine is under-estimated by CT when AIS subjects are in prone position. We conclude that the 3D deformity of AIS can be accurately assessed by EOS in erect position with extremely low radiation exposure.

## O23 Reliability of the EOS 3D imaging system for adolescent idiopathic scoliosis patients status post-surgery

### Adam Miller^3^, Kenoma Anighoro ^1,3^, Channing Tassone^1,2,3^, John Thometz^1,2,3^, Xue-Cheng Liu^1,2,3^

#### ^1^Department of Orthopaedic Surgery, Medical College of Wisconsin, Milwaukee, WI, 53226, USA ; ^2^ Musculoskeletal Functional Assessment Center, Children’s Hospital of Wisconsin, Milwaukee, WI 53226, USA; ^3^ Medical College of Wisconsin, Milwaukee, WI, 53226, USA

#### **Correspondence:** Adam Miller; Xue-Cheng Liu

**Introduction**

The EOS biplanar x-ray imaging system provides a low-dose radiation option for serial imaging and 3D spinal modeling of children with Adolescent Idiopathic Scoliosis (AIS). Our previous study demonstrated a moderate to high reliability for EOS in AIS patients without surgery, however, posterior spinal instrumentation obstructs the anatomic landmarks that are critically important for 3D reconstruction of the spine. The reproducibility of 3D spinal modeling in AIS following surgery has not been thoroughly investigated to date.

**Objectives**

The purposes of the study were 1) to determine the intraclass correlation (ICC) for inter- and intra-observer measurements of Cobb Angle in the coronal plane (CA), Kyphosis (KY), Lordosis (LO) and Axial Vertebral Rotation (AVR); 2) to compare differences of spinal parameters between two examiners and two trials per examiner for AIS children status post-surgery.

**Methods**

Simultaneous AP and lateral standing x-ray images of twenty patients with mean age at time of surgery 14.8 ± 2.4 years (4 males, 16 females) were retrospectively selected and analyzed. These AIS patients underwent surgical correction of scoliotic deformity by pedicle screw direct vertebral rotation at a single institution between 2014 and 2016. 3D spine modeling was performed in the sterEOS software by two examiners. Each observer performed two independent trials for each patient’s post-surgery images. Manual identification of spinal and pelvic parameters allowed creation of a 3D spine model from T1 to L5. Intra- and inter-rater reliabilities for the two examiners was then assessed by calculating ICC. One sample t-test was performed to compare differences in spinal metrics.

**Results and conclusions**

The inter-rater ICC for post-surgery CA, KY, LO and AVR were 0.94, 0.97, 0.97 and 0.88, respectively. Similarly, the intra-rater ICC for examiner 1 for the same measures were 0.78, 0.79, 0.92, and 0.79, while examiner 2 showed 0.92, 0.90, 0.97, and 0.70, respectively. The average difference in CA, KY, LO, and AVR measurements between trials 1 and 2 for examiner 1 and 2 independently were less than 1.5º (P > 0.05). The average post-surgery difference in CA, KY, LO, and AVR between examiner 1 and 2 was less than 2.0º (despite P < 0.05.)

**Significance**

EOS 3D imaging provides a reproducible modality for analyzing three-dimensional spinal geometry post-surgery in AIS patients despite visual obstruction from spinal instrumentation following surgery. The reproducibility is excellent for both inter and intra-observer assessment of CA, KY, and LO with moderate reproducibility and variability in the inter and intra-observer assessment of AVR. This 3D stereoradiography technology enables reliable comparison of different surgical techniques for the treatment of AIS patients in future studies.

**Acknowledgements**

We thank biostatisticians Alexis Vistocky and Dr. Sergey Tarima from the Division of Biostatistics at Medical College of Wisconsin.

## O24 Ultrasound for quantitative assessment of spinal deformity in adolescent idiopathic scoliosis using EOS radiography as gold standard: A study of 952 chinese patients

### Yi-shun Wong^1^, Kelly Ka-lee Lai^2^, Yong-ping Zheng^2^, Lyn Lee-ning Wong^1^, Alec Lik-hang Hung^1^, Bobby Kin-wah Ng^1^, Benjamin Hon-kei Yip^3^, Yong Qiu^4,5^, Jack Chun-yiu Cheng^1, 5, 6^, Tsz-ping Lam^1, 5, 6^

#### ^1^Department of Orthopaedics and Traumatology, The Chinese University of Hong Kong, Hong Kong, China; ^2^ Department of Biomedical Engineering, The Hong Kong Polytechnic University, Hong Kong, China; ^3^ Division of Family Medicine and Primary Health Care, The Jockey Club School of Public Health and Primary Care, The Chinese University of Hong Kong, Hong Kong, China; ^4^ Spine Surgery, The Affiliated Drum Tower Hospital of Nanjing University Medical School, Nanjing, China; ^5^ Joint Scoliosis Research Center of the Chinese University of Hong Kong and Nanjing University, Hong Kong and Nanjing, China; ^6^ SH Ho Scoliosis Research Laboratory, Faculty of Medicine, The Chinese University of Hong Kong, Hong Kong, China

**Introduction**

Ultrasound has recently been developed for quantitative assessment of spinal deformity in adolescent idiopathic scoliosis (AIS). Although promising results were reported, various factors that correlate with its accuracy remain undefined.

**Objectives**

This diagnostic accuracy study aimed to evaluate the reliability and validity of ultrasound measurement for AIS patients of both genders with various curve severity, curve levels, body mass index (BMI), ages and heights using low radiation EOS radiography as the gold standard.

**Methods**

Subjects diagnosed with AIS and aged between 8 to 40 years were recruited. Standing posteroanterior EOS radiography and ultrasound imaging of the spine were done on the same day. Coronal Cobb angles (E_Cobb) were measured manually on EOS radiographs. Spinous process angles (SPA) were measured from volume projection imaging of the Scolioscan system with in-built automatic algorithm. Intra-class correlation coefficients (ICC) were used to examine reliability. Concurrent validity of ultrasound measurement was evaluated with linear regression analysis and cross-tabulation under different clinical parameters.

**Results**

952 AIS patients (75.7% female, age 16.7 ± 3.0 years) were studied. The intra-and inter-reliability were 0.988 and 0.949 for E_Cobb measurement; 0.916 and 0.838 for Scolioscan scanning respectively. Out of 1432 curves that were detected and matched by Scolioscan (E_Cobb 29.3 ± 11.8°, SPA 18.4 ± 8.5°), statistically significant correlation (r = 0.816, p < 0.001) was found between E_Cobb and SPA. Correlation were 0.629 for upper thoracic curves (UTC, defined as curves with apices at T6.5 or above); 0.873 for upper spinal curves (USC, apices between T7 to T12.5); and 0.740 for lower spinal curves (LSC, apices at L1 or below) (all with p < 0.001). Conversion formulae to predict E_Cobb from SPA were [predicted E_Cobb = P_Cobb = 7.39 + 1.26 x SPA] for USC and [P_Cobb = 10.08 + 0.96 x SPA] for LSC. For curves with E_Cobb below 30°, the absolute difference between P_Cobb and E_Cobb was ≤ 5° in 66.6% and 62.4% of USC and LSC; while P_Cobb underestimated E_Cobb by > 5° in 6.0% and 7.2% of USC and LSC. In addition, tall stature was associated with stronger correlation between E_Cobb and SPA. Similar degree of correlation was seen between males and females or between different quartiles of BMI.

**Conclusions and significance**

Scolioscan gives satisfactory accuracy of measurement for curves with (a) apices at T7 or lower, (b) E_Cobb < 30° and also for patients with taller stature. Ultrasound for quantitative assessment of spinal deformity in AIS can be considered in lieu of conventional radiographs, with results to be interpreted according to various degree of accuracy under different clinical conditions.

## O25 A novel approach to sagittal profiling of adolescent idiopathic scoliosis using 3D ultrasound

### Tin-Yan Lee^1^, Jason Pui Yin Cheung^2^, Wei Wei Jiang^1,3^, Connie Lok Kan Cheng^1^, Kelly Ka-Lee Lai^1^, Haris Begovic^1^, Dino Samartzis^2^, Michael Kai Tsun To^2^, Yong-Ping Zheng^1^

#### ^1^Department of Biomedical Engineering, The Hong Kong Polytechnic University, Hong Kong, China; ^2^ Department of Orthopaedics and Traumatology, The University of Hong Kong, Hong Kong, SAR, China; ^3^ College of Computer Science and Technology, Zhejiang University of Technology, Hangzhou, China

##### **Correspondence:** Yong-Ping Zheng (ypzheng@ieee.org)

**Introduction**

Sagittal profile differences between normal and scoliotic spine are needed to be investigated at the early stage to determine the possibility of the development of vertebrae rotation [1]. Though radiographic Cobb is the gold standard for assessing spinal sagittal curvature, X-ray is radioactive. Non-ionizing three-dimensional (3D) ultrasound had been demonstrated to be feasible in evaluating vertebrae features and coronal curvatures of the spine [2, 3, 4]. Yet no study has reported the reliability and accuracy of 3D ultrasound on sagittal curvature analysis. In this study, the reliability and validity of 3D ultrasound on assessing spinal sagittal curvature of patients with scoliosis were investigated.

**Methods**

Twenty-one AIS patients, both males and females (Age: 15.7±1.3 years; Cobb’s angle range 11.1 to 41.9°), underwent 3D ultrasound and EOS X-ray scanning of the spine. X-ray Cobb (XCA) was measured on sagittal X-ray image (Fig. 1a), while spinous processes and laminae of the vertebrae were identified from B-mode images. Sagittal images were then generated to measure the thoracic and lumbar ultrasound spinous process angle (USSPA) (Fig. 1b) and ultrasound laminae angle (USLA) respectively (Fig. 1c). The reliability (intraclass correlation coefficients (ICC) for the intra- and inter-observer variability) and validity (linear regression analysis and Bland-Altman method, with mean absolute difference (MAD)) were tested for two ultrasound angles as compared to the X-ray Cobb (XCA).

**Results**

The ICC showed very reliable measurements of both ultrasound methods (ICC≥0.941). Moderate and significant linear correlations were seen between the ultrasound methods and XCA (Thoracic (R^2^≥0.574) / Lumbar (R^2^≥0.635)) (Fig. 2) and the Bland-Altman plot showed a good agreement between both ultrasound angles and XCA (Fig. 3). The MADs of both ultrasound angles, corrected by the linear regression equations, and XCA showed no significant difference (MAD: USSPA 6.4±4.8°/6.1±4.4° and USLA 7.5±4.9°/5.3±4.2°; p≥0.326 for thoracic / lumbar respectively).

**Conclusion**

Good correlations were found between 3D ultrasound and Cobb angle, without differences in reliability and validity using spinous processes and laminae. Other than coronal deformity, sagittal deformity of spine of AIS patient can also be assessed by non-ionizing and relatively inexpensive ultrasound imaging.

**Trial Registration**

N/A

**Consent to publish**

Signed informed consents were obtained from all subjects and of the guardians of patients aged below 18 prior to the start of the study. All patients (or parents of the subjects for those under 18 years of age) provided informed consent

**References**

1. Schlösser TP, Shah SA, Reichard SJ, Rogers K, Vincken KL, Castelein RM. Differences in early sagittal plane alignment between thoracic and lumbar adolescent idiopathic scoliosis. Spine J 2014;14(2):282-290.

2. Chin KJ, Karmakar MK, Peng P. Ultrasonography of the adult thoracic and lumbar spine for central neuraxial blockade. Anesthesiology 2011;114(6):1459-1485.

3. Zheng YP, Lee TT, Lai KK, Yip BH, Zhou GQ, Jiang WW, Cheung JC, Wong MS, Ng BK, Cheng JC, Lam TP. A reliability and validity study for Scolioscan: a radiation-free scoliosis assessment system using 3D ultrasound imaging. Scoliosis Spinal Disord. 2016;11:13.

4. Brink RC, Wijdicks SPJ, Tromp IN, Schlösser TPC, Kruyt MC, Beek FJA, Castelein RM. A reliability and validity study for different coronal angles using ultrasound imaging in adolescent idiopathic scoliosis. Spine J 2017.

**Fig. 1 (abstract O25).**
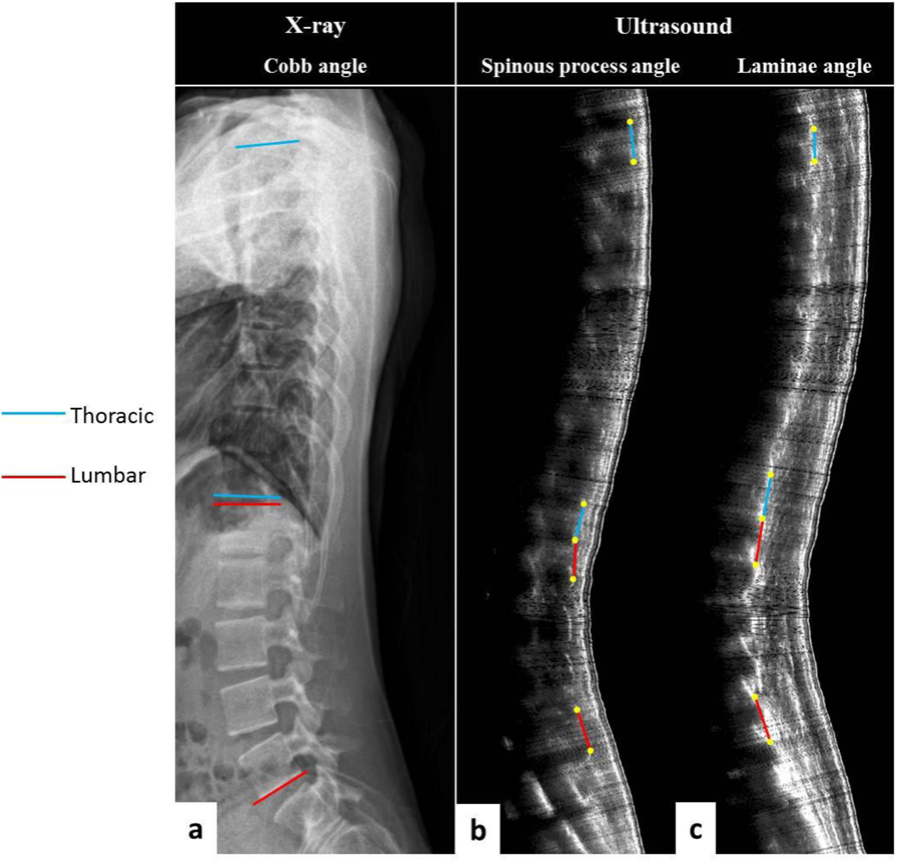
**a**) Measurement of X-ray Cobb’s angle (XCA); **b**) Measurement of ultrasound spinous process angle (USSPA); **c**) Measurement of ultrasound laminae angle (USLA) (Thoracic and lumbar angles are defined by the angle between the blue and red lines respectively)

**Fig. 2 (abstract O25).**
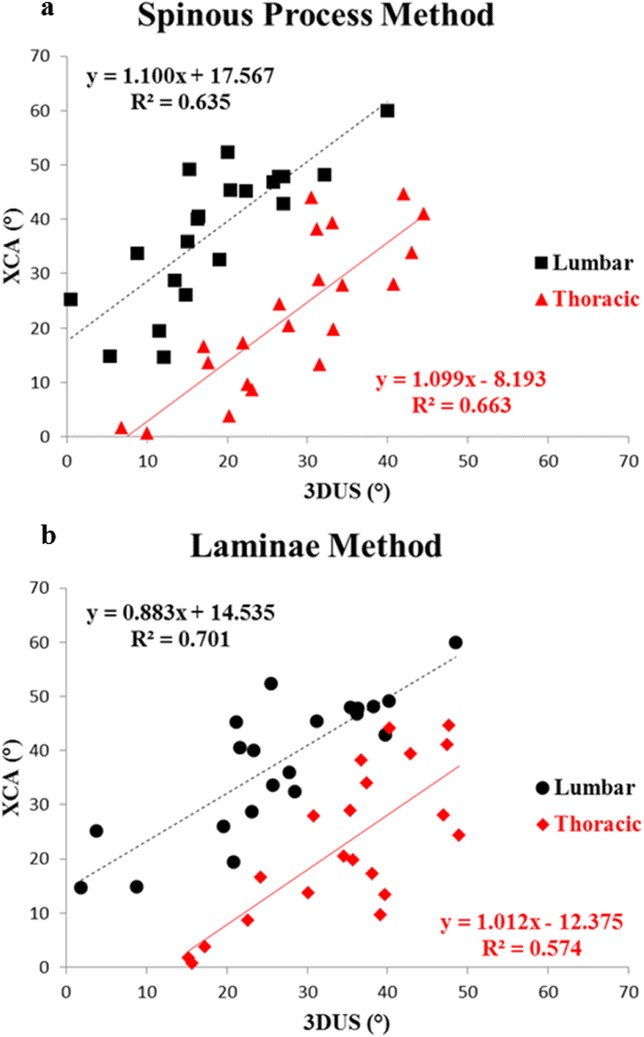
**a**) Correlations (**R**^**2**^) and equations between the X-ray Cobb’s angles (XCA) and the ultrasound spinous process angles (USSPA) for the thoracic and lumbar region; **b**) Correlations (**R**^**2**^) and equations between the X-ray Cobb’s angles (XCA) and the ultrasound laminae angles (USLA) for the thoracic and lumbar region

**Fig. 3 (abstract O25).**
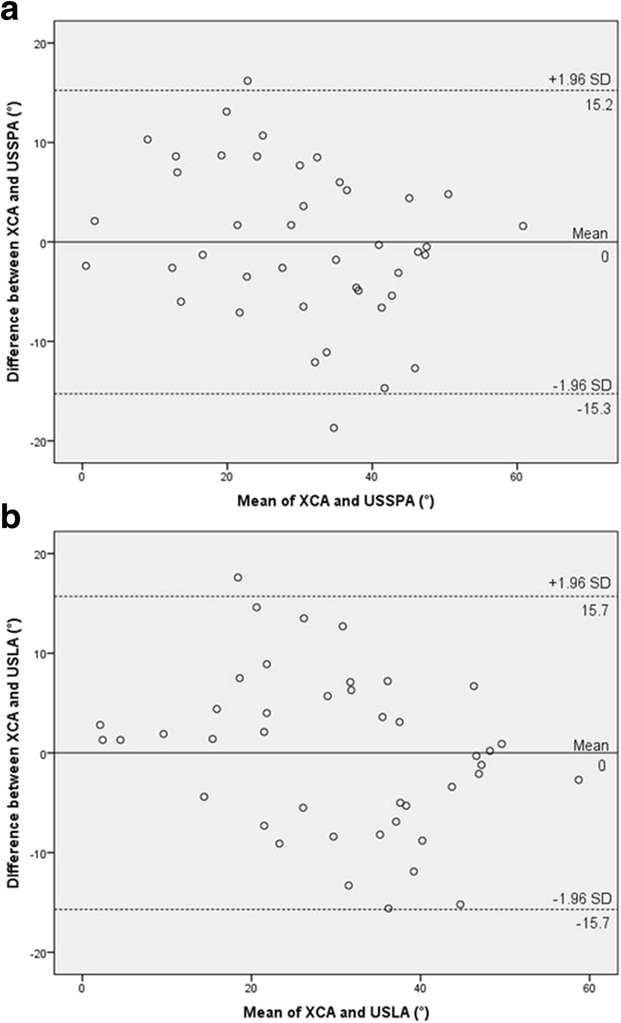
Bland-Altman plots that shows the differences between X-ray Cobb’s angles (XCA) and the sagittal ultrasound angles corrected with the linear regression equations. **a** Ultrasound spinous process angle (USSPA) and **b** ultrasound laminae angle (USLA). SD: standard deviation

## O26 Is cortical function associated with postural control altered in adolescents with idiopathic scoliosis?

### Julie Lanthier^1,2^, Sarah Lippé^2,3^, Inga S Knoth^2^, Catherine Bluteau^4^, Martin Simoneau^4,5^, Carole Fortin^1,2^

#### ^1^École de réadaptation, Faculté de médecine, Université de Montréal, Montreal, Quebec H3C 3J7, Canada; ^2^Centre de recherche, CHU Sainte-Justine, Montreal, Quebec H1T 1C5, Canada; ^3^Département de psychologie, Université de Montréal, Montreal, Quebec H3C 3J7, Canada; ^4^Département de kinésiologie, Faculté de médecine, Université Laval, Quebec, Quebec G1V 0A6, Canada; 5Centre interdisciplinaire de recherche en réadaptation et intégration sociale (CIRRIS), Quebec, Quebec, G1M 2S8 Canada

##### **Correspondence:** Carole Fortin (carole.fortin@umontreal.ca)

**Introduction**

Adolescent idiopathic scoliosis (AIS) is a multifactorial condition with unclear aetiology. Neurological impairments related to sensorimotor information processing have been suggested to play an important role in scoliosis mechanisms [1-3]. Previous studies have shown poorer postural performance and difficulty in reweighting sensory inputs following sensory deprivation in AIS group compared to age-matched controls [4,5]. Electroencephalography (EEG) has been used to document cortical activity associated with postural control. This study uses EEG frequency domain analysis to document cortical activity associated with postural control in adolescents with AIS compared to healthy control adolescents (CTRL).

**Objective**

To compare cortical activity associated with sensorimotor information processing related to postural control in open and closed-eyes conditions in adolescents with AIS and in CTRL.

**Methods**

Thirteen girls with a 15 to 45° AIS (13±1 years, 163±7 cm, 51±9 kg) and 14 age-matched CTRL girls (13±1 years, 158±11 cm, 50±9 kg) were recruited on two sites. EEG data was acquired using Electrical Geodesic systems. Participants stood in an upright standing posture with eyes open looking at an immobile fixation cross, approximately 2 m in front of the participant, for two minutes followed by two minutes with eyes closed. Fast Fourier Transformations (FFT) were performed to measure the power of the different frequency bands in both conditions. Cortical regions of interest (ROI) were central (electrodes C3, C4), frontal (F3, F4), parietal (P3, P4), temporal (T3, T4) and occipital (O1, O2). Significant differences were computed with p<0.05.

**Results**

During the “eyes closed” condition, differences between the AIS group and the CTRL group were found in beta and gamma frequency bands. The average power of beta signal was significantly larger in AIS group at the frontal, parietal, temporal and occipital ROI (Fig. 1). Also, the average power of gamma frequency was significantly larger in AIS group at the central, frontal, parietal and temporal ROI (Fig. 2).

**Conclusions and significance**

Our findings show enhanced beta and gamma frequency activity in adolescents with AIS compared to CTRL when holding a standing position while eyes closed, suggesting that more attention and vigilance are required in adolescents with AIS to maintain balance [6]. This may reflect less efficient cortical sensorimotor information processing associated with postural control in AIS. Future studies will assess if asymmetrical power within these ROI and frequency bands could be related to spine deformation progression.

**References**

1. Simoneau M, Lamothe V, Hutin É, Mercier P, Teasdale N, Blouin J. Evidence for cognitive vestibular integration impairment in idiopathic scoliosis patients. BMC Neurosci. 2009; 10:102.

2. Domenech J, Garcia-Marti G, Marti-Bonmati L, Barrios C, Tormos J.M, Pascuale-Leone A. Abnormal activation of the motor cortical network in idiopathic scoliosis demonstrated by functional MRI. Eur Spine J. 2011; 20 (7) :1069-78.

3. Pialasse J.P, Descarreaux M, Mercier P, Simoneau M. Sensory reweighting is altered in adolescent patients with scoliosis: Evidence from a neuromechanical model. Gait Posture. 2015; 42:558-63.

4. Haumont T, Gauchard GC, Lascombes P, Perrin PP. Postural instability in early-stage idiopathic scoliosis in adolescent girls. Spine. 2011; 36:E847-54.

5. Simoneau M, Mercier P, Blouin J, Allard P, Teasdale N. Altered sensory-weighting mechanisms is observed in adolescents with idiopathic scoliosis. BMC Neurosci. 2006; 7:68.

6. Pritchard TC, Alloway KD. Sommeil, coma et mort cérébrale : États de conscience et EEG. In : Neurosciences médicales: Les bases neuroanatomiques et neurophysiologiques. Traduction de la 1^ère^ édition. Paris : De Boeck Supérieur; 2002. p. 467-468.

**Fig. 1 (abstract O26).**
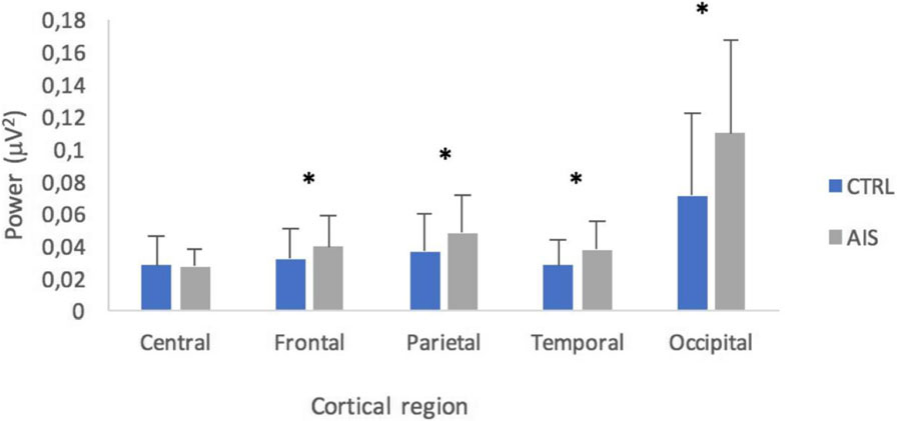
Average power (μV^2^) of beta frequency band at each cortical region of interest for AIS group and control group during “eyes closed” condition

**Fig. 2 (abstract O26).**
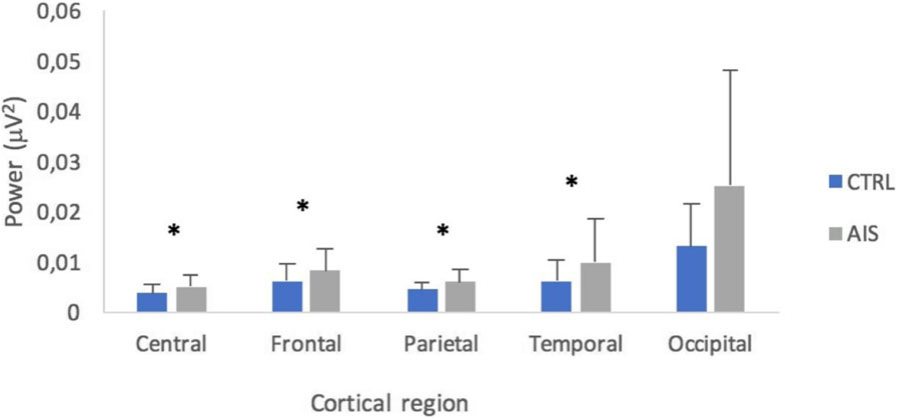
Average power (^μV2^) of gamma frequency band at each cortical region of interest for AIS group and control group during “eyes closed” condition

## O27 Axial rotation pattern may define the sagittal alignment in adolescent idiopathic scoliosis patients: A case-control study

### Hongda Bao, Shibin Shu, Qi Gu, Yuancheng Zhang, Zezhang Zhu, Zhen Liu, Yong Qiu

#### Nanjing Drum Tower Hospital, Nanjing, 210008, China

**Introduction**

A recent study based on 3D analysis revealed that increasing severity of coronal curvature is associated with a progressive loss of 3D thoracic kyphosis in AIS. However, the vertebral rotation is neglected in previous reports.

**Objective**

The aim of this study is to investigate the relationship between axial rotation pattern and sagittal alignment in AIS pts.

**Methods**

This study prospectively enrolled a series of Lenke type 1A female AIS pts. Hypo-kyphotic pts (TK<10°, DTK) and pts with normal TK (TK>20°, NTK) were matched by major Cobb angle, apex, Risser grade and Lenke classification. The biplanar full-spine images were taken before and 3 weeks after surgery, the curvature was reconstructed with dedicated software. Axial rotation parameters were obtained from reconstruction, including mean vertebral rotation of the major thoracic curve (MTR), mean vertebral rotation of the proximal thoracic curve (PTR) and mean vertebral rotation of the lumbar curve (LR). The paired t test was performed between the normal TK group and the hypokyphotic group.

**Results**

A total of 24 patients (12 pairs) were included (mean age 13.7 y/o) with baseline Cobb angle of 52.4 °. The mean pre-op TK of NTK groups was 28.2 °, while in DTK group was 11.2 °. There was a significant difference in the average vertebral rotation of the major thoracic curve between the two groups. The MTR of NTK group was significantly smaller than that of DTK group (10.2 ° vs. 12.7 °, p <0.001), and there was a significant correlation between TK and MTR (r = 0.30, p = 0.03). As for the lumbar curve rotation, the LR of the NTK group was significantly smaller than the DTK group (p=0.002), but the absolute value of the two groups was similar (p = 0.31). The lumbar Cobb angle correction rate was significantly greater in patients with thoracic and lumbar curve rotating in the same direction than that in the opposite direction (81.1% vs 61.9%, p = 0.005).

**Conclusion**

After matching the coronal deformity, the MTR of the hypokyphotic pts was significantly larger. The direction of the thoracic and lumbar curve rotation was also required to be well concerned in the preoperative surgical planning.

**Significance**

The results would benefit surgeons for the surgical decision-making in AIS patients with different baseline TK.

## O28 Patterns of intervertebral axial rotations in children with idiopathic scoliosis

### Xue-Cheng Liu^1,2,4^, John Thometz^1,2,4^, Channing Tassone^1,2,4^, Adam Thiessen^1,4^, Mahua Dasgupta^3,4^ Pippa Simpson^3,4^ , Aria Bagheri^4^

#### ^1^Deparment of Orthopedic Surgery, Medical College of Wisconsin, Milwaukee, WI 53206, USA; ^2^Musculoskeletal Functional Assessment Center, Children’s Hospital of Wisconsin, Milwaukee, WI 53226, USA; ^3^Quantitative Health Sciences, Children’s Hospital of Wisconsin, Milwaukee, WI 53226, USA; ^4^Medical College of Wisconsin, Milwaukee, WI 53206, USA

##### **Correspondence:** Xue-Cheng Liu

**Introduction**

Since the EOS imaging system (Biospace Med, Paris, France) has been applied in the measurement of scoliosis during full weight-bearing standing, it has provided with high-quality of 3D spinal modeling. Although the association between pelvic asymmetry, vertebral wedging and adolescent idiopathic scoliosis progression was reported using this new technology, different patterns of intervertebral axial rotation (IAR) across the spines of children with idiopathic scoliosis (IS) in Lenke type 1and its relation to curvature magnitudes are yet to be addressed.

**Objectives**

The purposes of the study were two folds: 1) to evaluate correlations between Cobb angles and IAR for Lenke type 1; 2) to analyze the patterns of the IAR for Lenke type 1.

**Methods**

Bilateral x-ray images of 53 children with IS (mean age 12.1 year-old) were retrospectively selected. The averaged Cobb angles was 30.3° (18°-56°). There were Lenke Type 1A (22), 1B (25) and 1C (6). Their EOS images were uploaded into the sterEOS computer program. With the use of software, spinal parameters were identified manually to construct a 3D model. It calculates the Cobb angles, lordosis, kyphosis, and the IAR of the each vertebra. We fit a third-Degree polynomial simultaneously to Lenke types 1A, B, C.

**Results and discussion**

Analysis of patients with Lenke type 1 showed the Cobb angle of the proximal curve was significantly correlated with the IARs of the apex, T1, T5-T8 (-0.39 – 0.30) (P<0.05), while the distal curve Cobb angle and the IARs of T4-T5, L2-L3 were correlated (-0.37 – 0.40) (P<0.05). The kyphosis correlated with the IARs at T11 (r=0.37, p= 0.006). No correlations were found between lordosis and the IARs of any vertebra.

The model predicted overall “S” patterns of IARs for the collective cohort from the T1 to L5 vertebral levels. The IARs involved in changes of orientation from +15 to -20 for the proximal curves and opponent direction from -5 to +25 for the distal curves. For type 1A we found that there was a barely observable minimum at Apex 1(A1) and similarly for the maximum at apex 2 (A2) with the largest scatter around the two apices. However, for both types 1B and 1C the minimum and maximum were much more marked occurring at the 6^th^ vertebra above A1 and the 4^th^ vertebra below A2. Further there was a significant difference of types 1B and 1C from type 1A (P< 0.02), but none between types 1B and 1C (P > 0.48).

**Conclusion and significance**

The EOS imaging system provides us a unique tool to reconstruct 3D spinal models and predicts two opposite orientations of intervertebral axial rotation. These orientations can be used to indicate how idiopathic scoliosis naturally intends to develop with respect to the balance of spinal segments, and the stability around the middle line of the trunk. This study will provide additional clinical input into the Lenke classification modifier, pre-surgical planning, and brace design for children with IS.

## O29 Toward a better understanding of sensorimotor function in adolescent idiopathic scoliosis

### Jean-Philippe Pialasse^1,2^, Martin Simoneau^3,4^, Inga S Knoth^2,5^, Sarah Lippé^2,5^, Carole Fortin^1,2^

#### ^1^École de réadaptation, Faculté de médecine, Université de Montréal, Montreal, Quebec H3C 3J7, Canada; ^2^Centre de recherche, Centre de réadaptation Marie Enfant, CHU Sainte-Justine, Montreal, Quebec H1T 1C9, Canada; ^3^Département de kinésiologie, Faculté de médecine, Université Laval, Quebec, Quebec G1V 0A6, Canada; ^4^Centre interdisciplinaire de recherche en réadaptation et intégration sociale (CIRRIS), Quebec, Quebec G1M 2S8, Canada; ^5^Département de psychologie, Université de Montréal, Montreal, Quebec H3C 3J7, Canada

##### **Correspondence:** Carole Fortin (carole.fortin@umontreal.ca)

**Introduction**

Although the etiopathogenesis of adolescent idiopathic scoliosis (AIS) remains unclear, among various hypotheses, alterations in sensorimotor processing could be involved in AIS onset. This suggestion has been supported by studies demonstrating altered sensory cortical processing and abnormal cortical network connectivity in AIS compared to controls[1-2]. Our group has detected poorer balance control and difficulty in reweighting sensory inputs suggesting a dysfunction of the central mechanisms performing sensorimotor processing in AIS[3-5]. Recently, electroencephalography (EEG) has been used to study cortical sensorimotor information processing associated with postural control[6]. This study uses alpha band event-related spectral perturbation (ERSP) to assess cortical activation patterns associated with postural control in adolescents with AIS and in healthy control adolescents (CTR).

**Objective**

To measure cortical activation patterns associated with sensorimotor information processing related to postural control during reintegration of ankle proprioception in adolescents with AIS compared to CTR using EEG.

**Methods**

Twelve girls with a 15 to 45° AIS (13±1 years, 162±7 cm, 51±10 kg) and 11 age-matched CTR girls (13±1 years, 160±4 cm, 51±8 kg) were recruited on two sites. Participants stand quietly on a force platform. They underwent 50 trials alternating open and closed-eyes conditions lasting 30 s each in three equal length epochs: 1) quiet standing, 2) sensory manipulation: ankle proprioception altered by 80Hz ankle tendon vibration and 3) sensory reintegration (cessation of vibration). EEG data were recorded using Electrical Geodesic systems. Data were processed using Matlab and EEGLAB. After EEG signal preprocessing, ERSP was calculated 1s before and 3s after the end of the vibration to single out brain activation during sensory transition. Electrodes of interest were C3, C4. Significant differences were computed with p<0.05.

**Results**

Regarding C3 (Fig. 1), during the closed-eyes condition, the AIS group had significantly more upper alpha band (frequency 10–13 Hz) desynchronization between 150 and 300 ms after cessation of vibration. During the open-eyes condition, this occurred for a longer time between 150 and 720ms. At C4 (Fig. 2), a significant higher desynchronization for AIS group was only found during the open-eyes condition: between 300 and 700ms and for few occurrences up to 3 seconds after cessation of vibration. There is also an interaction between group and visual condition on lower and upper alpha bands at both C3 and C4.

**Conclusions and significance**

Our results reflect a greater neural activity in the AIS group during sensory reintegration. In agreement with previous studies, difficulty to maintain balance control during sensory reintegration in patients with AIS suggests a link between scoliosis and sensorimotor processing impairment.

Four top left graphs represent frequency/time plot. The color gradient corresponds to green baseline calculated on the mean of the second before end of stimulation [-1 0], to red for more activity in this frequency, and blue to lower activity in this frequency. The five other graphs show in brown significant difference with p<0.05. Vertical lines on zero represent the end of vibration, horizontal lines help to delimit the alpha band. Condition with closed eyes is named “NonVis”, with open eyes “Vis”.

Four top left graphs represent frequency/time plot. The color gradient corresponds to green baseline calculated on the mean of the second before end of stimulation [-1 0], to red for more activity in this frequency, and blue to lower activity in this frequency. The five other graphs show in brown significant difference with p<0.05. Vertical lines on zero represent the end of vibration, horizontal lines help to delimit the alpha band. Condition with closed eyes is named “NonVis”, with open eyes “Vis”.

**References**

1. Wang,D, Shi L, Shangping L, et al. *Altered topological organization of cortical network in adolescent girls with idiopathic scoliosis.* PLoS One. 2013; 8(12): e83767.

2. Domenech J, Garcia-Marti G, Marti-Bonmati L, Barrios C, Tormos JM, Pascuale-Leone A. *Abnormal activation of the motor cortical network in idiopathic scoliosis demonstrated by functional MRI.* Eur Spine J. 2011; 20(7): 1069-78.

3. Simoneau M, Mercier P, Blouin J, Allard P, Teasdale N. *Altered sensory-weighting mechanisms is observed in adolescents with idiopathic scoliosis.* BMC Neurosci. 2006; 7: 68.

4. Simoneau M, Richer N, Mercier P, Allard P, Teasdale N. *Sensory deprivation and balance control in idiopathic scoliosis adolescent.* Exp Brain Res. 2006; 170(4): 576-82.

5. Pialasse JP, Descarreaux M, Mercier P, Blouin J, Simoneau M. *The Vestibular-Evoked Postural Response of Adolescents with Idiopathic Scoliosis Is Altered.* PLoS One,.2015; 10(11): e0143124.

6. Del Percio C, Brancucci A, Bergami F, et al. *Cortical alpha rhythms are correlated with body sway during quiet open-eyes standing in athletes: a high-resolution EEG study.* Neuroimage. 2007; 36(3): 822-9.

**Fig. 1 (abstract O29).**
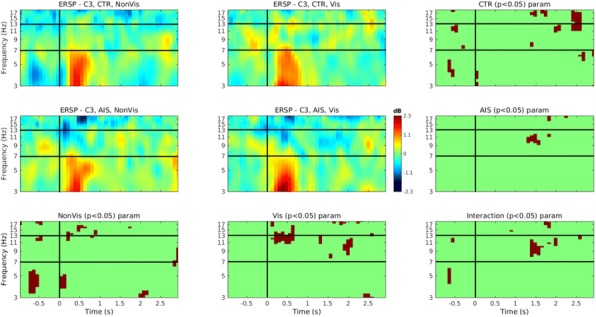
Event-related spectral perturbation (ERSP) over the left sensorimotor cortex C3

**Fig. 2 (abstract O29).**
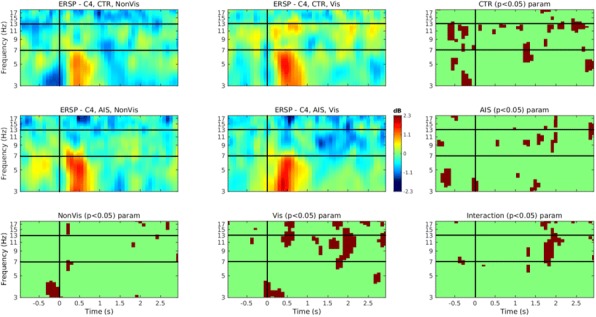
Event-related spectral perturbation (ERSP) over the right sensorimotor cortex C4

## O31 Does direct vertebral body derotation using multiple-level pedicle screws improve axial vertebral rotation? A pre- and post-operative analysis using biplanar radiography

### Kenoma Anighoro^1,3^, Channing Tassone^1.2.3^, Adam Miller^3^, John Thometz^1,2,3^, Xue-Cheng Liu ^1,2,3^

#### ^1^Deparment of Orthopedic Surgery, Medical College of Wisconsin, Milwaukee, WI 53206, USA; ^2^Musculoskeletal Functional Assessment Center, Children’s Hospital of Wisconsin, Milwaukee, WI 53226, USA; ^3^Medical College of Wisconsin, Milwaukee, WI 53206, USA

##### **Correspondence:** Kenoma Anighoro; Xue-Cheng Liu

**Introduction**

It has been previously established that sterEOS® (Biospace Med, Paris, France), a proprietary biplanar imaging system, allows reliable assessment of curve characteristics in adolescent idiopathic scoliosis (AIS) in three relevant planes (coronal, sagittal, and axial) under physiologic (i.e. standing) conditions with low radiation exposure. Using this technology, we demonstrate its value in measuring the effects in these three planes after treatment with direct vertebral body derotation (DVBD) using multiple-level pedicle screw instrumentation (MLPS).

**Objectives**

Our goals were to 1) determine changes in the coronal and sagittal plane; and 2) investigate changes of apical vertebral axial rotation following MLPS instrumentation through the use of SterEOS tools.

**Methods**

One observer retrospectively analyzed orthogonal radiographs obtained by simultaneous capture in two planes using sterEOS imaging system. Fifty-two patients with AIS who underwent corrective surgery between 2014 and 2016 at a single institution were included. Coupled radiographs were used to generate representative 3D models of the spine from T1 through L5 pre- and post-operatively. From these models, we determined the major curve coronal Cobb angle (CA), major curve apical axial vertebral rotation (AAVR), T4/T12 thoracic kyphosis (TK), and L1/L5 lumbar lordosis (LL). These data were then analyzed using paired t-testing and analysis of covariance.

**Results and discussion**

The average age of the included patients was 14.7 ± 2.2 (range 11 to 19 years) at time of surgery. The major curve was fully instrumented in all 52 patients. The average pre-operative CA was 58.0° ± 12.0° (range 33.0° to 90.3°). Post-operative CA was 19.8° +/- 8.3° (0.5° to 53.2°). The average change was –23.9° (p < 0.05). The average pre-operative TK was 15.0° ± 7.6° (-17.7 to 51.1°). The average post-operative TK was 20.9° ± 9.95° (-4.7° to 45.3°). The average change in TK was +3.89° post-operatively (p < 0.05). The average pre-operative LL was 47.76° ± 10.83° (22.5° to 56.2°). Average post-operative LL was 46.9° ± 10.15° (26.5° to 56.2°). The average difference in LL was - 0.8 (p = 0.4) .The average absolute value of the pre-operative AAVR was 14.4° ± 7.6° (0.1° to 29.2°). The average post-operative AAVR was 10.1° ± 5.5° (0.2° to 24.0°). The average change in AAVR was – 5.9° degrees (p <0.05). The AAVR was either stable or improved in 43/52 patients (82.7%).

**Conclusion and significance**

Our results demonstrate the usefulness of SterEOS system for measuring the surgical treatment effect of DVBD specifically with regards to AAVR and further confirm that DVBD confers significant axial correction in addition to coronal and sagittal plane correction within the region of instrumentation. SterEOS could eventually be used for routine perioperative measurement of axial rotation in addition to coronal and sagittal parameters, allowing more complete assessment of deformity in daily clinical practice.

## O32 Relationship between vertebral fracture burden and sagittal balance assessed by structured light back surface topography

### Jerzy Narloch^1^, Wojciech Glinkowski^1,2,3^

#### ^1^Department of Orthopaedics and Traumatology of Locomotor System, Baby Jesus Clinical Hospital, Warsaw, 02-005, Poland; ^2^Center of Excellence “TeleOrto” for Telediagnostics and Treatment of Disorders and Injuries of the Locomotor System, Medical University of Warsaw, Warsaw, 02-097, Poland; ^3^Faculty of Health Sciences and Physical Education, Kazimierz Pulaski University of Technology and Humanities, Radom, 26-600, Poland

**Introduction**

Recently, increasing recognition of the clinical importance of the sagittal plane alignment of the spine is frequently reported in the literature. The physiological spinal sagittal balance should serve as a baseline for the evaluation of pathological conditions associated with abnormal angular parameter values namely: sacral slope (SS), pelvic tilt (PT), pelvic incidence (PI), lumbar lordosis (LL), thoracic kyphosis (TK). Patients with vertebral fractures often suffer from unbalanced sagittal alignment.

**Objective(s)**

We aimed to evaluate the relationship between markerless, structured light back surface topography measurement system for posture and radiological assessment in patients with vertebral fractures.

**Method(s)**

The study included 61 patients with vertebral fractures, 41 women, and 20 men. The average age of patients was 70 (+/-15) years. Patients were treated using 3-point corset - Jewett brace to prevent further vertebra deformity or collapse. Torso deformation measurements were made using a system for three-dimensional assessment of the patient's body surface. The system uses a measurement technique with structured light and a posture assessment module based on the use of asymmetry indicators and the measurement of the size of curvatures of the spine in the sagittal plane.

**Result(s) and discussions**

Vertebral fracture burden was significantly correlated with sacral slope (SS), pelvic tilt (PT), pelvic incidence (PI) (r=0.34, r=0.47, and r=0.44, respectively; p<0.05). Neither radiological lordosis nor kyphosis showed significant correlation with fracture burden. Topographic scapulae angle was highly associated with increasing number and severity of vertebral fractures (r=-0.061, p<0.05). Among demographic characteristics, age showed significant association with pelvic tilt (r=0.35, p<0.05).

Kruskal-Wallis test showed age differed significantly in patients with more vertebral fractures compared to younger subgroup (p=0.02). Increasing fracture burden significantly influenced the differences in pelvic incidence (p=0.01) and pelvic tilt (p=0.02). Radiological sagittal curvatures, patients’ physical characteristics, topography-derived curvatures, sagittal balance, frontal balance, pelvis angle, waist angle, shoulders angle, the angle of trunk rotation, and Suzuki Hump Sum did not differ significantly with increasing level of vertebral fracture burden.

**Conclusion(s) and significance**

The severity of sustained vertebral fractures is clearly reflected in pelvic parameters describing sagittal balance – probably developed as a compensatory measure. The system using the structured light back surface topography measurement technique allows collecting accurate and repeatable body measurements without using X-rays.

Acknowledgement

This study was supported by grant Nr-13-0109-10/2010 Funded by National Center for Research and Development

## O33 Mapping vertebral levels during ultrasound assessment of spinal deformities in girls with adolescent idiopathic scoliosis (AIS) – construction of a reference table for clinical use

### Isabel Tran^1^, Yi Shun Wong^1^, Lyn Lee Ning Wong^1^, Yongping Zheng^2^, Ka Lee Lai^2^, Wayne YW Lee^1^, Bobby Kin Wah Ng^1^, Alec Lik Hang Hung^1^, Jack Chun Yiu Cheng^1^, Tsz Ping Lam^1^

#### ^1^ SH Ho Scoliosis Research Laboratory, Joint Scoliosis Research Center of the Chinese University of Hong Kong and Nanjing University, Department of Orthopaedics and Traumatology, The Chinese University of Hong Kong, Prince of Wales Hospital, Shatin, Hong Kong SAR, China; ^2^ Department of Biomedical Engineering, The Hong Kong Polytechnic University, Hong Kong SAR, China

**Introduction**

Ultrasound has been reported to be useful for measuring Cobb angle in adolescent idiopathic scoliosis (AIS). It can also measure the distance from T1 to ends of a curve or its apex, but cannot identify the vertebral level due to lack of a reference table.

**Objective(s)**

To develop a reference table on linear dimension along the vertebral column for each vertebra and intervertebral disc so that the level of the end-vertebra and apical vertebra can be determined.

**Method(s)**

This was a cross-sectional study on 293 female AIS patients aged 8-40 years old with Cobb angle between 10-40° (mean:24.3°±7.8°). EOS frontal radiography of the whole spine was taken. Centroid of the vertebral body was defined as the point of intersection between the diagonal lines drawn over the vertebral body. The mid-point of the intervertebral disc was also determined. To control confounding from spinal height, the “Fractional Distance”, FDi for the ith vertebra, was used and defined as ([Cumulative distance from the centroid of T1 to that of the ith vertebra]/[Cumulative distance from centroid of T1 to that of L5])x100%. The “Fractional Distance”, FD[i-(i+1)] for the intervertebral disc between ith and (i+1)th vertebra, was calculated similarly as ([Cumulative distance from centroid of T1 to the midpoint of that intervertebral disc]/[Cumulative distance from centroid of T1 to that of L5])x100%.

**Result(s) and discussions**

Intra-observer reliability of measurement showed an intraclass correlation coefficient of 0.963. 99% confidence intervals(CI) for FDi in % were tabulated as exemplified by T2:(4.41-4.50), up to L4:(92.50-92.69). Corresponding measurements for intervertebral discs were similarly tabulated as exemplified by T1-T2:(2.07-2.15) up to L4-L5:(96.07-96.21). Notably, these 99% CI on Fractional Distance for adjacent vertebrae and intervertebral discs did not overlap for all measurements.

**Conclusion(s) and significance**

A reference table on linear dimension along the vertebral column for female AIS subjects is constructed. The fact that 99% confidence intervals for adjacent vertebrae and intervertebral discs do not overlap supports the feasibility of using the reference table with which ultrasound evaluation of scoliosis can be conducted not only to determine the Cobb angle but also the levels of the end and apical vertebrae. Further studies are warranted to validate the level mapping with a new cohort of patients and to develop similar reference tables for male patients.

With availability of the reference table, ultrasound evaluation of scoliosis can include Cobb angle, the levels of the end-vertebra and the apex, thus enabling a comprehensive frontal plane evaluation with radiation-free ultrasound imaging.

## O34 Using ultrasound imaging method to determine spinal flexibility for children with AIS under observation or non-surgical treatment

### Mahdieh Khodaei^1^, Doug Hill^2^, Rui Zheng^2^, Lawrence H Le^1^, Edmond Lou^1,3^

#### ^1^Department of Radiology and Diagnostic Imaging, University of Alberta, Edmonton, Canada; ^2^Department of Surgery, University of Alberta, Edmonton, Canada; ^3^Department of Electrical and Computer Engineering, University of Alberta, Edmonton, Canada

##### **Correspondence:** Mahdieh Khodaei; Edmond Lou

**Introduction**

Children who have mild or moderate scoliosis may put under observation or non-surgical treatment regimen. Spinal flexibility is one of the parameters which may help clinicians to provide better treatment planning. The traditional supine side-bending radiograph method may not be used for these 2 groups due to extra ionizing radiation. Ultrasound has been introduced to measure proxy Cobb angle and vertebral rotation reliably. Three flexibility indices which could differentiate the gravitational and structural components of the curve were developed and applied in this study.

**Objective**

To use ultrasound (US) imaging method to determine if spinal flexibility was different between the observation and non-surgical treatment groups.

**Method**

One-hundred-eight (93 F; 15 M) AIS subjects, aged 13.7±1.7 years old were consented and recruited. There were 43 subjects under observation and 65 subjects under treatment (48 bracing; 3 exercise, and 14 exercise and bracing). All subjects were scanned by US machine in standing, normal prone, and maximum prone left or right or both sides-bending. One experienced rater measured the lateral curvatures and the maximum axial vertebral rotation (AVR) on standing only. Three flexibility indices as bending-relative-to-standing-index (BRSI), prone-relative-to-standing index (PRSI) and bending-subtract-prone-relative-to-standing index (B-PRSI) were calculated as following:

$$ \mathrm{BRSI}=\frac{\mathrm{US}\ \mathrm{standing}\kern0.5em \mathrm{Cobb}-\kern0.5em \mathrm{US}\ \mathrm{bending}\ \mathrm{Cobb}\kern0.5em }{\mathrm{US}\ \mathrm{standing}\ \mathrm{Cobb}} $$

$$ \mathrm{PRSI}=\frac{\ \mathrm{US}\ \mathrm{standing}\ \mathrm{Cobb}-\mathrm{US}\ \mathrm{prone}\ \mathrm{Cobb}\kern0.5em }{\ \mathrm{US}\ \mathrm{standing}\ \mathrm{Cobb}} $$

$$ \mathrm{B}-\mathrm{PRSI}=\frac{\ \mathrm{US}\ \mathrm{prone}\kern0.5em \mathrm{Cobb}-\mathrm{US}\ \mathrm{bending}\ \mathrm{Cobb}\kern0.5em }{\ \mathrm{US}\ \mathrm{standing}\ \mathrm{Cobb}} $$

A comparison between the observation and non-surgical treatment groups of the standing Cobb angle, AVR and 3 indices were reported.

**Results and discussions**

For the observation and non-surgical treatment groups, there were 70 and 103 curves identified, respectively. The Cobb angle and AVR between the observation versus the non-surgical treatment groups were (27.3±14.0, 9.1±5.5) versus (25.1±9.0, 8.0±3.8), respectively. The ranges of the Cobb angle and AVR of the observation group was larger than the treatment group. The 3 indices BRSI, PRSI and B-PRSI between two groups were 1.4±0.7, 0.5±0.2, 0.9±0.6 versus 1.3±0.6, 0.5±0.2, 0.8±0.5, respectively. Based on the 3 indices, there was no significant difference between two groups.

**Conclusion and significance**

There was no significant difference on the spinal flexibility between observation and non-surgical treatment groups. Since the observation group included subjects from the initial stage to the final stage, the bone age information may need to use to perform further analysis.

## O35 Does intraoperative imaging decrease pedicle screw-related complications in surgical treatment of adolescent idiopathic scoliosis: A systematic review update and meta-analysis

### Andrew Chan^1^, Eric Parent^2^, Jason Wong^3^, Karl Karvacan^4^, Cindy San^5^, Edmond Lou^6^

#### ^1^Department of Biomedical Engineering, University of Alberta, Edmonton, Alberta, T6G 2V2, Canada; ^2^Department of Physical Therapy, University of Alberta, Edmonton, Alberta, T6G2G4, Canada; ^3^Department of Electrical Engineering, University of Alberta, Edmonton, Alberta, T6G1H9, Canada; ^4^Faculty of Medicine and Dentistry, University of Alberta, Edmonton, Alberta, T6G2R7, Canada; ^5^Faculty of Pharmaceutical Sciences, University of British Columbia, Vancouver, British Columbia, V6T1Z3; ^6^Department of Electrical Engineering, University of Alberta, Edmonton, Alberta, T6G1H9, Canada

**Introduction**

Posterior spinal instrumentation and fusion with pedicle screws is the standard surgery for adolescent idiopathic scoliosis (AIS) curve correction. Free-hand methods and image guidance are two methods for screw insertion to prevent screw-related neurologic or vascular complications. However, due to the radiation and cumbersome nature of image guidance, it remains controversial to use in AIS surgery. A prior systematic review found moderate evidence that image guidance improved breach rates compared with free-hand methods and conflicting evidence of improvements in screw-related complication rates.

Objective: To update a prior systematic review and perform meta-analysis on complication and breach rates of posterior instrumentation and fusion in adolescent idiopathic scoliosis, comparing image guidance methods with free-hand methods.

**Methods**

The systematic review followed the methodology of the Preferred Reporting Items for Systematic Review and Meta-Analysis Protocols (PRISMA). A search strategy that focused on AIS patients undergoing posterior instrumentation and fusion with pedicle screws was developed and databases including MEDLINE, EMBASE, CINAHL, CENTRAL and Web of Science were searched. Articles were screened in separate abstract screening, full-text screening and extraction stages with two reviewers at each stage. Abstract inclusion criteria were AIS patients, posterior surgery with pedicle screws. Full-text inclusion criteria added reporting of free-hand versus image guidance methods for screw insertion, complications and breaches reporting. Risk of bias was assessed using the Quality in Prognosis Studies tool with two reviewers.

**Results and discussion**

The original systematic review had studies from 1970 to September 2015 with 79 studies fulfilling inclusion criteria. The systematic review update found 548 unique articles, 265 articles satisfying abstract screening criteria and 34 meeting full-text criteria. Fifteen articles have been extracted to date: three moderate risk of bias studies and 12 high risk of bias studies. From these articles, there is moderate evidence that breach rates are lower with image guidance (2 studies at 3.7%-6.9% vs 1 study at 12.1%) while screw-related complication rates were lower with image guidance (0% vs 1.4%). This compares to a prior review showing breaches at 3.7-7.9% vs 9.7-17.1% and complications at 0% vs 0-1.7%. Meta-analysis is planned when all articles have been extracted and will combined with the prior study.

**Conclusion**

The updated evidence on breach rates and complications are still limited by lack of comparison groups and poorly defined usage of free-hand and image guidance methods. There is moderate evidence of improvement in breach rates and screw-related complications when using image guidance compared to free-hand studies.

## O36 Accuracy of 3D ultrasound-camera guidance for posterior spinal surgery for adolescent idiopathic scoliosis

### Andrew Chan^1^, Eric Parent^2^, Edmond Lou^3^

#### ^1^Department of Biomedical Engineering, University of Alberta, Edmonton, Alberta, T6G 2V2, Canada; ^2^Department of Physical Therapy, University of Alberta, Edmonton, Alberta, T6G2G4, Canada; ^3^Department of Electrical Engineering, University of Alberta, Edmonton, Alberta, T6G1H9, Canada

**Introduction**

Severe adolescent idiopathic scoliosis is often treated surgically with posterior instrumentation and fusion with pedicle screws. Accuracy in placing pedicle screws can be improved using image guidance when compared with free-hand screw insertion methods. Current image guidance systems including fluoroscopy or intraoperative CT require usage of ionizing radiation and are cumbersome and bulky in the operating room, disrupting the flow of the operation. 3D intraoperative spinal ultrasound has been proposed as a potential method to assist insertion of pedicle screws.

**Objective**

To determine the accuracy of vertebra reconstruction and screw insertion trajectories on a vertebral phantom using a custom developed 3D spinal ultrasound-camera system.

Methods: Optitrack Prime 13W motion capture cameras were used for tracking an Ultrasonix 38mm linear transducer operating at 6.67 MHz with an Ultrasonix SonixTablet. Software was developed in Matlab to stream data from the two systems to generate 3D reconstructions of the vertebral surface. A custom transducer holder was 3D printed to attach motion capture markers. A T5 vertebra phantom was 3D printed and scanned five times to determine the accuracy of linear and angular dimensions on the reconstructed phantom. Measurements were made three times for each dimension measured. To determine the accuracy of screw insertion trajectory when using the 3D ultrasound navigator, the phantom included holes at the usual screw insertion point with trajectories between 0-30^o^. The left pedicle had straight down, medial and lateral trajectories while the right had down, superior and inferior trajectories to a total of six trajectories. A screw driver with four motion capture marker mounts was 3D printed and used to place screws into the holes on the vertebra five times. The entry-point and trajectory of the screwdriver recorded during placement and compared to the physically measured position and orientation of the screw in the capture volume.

**Results**

The accuracy of reconstructions was within 0.55±0.25mm for linear dimensions and 1.8±0.5^o^ for angular dimensions. The accuracy of reconstruction is strongly affected by the image processing filter parameters and the incident angle of ultrasound to create the reconstruction. This study used constant image processing filters to generate these reconstruction results. The average accuracy of the screw entry point was 0.27±0.14mm and the average accuracy of the screw trajectory was 0.72^o^±0.44^o^. Further study into vertebrae that are placed at different orientations and positions within the capture volume needs to be completed.

**Conclusion**

The custom developed ultrasound-camera system was able to reconstruct the vertebra accurately (0.55mm for linear and 1.8^o^ for angular) and had screw insertion accuracies of within 0.27mm and 0.72^o^ which are both within the desired accuracy of 1mm and 5^o^ to prevent pedicle breach.

## O37 Cervical extensor muscles atrophy in the patients with single level cervical spondylotic radiculopathy treated by fusion with posterior cervical cages placed bilaterally in the facet joints

### Paweł Główka^1,2^, Piotr Janusz^1^, Tomasz Kotwicki^1^, Kris Siemionow^2^

#### ^1^Department of Spine Disorders and Pediatric Orthopaedics, University of Medical Sciences, Poznan, Poland; ^2^Department of Orthopaedics, University of Illinois at Chicago, Chicago, IL, USA

**Introduction**

Spinal surgery involving open approaches inevitably causes muscle injury. Recent studies present the deep cervical extensor muscles atrophy after different spinal surgeries strategies: expansive open door-laminoplasty 68,9% [2], conventional open-door laminoplasty 60% [1] and 44% [3], skip laminectomy 13% [1] muscle preserving laminoplasty 12% [3]. The amount of muscles atrophy is observed to be related to the invasiveness of the exposure.

**Objectives**

The purpose of the study was to evaluate cervical extensor muscles atrophy after indirect root decompression with fusion with posterior cervical cages placed bilaterally in the facet joints.

**Methods**

The study includes 30 patients who undergone bilateral procedure of indirect root decompression with posterior cervical cages placed bilaterally in the facet joints. Patients were examined twice with CT: pre-surgery and at one year follow-up. The following cervical muscles cross sectional area (CSA) was measured on both pre- and post-surgery CT scans: multifidus, semispinalis cervicis, semispinalis capitis, splenicus. The pre-surgery and post-surgery lordosis was measured on standing cervical spine X-rays. The clinical outcomes were evaluated with Neck Disability Index (NDI), Visual Analogue Scale (VAS) for neck pain and VAS for arm pain.

**Results**

The differences between the pre-surgery versus the post-surgery muscles CSA (ΔCSA) were significant (CSA increased) for the following cervical muscles: multifidus, semispinalis cervicis, semispinalis capitis. There was no significant correlation between the ΔCSA versus the NDI improvement, the neck VAS and the arm VAS, respectively however, a positive correlation of the neck VAS improvement and the left splenicus ΔCSA was noted (r=0.52, p<0.05). Age of the patients presented the negative correlation with ΔCSA, but only the result for left multifidus was statistically significant (r=-0.41, p<0.05).

**Conclusions and significance**

This is the first study presenting evaluation of cervical extensor muscles atrophy after indirect root decompression with fusion with posterior cervical cages placed bilaterally in the facet joints. The most vulnerable to the surgery related injury according to our results is the cervical multifidus muscle and it is in consistence with the Fujimura and Nishi findings [1]. Our study presents the 13% of cervical multifidus muscle atrophy with only approximately 1% atrophy of the semispinalis cervicis and 1% atrophy of semispinalis capitis, and no atrophy but hypertrophy of the splenicus muscle. The fusion with posterior cervical cages placed bilaterally in the facet joints is the muscle preserving procedure with improvement in clinical outcomes in one-year follow up assessed with the NDI score, VAS for neck pain and VAS for arm pain.

1. Fujimura Y, Nishi Y (1996) Atrophy of the nuchal muscle and change in cervical curvature after expansive open-door laminoplasty. Archives of orthopaedic and trauma surgery 115(3-4):203–205

2. Bogduk N, Wilson A, Tynan W (1982) The human lumbar dorsal rami. Journal of Anatomy 134 (Pt 2):383

3. Fernandez-De-Las-Penas C, Albert-Sanchıs JC, Buil M, Benitez JC, Alburquerque-Sendın F (2008) Cross-sectional area of cervical multifidus muscle in females with chronic bilateral neck pain compared to controls. Journal of Orthopaedic & Sports Physical Therapy 38(4):175–180

## O38 Minimally invasive sacroiliac arthrodesis: 1 and 2-year results highlighting compression, grafting, and stabilization

### Scott A. Mitchell, William W. Cross III

#### Department of Orthopedic Surgery, Mayo Clinic, Rochester, MN, USA

**Introduction**

Sacroiliac (SI) joint pain remains an underdiagnosed and under-recognized source of chronic low back pain, especially in degenerative/deformative spine disease. As advances in minimally invasive approaches have become popularized, new techniques and technology are evolving. Studies in MIS SI arthrodesis remain limited due to small cohort sizes and industry-sponsored bias.

**Objective**

The purpose of this study was to report upon a single-surgeon series and outcomes of patients undergoing primary MIS sacroiliac arthrodesis utilizing a technique highlighted by SI joint compression, SI joint preparation for arthrodesis, and autografting at a major academic institution.

**Methods**

Between 2013 and 2016, 52 patients were available for review after undergoing primary, unilateral SI joint arthrodesis via percutaneous joint decortication, application of autograft/allograft, and placement of a modified lag screw by a single surgeon. Prospectively collected data, including a VAS, SF-12, ODI, and other standardized clinical questionnaires were collected at the pre-operative visit and the 3 month, 6 month, 12 month, etc. postoperative visits. CT scans were evaluated for evidence of radiographic fusion with bone bridging from the sacrum to the ilium.

**Results**

46 of the 52 patients (88%) had complete follow-up including patient outcome questionnaires and CT scans at the one year postoperative mark. In this cohort, 92% of patients gained CT-proven evidence of bony fusion at an average of 9 months postoperatively. 94% of the operated sacroiliac joints remained revision-free at an average of 2.4 years. Post-operatively, patients exhibited significantly improved ODI and SF-12 functional scores at all time points (ODI: pre-op 73.3; 3-month 58.6; 24-month 50.2; p < 0.01. SF-12: pre-op 31.4; 3-month 35.9; 24-month 37.1; p <0.01). Back and leg visual analog pain scores decreased post-operatively by 57% and 70%, respectively (p < 0.01). Median hospital stay was 1 day, median estimated blood loss was less than 50cc, and there were no recorded major complications.

**Discussion**

We report a large case series with standardized outcomes questionnaires and CT scans that demonstrate significant clinical improvement, decreased pain scores, and true arthrodesis in the vast majority of patients. This is a safe and effective technique for the appropriately diagnosed patient. This series highlights that excellent clinical outcomes and documented arthrodesis can be achieved by adhering to the 3 standard principles of arthrodesis: joint preparation, joint compression, and joint stability. It is clear that long-term studies of > 1-2 years are needed to assess the durability of SI fusion surgery.

**Significance**

This is the largest single-surgeon series that looks at third-generation sacroiliac arthrodesis techniques. Additionally, this series also represents the largest SI outcomes case series that is not sponsored by device manufacturing companies.

## O39 Surgical treatment of early-onset scoliosis by controlling asymmetrical growth in neurocentral synchondrosis: A preliminary study in an immature animal model

### Liang Xu

#### Spine Surgery, Drum Tower Hospital, Nanjing University Medical School, Nanjing, China

**Introduction**

Unilateral transpedicular screw fixation that passes through the neurocentral synchondrosis (NCS) can create scoliosis with the convexity on the side of the screw fixation. However, it remains unknown whether unilateral transpedicular screw fixation on the concave side could control the asymmetrical growth in NCS as well as halt the progression of scoliosis curve.

**Objective**

To evaluate the outcomes through implanting unilateral transpedicular screw fixation on the concave side in the porcine scoliosis model.

**Methods**

Twelve pigs were randomly assigned into two groups. In the control group, six animals received a sham operation without pedicle screw fixation. In the experimental group, six animals were treated with a single left (concave side) transpedicular screw placed across the NCS from T8 to T12. All animals were then observed for an 2-month period.

**Results**

There were no significant difference between two groups in the Cobb angle and vertebral rotation. After treatment with pedicle screws on the concave side, there were no significant progressions in the experimental group with regard to the Cobb angle (27.3±8.8° vs. 25.7±8.2°, P=0.137), vertebral rotation (19.1±7.1° vs. 17.6±8.4°, P=0.231) as well as the ratio of pedicle length between two sides (13.6±4.4% vs. 14.5±3.7%, P=0.311). Whereas in the control group, considerable progressions were found in the Cobb angle, vertebral rotation as well as the ratio of pedicle length between two sides (P<0.05 for all). Moreover, the Cobb angle and vertebral rotation in the control group was significantly higher than in the experimental group (P<0.05).

**Conclusions and significance**

Unilateral transpedicular screw fixation on the concave side can effectively control the asymmetrical growth in NCS as well as halt the progression of scoliosis curve.

## O40 A new cyclic implant based on vertebral tethering for the treatment of adolescent idiopathic scoliosis: A feasibility demonstration

### Viviane Lalande^1,2^, Isabelle Villemure^1,2^, Manuel Vonthron^1,2^, Stefan Parent^2,3^, Carl-Éric Aubin^1,2,3^

#### ^1^Mechanical Engineering, Polytechnique Montréal, Montréal, QC H3T1J4, Canada; ^2^CHU Sainte-Justine, Montréal, QC H3T1C5, Canada; ^3^Université de Montréal, Montréal, QC H3T1J4, Canada

**Introduction**

Anterior vertebral body tethering is a recent surgical technique aiming for the early correction of scoliotic deformities. It uses growth modulation resulting from unilateral compression with a tether, which induces a sustained pressure on the vertebral epiphyseal growth plates (GP). Experimental studies have demonstrated that cyclic compression would be less damaging than sustained compression to vertebral soft tissues. Hence, it would be of interest to develop a prototype of anterior vertebral body tethering using cyclic compression (i.e. with time dependent amplitude).

**Objectives**

The aim of this study was to experimentally demonstrate the feasibility of cyclic anterior vertebral body tethering using a porcine model.

**Methods**

Two healthy Duroc pigs (female; 48-50kg) were placed, under anesthesia, in lateral decubitus and a posterolateral thoracotomy was performed. A tether, spanning from T7 to T10, was fastened at the T7 level and could slide in all the remaining screw heads. It extended further down to be connected to an external motor, which cyclically pulled the tether. Between T10 and the external motor, a sheath was placed around the tether (Bowden-type tether) and allowed for the force transfer to compress the instrumented segment. Two force sensors measured the tether tension while a force sensor was slid between the disc and the vertebral end plate at the mid-instrumented level (T8-T9) to verify the prototype ability to transmit compression inside the spine. The motor pulled on the tether cyclically (0.1Hz) at ±30% for the following mean amplitudes: 29N, 35N, 40N, 44N, 49N. Each mean amplitude test was repeated three times. After this experiment, the pigs were euthanized without awakening.

**Results and discussion**

The implant was successfully implanted, and the motor was able to compress the spine by pulling on the tether. The applied tether tension had, on average, a difference of -2% ±3.2% (standard deviation) compared to the tension imposed in the prototype. The frequency and amplitude of the tether tension were synchronized with the compression measured in the spine. However, a mean lag of 0.5s was observed between responses; it may be explained by the time it takes to tension the tether.

**Conclusions and significance**

This preliminary experiment shows that the cyclic compression of the spine of an animal similar to the pediatric human is feasible. It thus opens the door to further in vivo experiments on a larger time scale to study the growth plate mechanobiological response to the implant. Since researchers have demonstrated that soft tissues were altered under a static compression, this research is a step towards protecting these soft tissues while correcting the deformity with tethering based on cyclic compression.

## O41 Comparative analysis of navigated and “free-hand” transpedicular screw placement techniques in surgical treatment of children with idiopathic scoliosis

### D. N. Kokushin, S. V. Vissarionov, S. M. Belyanchikov, V. V. Murashko, K. A. Kartavenko, N. O. Khusainov

#### Department of Spine pathology and Neurosurgery. Turner scientific research institute for pediatric orthopedics Ministry of health, Saint – Petersburg. Saint-Petersburg, 196603, Russia

##### **Correspondence:** N. O. Khusainov (nikitahusainov@gmail.com)

**Introduction**

Screw malposition during surgical treatment of adolescent idiopathic scoliosis remains an important issue due to potentially devastating effect of incorrect screw placement. CT - navigation technique is constantly developing method for improvement of treatment results.

Objective. To compare the correctness of transpedicular screw placement using the CT - navigated and “free-hand” techniques in surgical treatment of children with adolescent idiopathic scoliosis.

**Methods**

96 patients at the age of 14-18 years with idiopathic scoliosis Lenke type I and V were surgically treated and their results were evaluated. Study group (n=66) included patients who were operated using CT - navigation system for transpedicular screws placement. In the control group (n=30) “free-hand” technique was used. Correctness of screw position was evaluated with postoperative CT and a scale proposed by S.D. Gertzbein et al.

**Results**

Total amount of screws was 1166 in the study group and 546 in control group. In the study group the were 96% of screws (1119) placed correctly, and only 4% (47) incorrectly. In the control group correct placement was found to be in 78% (426 screws) of cases, incorrect position took place in 22% (120 screws) which was statistically significant (p<0,05). In the study group incorrect placement in the thoracic region was found in 4.8% of cases and in lumbar area in 2.5%. For the control group: 35.1% and 10.1% for the same areas respectively.

**Conclusion**

Navigation system is a useful tool which can significantly improve the results of surgical treatment of children with idiopathic scoliosis by increasing the amount of properly placed screws.

**Significance**

Specialized departments for spinal deformity correction should have a CT-navigation system and every spinal surgeon should be familiar with this technique as this significantly improves the final results of the surgery.

Keywords: idiopathic scoliosis, transpedicular systems, navigation, children.

## O42 Histomorphometric study of growth modulation of porcine vertebrae instrumented with a double epiphyseal implant

### Alejandra Mejía Jaramillo, Carl-Eric Aubin, Irène London, Bahe Hachem, Stefan Parent, Isabelle Villemure

#### École Polytechnique de Montréal, Institute of Biomedical Engineering, Montréal, Canada

**Introduction**

New fusionless approaches are currently developed for the early treatment of scoliosis. With the objective of progressively correcting the deformation while preserving patient’s mobility, a new implant (the “hemi-staple double”) allowing the action on both vertebral growth plates (GP) was developed and tested on a pig model. Its global effects on the spine were experimentally characterized. However, its local effects have not been evaluated.

**Aims**

To characterize the histomorphometry of the porcine GP’s for vertebrae instrumented with a double epiphyseal implant and for adjacent non-instrumented vertebrae.

**Methods**

Porcine vertebrae (T7-T9, from 11-weeks-old female pigs) previously instrumented for three months with a double epiphyseal implant (n=7) as well as control vertebrae (n=4) (Hachem B. et al., 2016) were prepared for growth rate (calcein injected one and seven days prior to sacrifice, GR) and for histomorphometric measurements including heights of the proliferative (PZH) and hypertrophic (HZH) zones, hypertrophic cells height (CH) and the number of proliferative chondrocytes per column (CNC). Each GP was analyzed in three regions: (1) non-instrumented; (2) central; (3) instrumented. The GR was calculated by measuring the distance between the two fluorescent calcein labels divided by six. Results were compared between the two groups for the three regions using a Student t-test (p<0.05).

**Results**

For the control group, a GR of 10.1±1.5 μm/day was found for the three regions. For the instrumented group, a GR of 8.6±1.7μm/day was measured. A significant decrease of 19.1% in the GR for region 3 was found in the instrumented group (8.1±1.8 μm/day) compared to the control group (10.0±1.3 um/day). No significant difference was found for GR in regions 1 and 2 between the instrumented (9.2±1.4 and 8.4±1.8 μm/day, respectively) and control groups (10.4±1.3 and 10.0±1.8 μm/day, respectively). Moreover, there was no statistically significant difference for the PZH for all regions. Significant reductions of 11.5% and 9.7% were found in regions 2 and 3, respectively, for the HZH of instrumented (49.4±4.6 and 51.0±5.5 μm, respectively) vs controls (55.8±4.0 and 56.5±4.1 μm, respectively). No significant differences were found for the CNC and the CH when comparing instrumented with controls.

**Conclusion**

This study allowed confirming the growth modulation effect of the double epiphyseal implant and quantifying the reduced GR within the instrumented region. This was translated at the GP histomorphometric level by a significant reduction in the HZH, the less mechanically rigid GP zone and the most sensitive to mechanical loading. This information is important to improve and develop new implants impeding the progression of musculoskeletal pathologies.

## P01 Position related discrepancy of trunk rotation measurements in idiopathic scoliosis

### Justyna Bloda^1^, Jakub Waś^2^, Dariusz Czaprowski^2^, Mateusz Kozinoga^3^, Tomasz Kotwicki^3^

#### ^1^Department of Physical Education, University of Physical Education, Warsaw, Poland; ^2^Department of Physiotherapy, Józef Rusiecki University College, Olsztyn, Poland; ^3^Department of Spine Disorders and Pediatric Orthopedics, University of Medical Sciences, Poznań, Poland

**Introduction**

One of the source the variability of angle of trunk rotation (ATR) could be the position of subject during the Adams forward bending test. The aim of the study was the assess the ATR variability related to the subject position.

**Methods**

The study comprised 97 children: 67 with idiopathic scoliosis (IS) and 30 healthy subjects, aged 9-18 years. In the scoliotic group the mean Cobb angle was 27.9° ± 13.1° for the thoracic and 23.6°± 11.2° for the lumbar spine, respectively. ATR was measured with Bunnell scoliometer during forward trunk flexion performed in standing position (Adams test). Maximal ATR value was evaluated for the proximal thoracic (Th1-Th4), main thoracic (Th5-Th12) and lumbar spine (L1-L5). The measurements were performed in the following variants of position: (1) forward bend using the reference position (feet placed at the hip width, hands joined, facing between feet), (2) forward bend in spontaneous position, (3) forward bend with feet parallel one against the other, (4) forward bend towards one foot, (5) forward bend with asymmetrical position of lower limbs in sagittal plane (sliding one foot 1 cm, 3 cm or 5 cm forward). For each subject and at each level of the spine (proximal thoracic, main thoracic and lumbar) the differences (changes) between ATR values measured in the reference position versus the foregoing variants were calculated. In each variant the difference was designated as ATR variability (∆ ATR).

**Results**

During forward bend towards the left foot the ∆ ATR was equal to or greater than 3° in 60% of subjects. There were no significant differences between ∆ ATR in proximal thoracic, main thoracic and lumbar spine for the habitual position (p=0.363) as well as during forward bend towards the right foot (0.277) or the left foot (p=0.635). The position of sliding one foot forward 3 cm or 5 cm resulted in ∆ ATR significantly higher in the lumbar than in the thoracic spine. There was no association between the position related ATR variability and number of curvatures, location of curvature, value of Cobb angle as well as skeletal maturity according to Risser (p>0.05). In children with IS, the ∆ ATR was significant higher during forward bend towards left foot (p=0.023) as well as during sliding forward 1 cm the right foot (p=0.018) and the left foot (p=0.012) compared to healthy children.

**Conclusion**

Sliding forward one foot causes the greatest ATR change in the lumbar spine. The position related variability of ATR is not related to parameters of scoliosis. In children with IS the position related variability of ATR is greater than in healthy children.

**Significance**

In clinical practice one should take into account the subject position during Adams test.

## P02 Study of genetic variants potentially involved in adolescent idiopathic scoliosis

### Hélène Mathieu^1^; Aurelia Spataru^1^; Vincent Cunin^2^; Sophie Ehresmann^1^; Norbert Ajeawung^1^; Justine Rousseau^1^; Mai Nguyen Thi Tuyet^1^; Virginie Saillour^3^; Soraya Barchi^1^; Julie Joncas^1^; Stefan Parent^1^; Philippe Campeau^1^; Florina Moldovan^1^

#### ^1^Research Center CHU Sainte Justine and Université de Montréal, Montréal, Canada; ^2^Hôpital Femme Mère Enfant Hospices civils de Lyon, 69677 Bron cedex, France; ^3^Centre de génomique clinique pédiatrique intégré Génome Québec and CHU Sainte-Justine, Montréal, Canada

**Aim**

Scoliosis is a complex disease characterized by phenotypic heterogeneity with unknown etiology. Some forms of scoliosis, including adolescent idiopathic scoliosis (AIS), seem to have genetic contribution. Epidemiologic approach confirmed genetic involvement of AIS and suggested different type of inheritance. In this study, the aim is to identify the disease-causing gene by performing exome sequencing of affected candidates.

**Our hypothesis** is that AIS is a polygenic disorder and that AIS definitely involves several genes.

**Materials and methods**

The selected families were mostly French-Canadian and have at least two members with AIS with a Cobb Angle up to 15 degrees. We performed exome sequencing of at least two affected people in each family. Identified candidate variants were validated by Sanger sequencing, and we studied their segregation with the disease in each family. To prioritize genes for functional studies, we studied pathogenicity prediction using *in-silico* analysis.

**Results**

20 families were studied: 8 had recessive inheritance of AIS and 12 had dominant inheritance. Exome sequencing of recessive inheritance families lead to identify 9 genes with 2 rare variants as possible candidates. 25 genes with rare variants in at least 2 families and over-represented compared to control population were selected for dominant inheritance families. After confirmation by Sanger sequencing and segregation analysis, 13 genes were considered for future functional studies: FN1 and VLDLR for recessive inheritance families; CA4, CASP4, GLS2, LAMB3, OGFR, P3H1, PTGS1, SPATA31E1, SSPO, SIPA1L3 and TCEB3B for dominant inheritance families. Variants of 7 genes: FN1, VLDLR, CA4, GSL2, OGFR, PTGS1 and SIPA1L3, have been predicted pathogenic.

**Conclusion**

Selected genes are potentially involved in AIS and could play a role in spine development by affecting bones and cartilage morphogenesis. Molecular consequences of these selected genes will be validated *in vitro* using cellular model derived from AIS patients and *in vivo,* by introducing the mutations by CRISPR-Cas9 in zebrafish.

**Significance**

Finding causative gene of AIS will help us understand the etiopathogenesis of AIS.

## P03 Genetic variant of SOCS3 gene is functionally associated with lumbar adolescent idiopathic scoliosis

### Jun Qiao^1,3^, Leilei Xu^1,3^, Bangping Qian^1,3^, Zezhang Zhu^1,3^, Jack Chun Yiu Cheng^2,3^,Yong Qiu^1,3^

#### ^1^Department of Spine Surgery, the Affiliated Drum Tower Hospital of Nanjing University Medical School, Nanjing, Jiangsu 210008, China; ^2^Department of Orthopaedics and Traumatology, Chinese University of Hong Kong, Hong Kong, China; ^3^Joint Scoliosis Research Center of the Chinese University of Hong Kong & Nanjing University, Nanjing, China

**Introduction**

Many investigators are realizing that the etiologies of thoracic AIS and lumbar AIS may be different. Some studies demonstrated that muscle development imbalance may be responsible for onset and progression of lumbar AIS. SOCS3 is one of the significant regulators of skeletal muscle development, and in vitro study showed that SOCS3 influences myoblast differentiation.

**Objective**

To investigate association between SOCS3 gene polymorphisms and the onset and progression of lumbar AIS and to further clarify its role in the regulation of SOCS3 expression in AIS patients.

**Methods**

Rs4969198 was genotyped in 476 lumbar AIS patients and 672 controls. The differences of genotype and allele distributions between patients and controls were calculated using Chi-square test. Paravertebral muscles were collected from 53 AIS, 23 congenital scoliosis (CS) and 18 lumbar disc herniation (LDH) patients. AIS patients were classified into three groups according to the genotypes of each SNP, and One-way ANOVA test was used to compare SOCS3 expression among different groups and genotypes.

**Results**

Patients were found to have a significantly higher frequency of GG than the controls (40.8% vs. 29.9%, OR= 1.36, p = 0.000), and the frequency of allele G was found to be remarkably higher in the patients than the controls (65.3% vs. 56.7%, OR= 1.15, p = 0.000). AIS patients had significantly less muscle expression of the SOCS3 than the CS patients (2.73 ± 2.17 vs. 4.62 ± 2.41, p=0.006) and the LDH patients (2.73 ± 2.17 vs. 4.12 ± 2.93, p=0.009). The SOCS3 expression was significantly correlated with the curve severity (r = 0.472, p = 0.014).

**Conclusions**

The SOCS3 gene is significantly associated with the development of lumbar AIS in Chinese population. Deceased expression of SOCS3 is associated

with larger severity of lumbar AIS.

**Significance**

This study indicated an unique etiology of lumbar AIS, which is different from thoracic AIS.

## P04 Arguments against a single genetic etiology of adolescent idiopathic scoliosis

### Ayesha Maqsood, David K. Frome, John F. Sarwark

#### Department of Surgery, Ann & Robert H. Lurie Children’s Hospital of Chicago, Chicago, IL, 60611, USA

##### **Correspondence:** John F. Sarwark (jsarwark@luriechildrens.org)

**Introduction**

Adolescent Idiopathic Scoliosis (AIS) is a common spinal condition affecting 2-4% of the population.[1] The etiology of AIS is considered multifactorial but remains under investigation. Numerous recent studies have attempted to identify a single genetic etiology of AIS.

**Objectives**

The objective of this brief study is to assess current evidence of a single genetic cause of AIS, including strength of evidence of studies to date and also those of genome-wide association studies (GWAS).

**Methods**

The study followed the PRISMA guidelines for conducting a systemic review. An extensive search, selection of studies, assessment of risk-of-bias and level of evidence grading was completed. A literature search was conducted using the following terms: adolescent idiopathic scoliosis, scoliotic, spinal curve, genetic, gene, etiology, polymorphisms. Articles were then assessed for risk-of-bias using a scoring system, 6 being low risk-of-bias and 0 being high risk-of-bias. The level of evidence grading was completed using criteria from the Oxford Centre for Evidence-Based Medicine. Based on the level of evidence, a grade of recommendation is provided on a scale of A (highest) to D (lowest).

**Results and conclusions**

From the search, 36 relevant articles were identified. After exclusions, 8 articles were included in the quantitative analysis; three had a risk-of-bias score of 4, three had a risk-of-bias score of 5 and two had a risk-of-bias score of 6. Next the level of evidence was assessed, a level of 2b was given for one of the articles and a level of 3b was given to the remaining 7 articles. All 8 articles received a grade of recommendation of B. Of the 8 studies analyzed, 4 of the studies are in favor of a genetic link for AIS and 4 studies are against. And 6 of the total 8 studies analyzed were GWAS.

Based on the available literature analyzed in this paper there is moderate evidence with a low risk-of-bias in favor of there not being a single genetic cause of AIS. There is also equally moderate evidence with a low risk-of-bias in favor of a genetic link with AIS but these studies were completed in Asian populations, accounting for a small percentage of phenotypic variance observed in AIS, leaving the etiology of the majority of AIS largely unknown. Therefore, further studies should be completed in broader populations to validate the observed gene associations to AIS.

**Significance**

The etiology of AIS being a single genetic cause is not supported by evidence to date.

**References**

1. Chettier R, Nelson L, Ogilvie JW, et al. Haplotypes at LBX1 have distinct inheritance patterns with opposite effects in adolescent idiopathic scoliosis. PLoS One. 2015;10(2): e0117708.

## P05 Vitamin D enhanced cellular responses of AIS patients derived primary osteoblasts and osteocytes to mechanical stimulation

### Jiajun Zhang^1,2^, Yujia Wang^1,2^, Carol Cheng^1,2^, Tsz-ping Lam^1,2^, Bobby K. W. Ng^1,2^, Jack C. Y. Cheng^1,2^, Wayne Y. W. Lee^1,2^

#### ^1^Department of Orthopaedics and Traumatology, SH Ho Scoliosis Research Laboratory, The Chinese University of Hong Kong, Shatin, NT, Hong Kong; ^2^Joint Scoliosis Research Center of the Chinese University of Hong Kong and Nanjing University, Department of Orthopaedics and Traumatology, Faculty of Medicine, The Chinese University of Hong Kong, Shatin, Hong Kong.

##### **Correspondence:** Wayne Y. W. Lee (waynelee@ort.cuhk.edu.hk)

**Introduction**

Adolescent idiopathic scoliosis (AIS) is a three-dimensional spinal deformity closely associated with altered bone qualities. Improving bone qualities could be a potential treatment target to prevent curve progression. We previously investigated if whole body vibration could promote bone qualities in AIS girls, which the outcome was further enhanced by higher basal serum level of 25(OH) vitamin D (VitD). Osteoblasts are bone forming cell. Its descendent cells, osteocytes, transduce mechanical stimulation to biological signaling to orchestrate bone homeostasis. Therefore, we hypothesized that VitD could improve response of osteoblasts and osteocytes to mechanical stimulation in AIS.

**Objectives**

To characterize cellular activities of AIS osteoblasts and osteocytes under concurrent treatment of VitD and vibration.

**Method**

Patients-derived primary osteoblasts were isolated from iliac crest trabecular bone biopsies harvested intraoperatively from 10 AIS patients undergoing spinal fusion. The primary osteocytes culture were established by embedding the osteoblasts in 3D collagen type I gel for 7 days. The cells were with/without vibration (0.3g, 35 Hz, 20 minutes daily) under different concentration of 1α, 25(OH) VitD_3_ at concentration of 0, 0.01, 0.1 uM for 7 days. mRNA level of representative osteoblast and osteocyte markers were determined with qPCR.

**Results**

VitD increased the response of AIS primary osteoblast and osteocytes to vibration in a concentration-dependent manner as indicated by up-regulation of *Runx2, Alp, Spp1, Bglap* and *Vdr* in primary osteoblasts, and *Phex, Cx43* and *E11* in primary osteocytes. Significant difference was observed in Vit-D supplementation at concentration of 0.1uM.

**Discussion and significance**

This is the first study on the effect of VitD on the cellular response of AIS primary osteoblasts and osteocytes to mechanical stimulation. Our findings suggest that higher level of VitD could enhance the cellular response to mechanical stimulation, implying a sufficient serum VitD level might be important factor to bone qualities improvement in AIS patients.

**Acknowledgements**

This project is supported by Hong Kong RGC/GRF (Ref. no. 14116415) and HMRF 04152176.

## P06 Restrained differential growth: The initiating event of adolescent idiopathic scoliosis?

### Tom Crijns, Agnita Stadhouder, Theodoor Smit

#### Department of Orthopedics, VUMC, 1081 HV, Amsterdam, The Netherlands

**Introduction**

Despite decades of research, there is no generally accepted theory on the physical origin of the severe spinal deformations seen in AIS. The prevailing theories tend to focus on left-right asymmetry, rotational instability, or the sagittal spinal profile in idiopathic scoliosis.

**Objectives**

The purpose of this study was to explore a new hypothesis suggesting that the curvatures seen in adolescent idiopathic scoliosis (AIS) originate from restrained differential growth between the vertebral column and the surrounding musculo-ligamentary structures.

**Methods**

We test our hypothesis with a physical model of the spine that simulates growth, counteracted by ligaments and muscles, modeled by tethers and springs. Growth of the spine is further restrained by an anterior band representing the thorax, the linea alba, and abdominal musculature. We also explore literature in search of molecular mechanisms that may induce differential growth.

**Results**

Differential growth in the restrained spine model first induces hypokyphosis and mild lateral bending of the thoracic spine, but then suddenly escalates into a scoliotic deformity, consistent with clinical observations of AIS. The band simulating the ventral structures of the body had a pivotal effect on sagittal curvature and the initiation of lateral bending and rotation. In literature, several molecular mechanisms were found that may explain the occurrence of differential growth between the spine and the musculo-ligamentary structures.

**Conclusion**

While AIS is a three-dimensional deformation of the spine, it appears that restrained differential growth in the sagittal plane can result in lateral bending and rotation without a pre-existing left-right asymmetry. This supports the concept that AIS may result from a growth imbalance rather than a local anatomical defect.

## P07 Growth pattern of lower limbs is not altered in AIS patients: A full-body quantified analysis

### Hongda Bao, Shibin Shu, Qi Gu, Yuancheng Zhang, Zezhang Zhu, Zhen Liu, Yong Qiu

#### Nanjing Drum Tower Hospital, Nanjing, 210008, China

**Introduction**

The abnormal spinal overgrowth was confirmed in adolescent idiopathic scoliosis (AIS) pts which may be attributed to secondary change. However, the growth velocity in different maturity status and the final length of lower limbs were not investigated in AIS pts.

**Objective**

To investigate if the growth pattern of lower limbs is altered in AIS patients.

**Methods**

This is a prospective analysis of AIS pts and asymptomatic subjects. The inclusion criteria for AIS were as follow: female, thoracic Cobb angle 20~60°, age≥10y/o, with full-body images. Major Cobb angle, length of spine (LOS), length of lower limbs (LLL), height of pelvis (HOP) were measured. The following ratios were calculated: Ratio_SL_=LOS/LLL, Ratio_SP_=LOS/HOP, Ratio_PL_=HOP/LLL. The maturity statues was classified into 3 groups: pre-peak height velocity (PHV) defined as Risser 0 & Open triradiate cartilage (TC), during PHV (Risser 0 & closed TC), post-PHV (Risser≥1). The radiographic parameters and ratios were compared between different maturity status.

**Results**

A total of 49 AIS pts (mean age 13.4 y/o, Cobb angle 33.1°) and 30 asymptomatic adolescents (mean age 12.5 y/o) were enrolled. 20 AIS pts were Risser 0 and 29 Risser≥4, 18 normal subjects were Risser 0 and 12 Risser≥4. The Ratio_SL_ and Ratio_SP_ were significantly larger in AIS pts with Risser≥4 compared to the normal subjects (p=0.037 and 0.026, respectively), indicating longer final spinal length in AIS. While the Ratio_PL_ was similar between AIS and normal subjects in both Risser 0 and Risser≥4 group. The change of ratios from pre-PHV to post-PHV showed similar trends between AIS and normal subjects: the Ratio_SL_ and Ratio_PL_ were significantly smaller in during-PHV group (all p<0.001), indicating that the PHVs of spine and pelvis are later than the PHV of lower limbs.

**Conclusion**

The final length of lower limbs was similar, while the peak growth of lower limbs is earlier than the pelvis and spine in both AIS and normal controls. The results indicated that the growth pattern of lower limbs is not altered in AIS patients.

**Significance**

This study found that the growth pattern of lower limbs may not be altered in AIS patients, indicating that length of lower limbs could serve as a potential reference value for AIS patients.

## P08 Relationship between abnormal sagittal global alignment and severity of vertebral fracture in patients with osteoporosis

### Zongshan Hu, Gene C.W. Man, Sheung Wai Law, Anthony Kwok, Jack C.Y. Cheng

#### Department of Orthopaedics and Traumatology, Faculty of Medicine, The Chinese University of Hong Kong, Prince of Wales Hospital, Hong Kong, China

**Introduction**

Osteoporosis is a major health condition characterized by low bone mass and diminished bone strength, leading to skeletal fragility and increase risk of hip, wrist and spinal fracture [1]. Osteoporotic vertebral compression fractures (VCF) of the spine is very common and at times underdiagnosed [2] and was found to be associated with the increased mortality, morbidity, and overall decline in quality of life in the elderly [3]. Studies have shown that osteoporotic patients with vertebral fracture had significantly higher thoracic kyphosis and lower lumbar lordosis in the spinal sagittal alignment profile [4]. However, the influence of vertebral fractures on whole-body compensatory mechanism, including pelvic retroversion and knee flexion, remains unclear. The aim of this study was to compare the global body sagittal alignment and quality of life in elderly osteoporotic subjects with and without vertebral compression fractures, and to investigate the relationship between the changes of these sagittal alignment with the severity of the fracture.

**Materials and methods**

A consecutive series of 72 osteoporotic subjects with BMD documentation were prospectively enrolled. Clinical assessment, including age, body height and weight, were recorded. Global sagittal alignment was taken with EOS^®^ low dose X-ray imaging system. The number and location of VCF were assessed, and the severity of VCF was evaluated by Spinal Deformity Index [5]. Measurement on global sagittal alignment was done by using T1 pelvic angle (TPA) and global sagittal angle (GSA) (Fig. 1). Quality of life assessment was completed for each subject by validated questionnaires: Oswestry Disability Index (ODI) and Short-form (SF)-12.

**Results**

The TPA and GSA were significantly correlated with SF-12 and ODI in osteoporotic patients. The patients with VCF were found to have significantly higher TPA and SVA. The number and severity of VCF significantly correlated with global sagittal alignment. Discriminative value for identification of patients with at least one VCF, assessed by Area Under the Curves (AUCs) was 0.661 and 0.766 for TPA and GSA, respectively. Using multivariate analysis, parameters significantly associated with abnormal global alignment (GSA) were the number (OR=2.96, P=0.01) and severity of VCF (OR=4.23, P<0.0001) and age (OR=1.07, P=0.02) (Table 1).

**Discussion**

Similar to previous studies on adult spinal deformity, the parameters of TPA and GSA were significantly correlated with quality of life measurement in osteoporotic patients. In addition, there was differences in TPA and GSA found between osteoporotic patients with and without VCF. This is the first study to report deteriorated global alignment in osteoporotic patients with VCF. There was a negative effect of the number and severity of VCF on global alignment. Further longitudinal study based on these early findings with a larger cohort would be important in the future to define more quantitatively the relationship that might inform better the clinical prognostication, prevention and treatment strategy.

**Significance**

The osteoporotic patients with VCF had a worse overall global sagittal alignment. The number and severity of VCF are strong determinants of global sagittal balance. The osteoporotic patients with a poorer sagittal global alignment may imply more severe vertebral fracture. Based on our current result, the changes in global sagittal alignment may be a potential prognostic indicator for vertebral facture in osteoporotic patients.

**References**

[1] Jackson S et al. Osteoporos Int 2010

[2] Ettinger B et al. J Bone Miner Res, 1992

[3] Bliuc D et al. JAMA, 2009

[4] Lee J et al. Eur Spine J, 2013

[5] Kerkeni S et al. Osteoporos Int, 2009

**Table 1 (abstract P08).** Comparisons between the patients with and without vertebral compression fracture (VCF) in terms of baseline characters and radiographic measurements

Variables	VCF (n=37)	non-VCF (n=35)	*p* value
Age (yrs)	71.8±9.3	69.3±9.8	0.34
BMI (kg/m^2^)	23.3±3.7	24.2±3.8	0.12
Lumbar spine BMD (g/cm^2^)	0.849±0.17	0.791±0.13	0.02
Femoral neck BMD (g/cm^2^)	0.736±0.11	0.718±0.09	0.11
T1 pelvic angle (°)	26.6±10.9	16.5±7.4	<0.001
Global sagittal angle (°)	25.7±6.4	18.2±6.9	0.001

**Fig. 1 (abstract P08).**
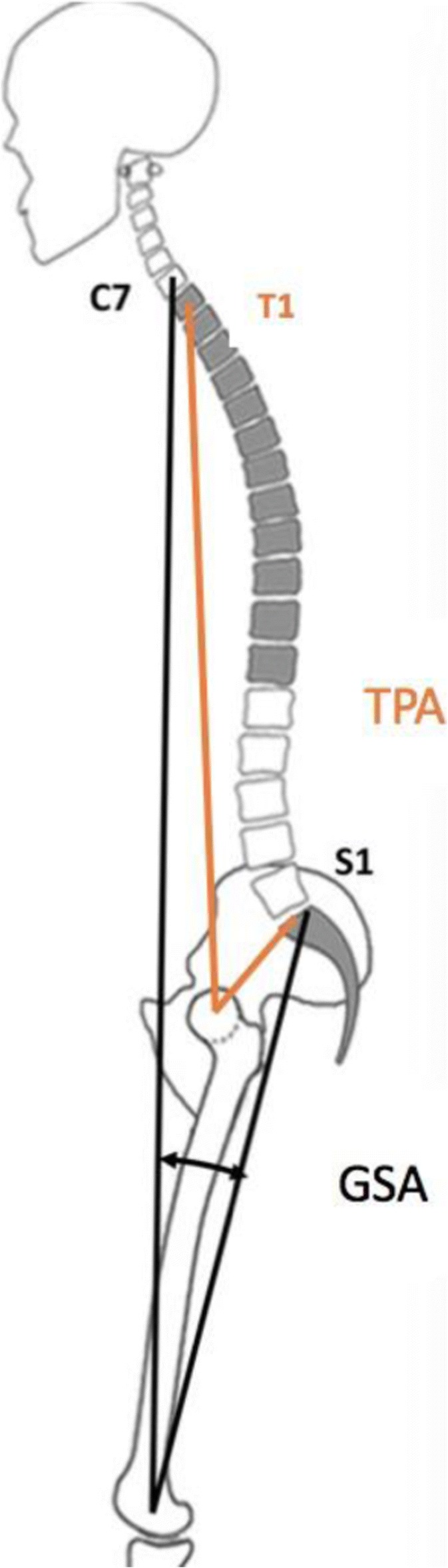
The T1 pelvic angle (TPA) is defined as the angle between the line from the femoral head axis to the centroid of T1 and the line from the femoral head axis to the middle of the S1 superior endplate. The Global sagittal angle (GSA) was defined as the angle formed by a line from the midpoint of the 2 distal femoral condyles to the center of C7, and a line from the midpoint between the 2 distal femoral condyles to the posterior superior corner of the S1 endplate

## P09 Validation of a novel 3D imaging and surface topography mobile App to assess spinal posture and mobility

### Gokulakannan Kandasamy^1^, Josette Bettany-Saltikov^2^, Paul Van-Schaik^1^, John Dixon^2^

#### ^1^School of Social Sciences Humanities and Law, Teesside University, Middlesbrough, UK; ^2^School of Health and Social Care, Teesside University, Middlesbrough, UK

**Introduction**

Spinal deformities such as scoliosis, hyper kyphosis, increased or decreased lordosis as well as marked back asymmetries are all potential sources of spinal pain. The measurement of 3D spinal posture and mobility is important for both the clinical and research context (epidemiological and clinical studies). Within clinical practice, monitoring changes in back shape and spinal mobility over time is fundamental for patients with spinal pain. Although various techniques are available to measure spinal posture and kinematics in a research or lab-based environment, there is still a need for a less expensive, portable, mobile-based application, to record 3D spinal posture and mobility with precision and accuracy.

**Objective**

The main aim of this study was to validate a novel 3D imaging and surface topography mobile App to assess spinal posture and mobility.

**Participants**

Twenty-five volunteers (16 males, 9 females, age range of 21- 45 years) with no history of spinal pain participated in this study.

**Instrumentation**

In this study we have used the commercially available IPad based 3D mobile scanning tool ‘Structure Sensor^TM^’ to capture the shape of the back as well as the whole participants’ body. This sensor consists of two different cameras (Color video (red-blue-green) and the depth camera). This sensor along with the normal IPad camera provides real-time anatomical landmarks and reconstructs the whole back and body shape using the triangulation method.

**Procedure**

For each subject, an optical motion capture system and 3D mobile scanning tool were simultaneously used to capture three trials of standing, bending forward, backwards, bilateral side flexion and rotation. The 3D mobile data was then processed in the open source software to measure lumbar lordosis, thoracic kyphosis, bilateral side flexion and bilateral rotation. These variables were also measured for the optical motion captured data using commercially available Vicon software.

**Result(s) and discussions**

Comparison of the mean results between the two measurement systems in standing showed no statistically significant differences in lumbar lordosis, thoracic kyphosis, lumbar flexion and thoracic flexion (p value 0.35, 0.14, 0.18 and 0.45). Side flexion of lumbar and thoracic spine also showed statistically non- significant difference. For rotation, the two devices yielded significantly different group mean values and the limits of agreement were unacceptably wide (-35^0^ to 39^0^; -26^0^ to 61^0^; -50^0^ to 95^0^ and 24^0^ to -63^0^). The authors believe that these results may have been caused by different methods of angle calculation between the app and vicon.

**Conclusion(s) and significance**

The app has been shown to be valid for the measurement of most variables measured. Given that it is inexpensive, extremely portable and simple to setup, this tool has a high potential to be used within clinical practice for monitoring spinal posture and mobility in the sagittal and frontal plane.

**Keywords:** Spinal posture, mobility, ROM, surface measurement, depth camera, mobile application

## P10 A pilot study on using EOS 3D reconstruction parameters to estimate the spinal flexibility on children with AIS

### Mahdieh Khodaei^1^, Lawrence H Le^1^, Edmond Lou^1,2^

#### ^1^Department of Radiology and Diagnostic Imaging, University of Alberta, Edmonton, Canada; ^2^Department of Electrical and Computer Engineering, University of Alberta, Edmonton, Canada

##### **Correspondence:** Edmond Lou

**Introduction**

Adolescent idiopathic scoliosis (AIS) is a 3D spinal deformity. Spinal flexibility is an important parameter to assist treatment planning for children with AIS. Although ultrasound (US) imaging has been proposed to estimate spinal flexibility, it is not always available in most of scoliosis clinics. On the other hand, low-dose bi-planar EOS system is becoming common to image scoliosis. This system allows 3D reconstruction of spine that provides 3D deformity information which may allow clinicians to estimate spinal flexibility.

**Objective**

To investigate if 3D reconstruction parameters from the EOS system were able to estimate spinal flexibility on children with AIS.

**Methods**

Forty-two (35 F; 7 M) AIS subjects were recruited. All subjects had the biplanar EOS images as well as scanned by a delicate US machine in two positions including standing and maximal prone side-bending either only toward right or left, or both sides. One trained rater reconstructed all 42 EOS images. Three parameters including the posteroanterior (PA) Cobb angle, the maximal axial vertebral rotation (AVR), and the sagittal balance either on the thoracic (kyphosisT1/T12) or lumbar (lordosis L1/L5) regions were obtained after reconstruction. The same rater measured the proxy Cobb angle on standing and side-bending US images. Spinal flexibility was calculated based on the equation: (proxy PA Cobb – proxy side bending) / (proxy PA Cobb). The thoracic and lumbar regions were analyzed separately. A step-wise linear regression analysis was used to find the association of the 3 parameters with flexibility and the Pearson correlation coefficient (r) were reported.

**Results and discussions**

Sixty-three curves were identified on both EOS reconstructions and US images. The PA Cobb angle, AVR, kyphotic angle (KA), and lordotic angle (LA) at the thoracic and lumbar regions were (24.9±0.2, 7.9±4.5, 29.4±12.0), and (23.4±8.7, 8.2±5.1, 48.9±10.3), respectively. For the thoracic region (n = 40), the r values for Cobb, AVR, and KA associated with the flexibility were -0.60, -0.28, 0.21, respectively. For the lumbar region (n =23), the r values for Cobb, AVR, and LA were -0.72, -0.34, -0.26, respectively. Since the Cobb is the strongest predictor for the flexibility, using the Cobb + AVR, Cobb + KA and Cobb + AVR + KA provided the r value -0.60 for all 3 models. Similarly, for the lumbar region, the r value for Cobb + AVR, Cobb + LA and Cobb +AVR + LA was also -0.72.

**Conclusion and significance**

Among the 4 parameters, the Cobb angle showed a moderate association with the spinal flexibility based on the lateral bending method. As the AVR, kyphotic angle and lordotic angle more described the deformity in transverse and sagittal planes, they may reflect the flexibility in the other 2 planes. Therefore, future study should use ultrasound to capture the spinal information while subjects rotate along the gravity axis to the right and left as well as bend forward and backward.

## P11 Magnetic resonance imaging-based morphological changes of paraspinal muscles in girls with adolescent idiopathic scoliosis

### Kwong Hang Yeung^1^, Gene Chi Wai Man^2,3^, Shi Lin^1^, Steve Cheuk Ngai Hui^1^, Tsz Ping Lam^2^, Bobby Kin WahNg^2^, Jack Chun Yiu Cheng^2^, Winnie Chiu Wing Chu^1^

#### ^1^Department of Imaging and Interventional Radiology, Faculty of Medicine, The Prince of Wales Hospital, The Chinese University of Hong Kong, Shatin, Hong Kong SAR, China; ^2^Department of Orthopaedics and Traumatology, Faculty of Medicine, The Prince of Wales Hospital, The Chinese University of Hong Kong, Shatin, Hong Kong SAR, China; ^3^Department of Obstetrics and Gynaecology, Faculty of Medicine, The Prince of Wales Hospital, The Chinese University of Hong Kong, Shatin, Hong Kong SAR, China

**Introduction**

Several studies have reported abnormalities in biochemical, electromyographic activity (EMG) and histological changes of paraspinal muscles in AIS patients. However, these studies only had qualitative data without the comparison with healthy controls. Change of muscle mass and mean density at the lumbar region have also been described for scoliotic spines. All these findings suggested that imbalance of paraspinal muscles in AIS patients could be a contributing factor to the development of more severe scoliotic curve.

**Objectives**

To investigate and compare any morphological differences in paraspinal muscles between AIS patients with different curve severities and healthy controls.

**Methods**

T2-weighted MR images with multi-planar reconstruction were acquired in 64 Chinese female adolescents including AIS girls with primary right-side thoracic curves (17 mild to moderate, 24 severe curves), and 23 age-matched controls. In AIS subjects, measurements of the paraspinal muscles were taken on both concavity and convexity of the scoliotic curve starting from two vertebral levels above and two vertebral levels below the scoliotic apex. Morphological assessments of the multifidus (MF) and erector spinae (ES) muscles on both sides were made including signal intensity, area and fat deposition using ROI based approach, manual tracing and thresholding technique respectively. Same parameters were measured in healthy controls at matched vertebral levels. Ratios were taken as concave/convex (for AIS) or left / right (for controls). One-way ANOVA test with Post Hoc test of Bonferroni correction was used for comparison. Cobb angle was correlated with anthropometric parameters using pearson correlation test.

**Results & discussion**

In AIS, signal intensity was significantly higher at the multifidus of paraspinal muscles on the concave side, when compared with the controls. Higher ratios of signal intensity and fatty components were noted within the concave muscle when compared with the convex side (P-value <0.01). A significant correlation was found between Cobb angle and signal intensity within the concave muscle at the apical level.

**Conclusion & significance**

Increased signal intensity and fatty components are asymmetrically present in the paraspinal muscles at the apex level in AIS. Our results showed higher intensity in paraspinal muscle at the concavity of the curve in AIS when compared with controls. There was also significant linear correlation between abnormal muscle signal and scoliotic curve. Above features are suggestive of altered muscle composition in the concave paraspinal muscle, possibly due to distortion and prolonged stretching of the paraspinal muscle caused by the spinal deformity.

## P12 Results of the Maastricht brace in the treatment of adolescent idiopathic scoliosis

### Dirk Schrander, Chris Arts, Helma Voets, Mark van den Boogaart, Paul Willems, Lodewijk van Rhijn

#### Department of Orthopaedic Surgery, Maastricht University Medical Center, Maastricht, Research School CAPHRI, Maastricht, The Netherlands

**Introduction**

The Maastricht brace (M-brace) was developed to improve patient compliance and associated efficacy of brace treatment in adolescent idiopathic scoliosis (AIS). The main features are an anterior closure, elastic thoracic pelotte and comfortable material, so the emphasis is on wearability and comfort for the patient, whilst retaining corrective pressure function. Initial pressure measurements in the M-brace revealed a higher corrective pressure as compared to the Boston brace, and a better patient reported quality of life as measured with the SRS 22 and Brace questionnaire. Earlier results of the efficacy in terms of curve correction of the M-brace in AIS were promising, with an average in-brace curve correction of 48%. The aim of this study was to evaluate the end-term radiological results of patients treated with the M-brace, with a minimum of one year follow up after stop of brace wearing.

**Methods**

All patients with AIS and a Cobb angle between 25 and 45 degrees, who had been treated with the M-brace since January 2011, were included in this retrospective single-center study. The correction effectiveness of the brace was evaluated by comparing the primary curves on standing postero-anterior full spine radiographs with and without M-brace. The end-term correction of the Maastricht brace was defined as the primary curve measured on standard postero-anterior full spine radiographs taken one year after stop of brace wear. The effectiveness of bracing was defined as prevention of curve progression necessitating surgical intervention.

**Results**

A total of 39 patients were included. There were 31 patients with a primary thoracic curve, 8 patients with a primary lumbar curve. The predominant Lenke classification was type 1 (21), 2 (7) and 3 (8). The average primary curve Cobb angle was 35.2° ± 9.7°. The average primary curve angle in bending x-rays was 14.9° ± 6.8°. In the M-brace the primary curve was 24.2° ± 8.3°. This is an in-brace correction of 46%. The average primary curve Cobb angle at end-term of bracing was 37.7° ± 12°. There were 9 patients in whom the curves progressed to surgical magnitudes during brace wear. Therefore, the effectiveness of bracing primary thoracic adolescent idiopathic scoliosis with the M-brace was 77%.

**Conclusions and significance**

These first end-term results demonstrate an overall adequate correction effectiveness with the M-brace, with end term results comparable to those of the Boston brace in literature. Future compliance research is imminent in order to objectify the expected wearability and comfort.

## P13 Optimal thickness for additive manufactured brace

### Robert Rizza^1^, Xue-Cheng Liu^2^, John Thometz^2^, Vince Anewenter^1^

#### ^1^Department of Mechanical Engineering, Milwaukee School of Engineering, Milwaukee, WI, USA; ^2^Department of Orthopaedic Surgery, Medical College of Wisconsin, Milwaukee, WI, USA

##### **Correspondence:** Robert Rizza; Xue-Cheng Liu

**Introduction**

Considering implementation of computerized models which predict the functional behavior of the brace a priori, questions are raised as why these same computerized methods cannot be used to manufacture the brace.

There is much interest in using Additive Manufacturing (AM), a 3D printing method, as this approach is a minimal hands-on very accurate method that may generate brace with a very short technician involvement time (as little as 0.5-2.5 hours). However, to make full benefit of AM the brace needs to be designed for manufacturing with AM in addition to being designed to provide the proper constraint forces.

Traditionally manufactured braces are made of Polyethylene (PE). Many original AM materials have less endurance, are more brittle, and have less flexibility and rigidity than PE. However, there are many new AM materials which compare favorably to PE.

**Objectives**

This study was motivated by the goal to develop a brace manufactured using AM with an optimal AM material that generates the same constraint forces as a traditional brace made of Polyethylene.

**Materials and methods**

Data of a patient’s torso and spinal geometry was used in a prior validated spine Finite Element Analysis (FEA) model to predict the maximum allowable brace deformation for the patient (18.57 mm). Computer Aided Design (CAD) was employed to create a computer model of the brace design. The design was optimized with cut-outs to remove unnecessary material and further proper patient breathing and derotation of the spine. Various AM materials were investigated and mechanically tested to determine the optimal one. The AM and traditional brace were designed so that the deformation does not exceed 18.57 mm thereby generating the needed constraint forces. A brace was constructed using AM (Fortis 900®, Stratsys, Inc.) and tested in the laboratory to validate the FEA and determine the brace response.

**Results and discussions**

Polylactic Acid (PLA), a biomaterial, was found to be the best AM material. The FEA prediction for the AM brace thickness was a 2.5 mm. The thickness of the traditional brace was 4 mm. The surface area and hence weight of the AM was 56% less than the traditional brace. For the AM brace, the laboratory testing indicated a 7% difference between the FEA deformation (18.57 mm) and the testing (17.19 mm).

**Conclusion and significance**

Both AM and traditional designs will generate the proper constraint forces to maintain spinal correction. But AM design is thinner (2.5 mm vs. 4 mm) and hence less weight. It was constructed with a short technician time (0.5 hours) and had 56% less material while allowing for better breathing and rotation of the torso.

## P14 Predictors of failure in bracing adolescent idiopathic scoliosis

### Dirk Schrander, Chris Arts, Helma Voets, Mark van den Boogaart, Paul Willems, Lodewijk van Rhijn

#### Department of Orthopaedic Surgery, Maastricht University Medical Center, Maastricht, Research School CAPHRI, Maastricht, The Netherlands

**Introduction**

Several studies have been published demonstrating predictors of failure in bracing of adolescent idiopathic scoliosis (AIS). Initial correction magnitude, age of onset, curve pattern, curve magnitude before peak growth and low Risser scores before bracing have all been described as predictors. However, there are few studies describing the effect of prior vertebral rotation on brace effectiveness. The Maastricht brace (M-brace) was developed to improve patient compliance and associated efficacy of brace treatment in AIS. The main features are an anterior closure, elastic thoracic pelotte and comfortable material use. Because of the thoracic elastic pelotte, a stronger vector force is expected to control thoracic rotation.

**Methods**

All patients with mild to moderate AIS, who had been treated with the M-brace between January 2011 and 2015, were included in this retrospective single-center study. The correction effectiveness of the brace was evaluated by comparing the primary curves on standing postero-anterior full spine radiographs with and without M-brace. The success of bracing was defined as prevention of curve progression necessitating surgical intervention. The rotation before and in bracing was objectified using the Nash-Moe classification, ranging between 0 (no rotation) and 4 (more than 90° rotation). Other radiological parameters in the anteroposterior and sagittal profile were also evaluated and included in the statistical analysis to rule out other possible predictors of failure.

**Results**

A total of 39 patients were included. There were 31 patients with a primary thoracic curve, 8 patients with a primary lumbar curve. The predominant Lenke classification was type 1 (21), 2 (7) and 3 (8). The average primary curve Cobb angle was 35.2° ± 9.7°. The average Nash Moe classification was 1.3 (0.8). The average primary curve angle in bending x-rays was 14.9° ± 6.8°. In the M-brace the primary curve was 24.2° ± 8.3°. The average primary curve Cobb angle at end-term of bracing was 37.7° ± 12°. There were 9 patients in whom the curves progressed to surgical magnitudes during brace wear. In these 9 patient the average Nash-Moe score was 2 (0.5), significantly higher than the effective brace population (1,1 (0,7), p < 0.05). The average primary curve cobb angle within this group was also higher, with an average of 44.7 (9.6) compared to 32.5 (9.3, P< 0.05). There was a striking difference between the Thoracic Sagittal modifier, averaging a smaller cobb angle of 13.3 (8) compared to the effective brace population of 20.6 (11.1), P<0.05.

**Conclusions and significance**

The predictors of failure in bracing adolescent idiopathic scoliosis are important in determining for which patient it is useful to undergo a brace treatment. This research emphasizes that patients with a Nash-Moe classification of 2 or more at time of diagnosis, high cobb angle and low thoracic sagittal modifier should be monitored more closely because of risk of progression.

## P15 Vertebral modulation during bracing - the trigger for rapid curve progression in non-dystrophic neurofibromatosis type 1 scoliosis

### Benlong Shi^1,3^, Yang Li^1,3^, Zhen Liu^1,3^, Xu Sun^1,3^, Zezhang Zhu^1,3^, Yong Qiu^1,3^, Tsz Ping Lam^2,3^, Jack C. Y. Cheng^2,3^

#### ^1^Spine Surgery, the Affiliated Drum Tower Hospital of Nanjing University Medical School, Nanjing, China; ^2^Department of Orthopaedics and Traumatology, Chinese University of Hong Kong, Hong Kong, China; ^3^Joint Scoliosis Research Center of the Chinese University of Hong Kong & Nanjing University, Nanjing, China

**Introduction**

Bracing treatment is often recommended for non-dystrophic NF1 scoliosis since it is similar to the idiopathic curve. The vertebral modulation has been widely found in non-dystrophic NF1 scoliosis, defined as the tendency of acquiring dystrophic changes during longitudinal follow-up. However, the association between vertebral modulation and rapid curve progression during bracing treatment in NF1 scoliosis is rarely reported.

**Objective**

To investigate the curve evolution of patients with NF1 scoliosis undergoing bracing treatment, and to evaluate the association between vertebral modulation and rapid curve progression in NF1 scoliosis.

**Method**

The patients were regularly followed up for at least 2 years at an interval of 4-6 months and those patients who presented vertebral modulation were included in this study. The age, Cobb angle and rate of curve progression were measured for each visit. Vertebral modulation was observed in all patients, of which the age was recorded. The rapid curve progression phase was defined as >10°/y.

**Results**

A total of 15 patients with average ages of 7.1±2.1 years at initial visit and 9.9±2.2 years at the last follow-up were enrolled. The average Cobb angle were 42.7±10.1 ° at initial visit, which increased to 61.7±14.4 ° at the last follow-up. 10 patients finally underwent surgery intervention and the remaining 5 patients were still under observation. The average age of modulation was 8.4±1.8 years. The average rate of curve progression was 5.1±0.6°/y before modulation and 10.2±2.4°/y after vertebral modulation, respectively (P=0.001). Totally, 0 and 10 patients were identified to be with rapid curve progression phase before and after modulation, and significant difference was found between 2 groups (P<0.001).

**Conclusions**

The patients with non-dystrophic NF1 scoliosis are in a high risk of rapid curve progression after vertebral modulation. The vertebral modulation during bracing thus serves as a trigger for rapid curve progression in non-dystrophic NF1 scoliosis.

## P16 Evaluation of accuracy, precision and optimal parameters of a 3D scanner in acquiring body contour of patients with adolescent idiopathic scoliosis

### Kenwick Ng^1^, Edmond Lou^1,2*^, Kajsa Duke^3^

#### ^1^Department of Biomedical Engineering, University of Alberta, Edmonton, Alberta, T6G 2V2, Canada; ^2^Department of Electrical Engineering, University of Alberta, Edmonton, Alberta, T6G 1H9, Canada; ^3^Department of Mechanical Engineering, University of Alberta, Edmonton, Alberta, T6G 1H9, Canada

##### **Correspondence:** Edmond Lou (elou@ualberta.ca)

**Introduction**

The traditional method in fabricating a brace for treating Adolescent Idiopathic Scoliosis (AIS) requires significant labour. A novel approach to design a brace with real-time feedback involving a custom brace design frame and ultrasound scanner to measure scoliotic curvature has been developed. To improve fabrication efficiency, usage of a 3D handheld scanner to acquire the surface contours of the patient’s torso is proposed. This study proposes to evaluate the accuracy and precision of this technology before applying it in scoliosis clinics.

**Objective**

To evaluate the accuracy and precision of the 3D Spectra Scanner along different dimensions at varying scan distances to acquire body shape.

**Methods**

A 3D Spectra Scanner was used as the torso scanner to determine its accuracy and precision. A set of 3 Optitrack Prime 13W motion capture cameras with a calibrated accuracy of 0.10mm were used to capture specific dimensions on the torso to compare with the Spectra Scanner. Five linear dimensions including A) maximum lateral distance from waist-to-rib (103mm), B) waist thickness (105mm), C) waist depth (132mm), D) vertical distance between axilla and ASIS (240mm) and E) maximum brace length (495mm) were measured on a patient foam mold. Three scan distance ranges 1) close 26-38cm, 2) optimal 38-48cm and 3) far 48-58cm based on light indicators were tested. For each measurement, the distance between two 6.8mm reflective markers which were placed on the torso was calculated from the Optitrack and Spectra scanner software. Five scans were performed at a scan distance with 5 dimensions and each measured five times, coming to 375 (5 x 5 x 3 x 5) measurements. Root Mean Squared (RMS) of deviations between motion capture and scanner measurements were used to determine accuracy.

**Results and discussions**

The average accuracy of each measurement was a)0.97mm, b)3.31mm, c)1.46mm, d)1.25mm, e)4.92mm. The precision of each measurement was a)1.08mm, b)1.08mm, c)0.85mm, d)0.83mm, e)3.23mm. The largest dimension had the poorest accuracy at 4.92mm while the smallest dimension had the best accuracy at 0.97mm. Probability value for mean accuracy (Ap) and precision (Pp) with two tail Student T-Test between close/optimal (Ap=0.80, Pp=0.30 >0.05), close/far (Ap=0.43, Pp=0.16 >0.05), and optimal/far (Ap=0.70, Pp=0.37>0.05) scan distances showed no significant difference. The results demonstrated 3D scanner accuracy is length rather than scan distance dependent. Further study evaluating the (X,Y,Z) components of each dimension would be valuable for brace construction.

**Conclusions and significance**

The accuracy and precision of Spectra 3D scanner are 4.92mm and 3.23mm overall. Accuracy and precision are not scan distance dependent, but accuracy is length dependent. These findings provide confidence for orthotists to use the 3D scanned torso in building orthosis either manually or applying 3D printing technology. Further clinical efficiency study is needed.

## P17 Association of the in-brace Cobb angle correction with axial vertebral rotation and torsion correction on children with AIS

### Kenwick Ng^1^, Edmond Lou^1,2*^, Douglas Hill^3^, Kajsa Duke^4^

#### ^1^Department of Biomedical Engineering, University of Alberta, Edmonton, Alberta, T6G 2V2, Canada; ^2^Department of Electrical Engineering, University of Alberta, Edmonton, Alberta, T6G 1H9, Canada; ^3^Alberta Health Services, Edmonton, Alberta T5G 0H1, Canada; ^4^Department of Mechanical Engineering, University of Alberta, Edmonton, Alberta, T6G 1H9, Canada

##### **Correspondence:** Edmond Lou (elou@ualberta.ca)

**Introduction**

Bracing is a proven non-surgical treatment for adolescent idiopathic scoliosis (AIS). Currently, in-brace Cobb angle (CA) is used to predict treatment outcomes. However, the association of the in-brace CA correction with in-brace apical axial vertebral rotation (AVR) or torsion corrections of the treated curves are unknown. Torsion is defined as the maximum AVR of curvature divided by half of the number of vertebrae within the curve. Understanding the associations may help orthotists to design a better brace.

**Objective**

To investigate if in-brace Cobb angle correction was associated with in-brace apical axial vertebral rotation (AVR) correction or in-brace torsion correction at the first follow-up clinic.

**Methods**

26 children with AIS (21 F, 5 M) were retrospectively extracted from our clinical records with ethics approval. The inclusion criteria were a) 10-16 years, b) Risser < 3, c) maximum CA 20^o^-40^o^, and d) prescription of full time TLSO with no prior bracing. Pre-brace and in-brace posteroanterior radiographs were used to extract CA, AVR based on Stokes method, and torsion of the treated curve. Pearson correlation was used to determine the association between in-brace corrections of (a) CA vs. AVR, (b) CA vs. torsion, and (c) AVR vs. torsion for both entire group and subgroups analyses. Six subgroups were categorized with mild and severe for CA and AVR, as well as small and large number of vertebrae within the curve (NVC), respectively. The thresholds in categorizing the 3 parameters were based on the corresponding average value of the pre-brace measurements from the entire group.

**Results**

For the entire group, the average in-brace CA, AVR and torsion correction were 46±23%, 14±91% and 7±87%, respectively. The Pearson correlation for (a), (b) and (c) analyses were R^2^ = 0.05, 0.03 and 0.96, respectively. No correlation was found between in-brace correction of CA with AVR or torsion. AVR and torsion correction had strong correlation. Regarding the subgroups analyses, for the mild CA (<29^o^, n = 14) and severe CA (≥29^o^, n = 12), the R^2^ values for (a), (b) and (c) were 0.07, 0.03, 0.94 and 0.03, 0.02, 0.97, respectively. The lateral curve severity has no effect on the correction of the in-brace CA with AVR or torsion. For mild AVR (<5, n=14) and severe AVR (≥5, n=12), the R^2^ values for (a), (b) and (c) were 0.30, 0.38, 0.96 and 0.39, 0.38, 0.94, respectively. However, for both (a) and (b), a positive and a negative association were found in the mild and severe AVR groups, respectively. For small NVC (≤6, n=10) and large NVC (>6, n=16), the R^2^ values for (a), (b) and (c) were 0.25, 0.20, 0.99, and 0.11, 0.08, 0.97, respectively. Length of curve has no effect on the correction of the in-brace CA with AVR or torsion.

**Conclusion**

Orthotists focus on in-brace CA correction while designing TLSO. There was no strong association between in-brace CA correction with in-brace AVR or torsion correction.

## P18 Patient-related outcomes in adolescents and young adults with idiopathic scoliosis undergoing spine deformity surgery – a cohort study based on a single centre spine outcomes registry

### Miranda L. van Hooff ^1,3^, Maarten Spruit ^2^, Philip P. Horsting ^2^, Marinus de Kleuver ^3^, Luuk W. L. de Klerk ^2^

#### ^1^ Sint Maartenskliniek, Department for Research, 6574 NA, Ubbergen, The Netherlands; ^2^ Sint Maartenskliniek, Department of Orthopaedics, 6574 NA, Ubbergen, The Netherlands; ^3^ Radboud university medical center, Department of Orthopaedics, 6525 GA, Nijmegen, The Netherlands

**Introduction**

Recently, international consensus was reached to systematically monitor patient-related outcomes (i.e. patient-reported outcome measures [PROMs] and clinician-based outcome measures) in deformity surgery for adolescents and young adults (AYA) with idiopathic scoliosis. Current studies are mainly based on radiographic outcomes and Cobb angles. Evaluation of outcome domains that matter to patients is recommended (e.g. functioning and health-related quality of life), but often remain underexposed.

**Objective**

To evaluate the two-year follow-up patient-related outcomes in AYA with idiopathic scoliosis undergoing spine deformity surgery.

**Method**

A single institution consecutive cohort study in The Netherlands. Since March 2014 all patients undergoing scoliosis surgery are systematically monitored over time and registered in an online web-system, which is connected to the patients’ electronic file. Routinely, relevant patient characteristics, radiological, and peri-operative parameters are reported. PROMs used: ODI (functioning), SRS22r and EQ5D (health-related quality of life), NRS (0-10; back and leg pain). Improvement over time (preoperative, 1-year and 2-year follow-up [fu]) in AYA, aged ≤25years, is evaluated with repeated measurement ANOVA (p<0.05). Clinical relevancy of PROMs is determined by published minimal clinical important changes (SRS22r) and by satisfactory symptom state comparable to healthy persons (ODI≤22). Clinician-based outcome measures were post-operative complications and revision surgery. The institution's review board approved the study and an opt out procedure was applied as ethical approval for this study was not required.

**Results and discussion**

91 patients with AYA idiopathic scoliosis were included for analysis (age 15.4years [SD2.6], range 12-25; females [n=72; 79.1%]), with primary Cobb of 56.1° (SD9.1). The majority had a Lenke type 1 (t1 n=42 [46.2%]; t2 n=16; t3 n=13; t4 n=5; t5 n=6; t6 n=6; not reported n=3). PROMs response rate at 2-year fu was 70.5% (91/129). Preoperatively, the non-responders did not differ from those who completed the PROMs at fu. Except for leg pain (F_[2,89]_=0.84; p=0.44), all PROMs showed a similar pattern; significant improvement up to 1-year fu and maintenance of results between 1 and 2-year fu. At 2-year fu 82 (90.1%) reached satisfied symptom state comparable to healthy persons (preoperatively n=73 [80.2%]) and 68 patients reached relevant improvement on SRS22r self-image (74.4%). 7 complications (7.7%) occurred in 7 patients of whom 1 patient (1.1%) required revision surgery for non-union.

**Conclusion and significance**

After spine deformity surgery AYA with idiopathic scoliosis improve significantly on all outcome domains and reach a relevant improvement in functioning and self-image. As scoliosis surgery is regarded as high complex care, continuous and standardized outcome monitoring is important to improve the quality of spine care, which is the basis for value-based spine care.

## P19 Radiographic comparison of patient-specific and manually contoured conventional rods in adolescent idiopathic scoliosis (AIS) surgery

### Pouya Alijanipour^1^; Michael J. Heffernan^1^; Nicholas K. Baldwin^1^; Afshin Aminian^2^; Andrew G. King^1^

#### ^1^Orthopedic Pediatric Spine Surgery, Children's Hospital of New Orleans, Louisiana State University Health Sciences Center, New Orleans, LA, USA; ^2^Children's Hospital Orange County, Orange, CA, USA

**Introduction**

In AIS surgery, it is unclear if pre-countoured patient-specific (PS) rods (based on digital planning using preoperative x-rays) are associated with an alignment advantage compared to conventional manually contoured (C) rods.

**Objective**

Evaluation whether PS rods are associated with more physiologic sagittal alignment and less rod contour change when compared to C rods.

**Methods**

Retrospective single-surgeon study of consecutive patients before and after use of PS rods. Operative AIS cases with either PS rods (n=21) or manually contoured C rods (n=20) and 1-year follow-up were included. Calibrated digital pre- and postoperative x-rays were assessed for spinal alignment and rod contour change (Δ: 1year-immediate postoperative x-rays for maximal rod deflection distance [MRDD] and angle of tangents to rod endpoints [AT]) using t-tests.

**Results and discussion**

There was no significant difference between PS and C groups in terms of age (mean: 15 vs 14.8 years), female gender (76 vs 85%), body mass index (21.7 vs 21.4 kg/m^2^), Cobb angle (57.1 vs 54.8°), pelvic incidence (49.5 vs 50.0°), sagittal parameters, surgery duration (201 vs 206 minutes), number of fused levels (10.2 vs 9.4), rod material (Titanium alloy rods: 81 vs 65%), rod diameter (6.0 vs 5.8 mm) and surgical complications (1 wound dehiscence vs none), respectively (all p values > 0.05). Postoperative x-rays showed no statistically significant difference in Cobb angles (13.7 vs 13.6°), thoracic kyphosis (27.1 vs 24.4°), lumbar lordosis (55.8 vs 57.6°) and rod contour change (ΔMRDD: 1.1 vs 1.4 mm; ΔAT: 1.8 vs 3.1°), respectively (all p values > 0.05). In patients with thoraco-lumbar (TL) fusion (n=13 in each group), postoperative TL angle (T10-L2) was significantly lordotic in C group (-7.3°) compared to PS group (-0.3°, p<0.001).

**Conclusion and significance**

In this study PS and C rods were generally associated with comparable sagittal spinal alignment and rod contour change 1 year after AIS surgery. The only exception was spinal TL junction, which was hyperlordotic in C group due to suboptimal positioning of thoracic and lumbar curves during manual rod contouring. This finding can be considerable in terms of its potential contribution to long-term risk of junctional disorders in AIS patients.

## P20 Quality of life and back pain in middle aged idiopathic scoliosis patients >20 years after brace or surgical treatment

### Johan L. Heemskerk^1^, Mark C. Altena^1^, Bernard E. E. M. J. Veraart^1^, Rene M. Castelein^2^, Diederik H. R. Kempen^1^

#### ^1^Department of Orthopedic surgery, OLVG, Amsterdam, The Netherlands; ^2^Department of orthopedic surgery, UMCU, Utrecht, The Netherlands

**Introduction**

Treatment of IS attempts to alter the natural history of this disease and prevent future problems in adulthood. However, there is limited information on the effects of treatments on QoL & back problems in middle aged IS patients.

**Objectives**

To evaluate QoL in middle aged IS patients and compare this with an age matched reference cohort without scoliosis.

**Methods**

IS patients, treated during childhood between 1978-1996 at the Amsterdam OLVG hospital, were selected from a historic database and contacted to participate in this study. Patients were treated with Boston brace or operated by Harrington spondylodesis at least 20 years ago. They were send a digital questionnaire focusing on back pain(Oswestry Disability Index) & QoL(SF-36). SF-36 was compared with a local age matched reference cohort(N=4172) in Amsterdam (mean age 43 yrs).

**Results**

Currently 183 patients completed the questionnaire of the 402 eligible patients. Patients(81% women) had an age of 43±3.6yrs with a follow up of 28±4yrs. 136 patients were brace treated(BT) and 47 were surgically treated(ST). BT patients had a Cobb of 32°±12 at end of treatment. Age at surgery was 16±3.1yrs with a Cobb of 57°±10 before surgery. At early adulthood, Cobb of the BT and ST group were 34°±14 and 35°±12, respectively. 70% of BT and 83% of the ST patients had back pain with a ODI of 9±10 and 19±19, respectively. Scores on the SF-36 domains were all lower in ST cohort compared to the BT cohort and were significant in 6 of the 8 subscales(P<0.03). Significant differences were larger than minimal clinically important differences. Compared to the reference cohort, BT patients had lower scores in 5 domains. Only the vitality subscale was significant(62±17 vs 69±19;P=0.005). The ST patients had significant lower scores(P<0.01) on all subscales compared to the reference cohort with exception of mental health. The differences in social functioning score(65±23 vs 85±22;P=0.002) and emotional role limitation score(58±32 vs 83±32.3;P=0.001) were the largest.

**Conclusions**

The study confirms that IS is a chronic disease with a serious impact on QoL in adulthood. Despite frequent back pain, pain intensity was not severe. Overall, Boston BT patients had better QoL scores compared to Harrington ST patients.

## P21 Clinical validation of a new 3D portable stereo-photogrammetric opto-electronic device for posture and 3D spine morphology measurement

### Moreno D’Amico^1,2^, Edyta Kinel^3^, Piero Roncoletta^1^

#### ^1^SMART Lab - Bioengineering & Biomedicine Company, Via C.D. Spiga 10, 65124 Pescara, Italy; ^2^ Department of Neuroscience, Imaging and Clinical Sciences, University G. D’Annunzio, Pescara, Italy; ^3^Department of Rheumatology and Rehabilitation, Clinic of Rehabilitation, University of Medical Sciences, Poznan, Poland

**Introduction**

3D Stereo-photogrammetric measurements allow evaluating posture and movement quantitatively. Unfortunately, up to now, this approach has been bounded to research or big rehabilitation centres with large facilities. Undoubtedly, ease of use, ultimate portability and reduced costs could be key points for the spread diffusion of this technology into the clinical rehabilitation field.

**Objectives**

This paper aimed to study the clinical applicability of a new developed self-calibrated, portable, opto-electronic stereo-photogrammetric device.

**Methods**

124 healthy subjects (57 females, 67 males), have been simultaneously analysed by using two different opto-electronic devices. Specifically, new portable and a standard lab version of the recently proposed integrated stereo-photogrammetric opto-electronic system named ***G.O.A.L.S***.^1^ Both the systems present a 3D reconstruction error below 1mm. The standard lab configuration system is equipped with 6 infrared cameras (0.3Mpix, 100 fps) allowing a large working volume. The portable is equipped with 3 infrared cameras (0.3Mpix, 120 fps) in a fixed configuration factory self-calibrated rig bar, but working on a smaller volume. The 3D raw data acquired by both the systems have been processed with the ***ASAP 3D Skeleton Model***^***1***^ biomechanical and clinical software. The limitation of the portable device is that only one side of the patient at a time can be analysed. However, a specially developed anatomical calibration procedure has been developed to provide, from the measures of the patient’s backside only, also an estimate of the head and pelvis 3D poses and dimensions. Five static acquisitions lasting 2 seconds each in the natural orthostatic condition have been simultaneously acquired with the two devices using a well-established 27 body landmarks clinical protocol for posture and 3D spine shape analysis. Averages and S.D. of the acquired series have been computed, and six main postural parameters have been extracted. Postural characteristics were compared for each variable of interest, applying a set of paired t-Tests with a significance level of α< 0.01.

**Results and discussion**

Both raw differences and t-Test on all the considered clinical parameters demonstrated the full agreement of the two devices measurements. Absolute raw differences values resulted clinically irrelevant and always below 1 S.D. unit.

The sources of the found differences are related to two main factors. The first is connected to the different calibration procedures applied to the two systems. The second could be referred to the not synched different acquisition rates, so, the natural postural oscillations of the subject are measured in slightly different moments of time.

**Conclusions and significance**

The two systems resulted in being equivalent in the analysis of posture and spine morphology, demonstrating the clinical usefulness of the portable system. This latter presents a series of advantages: self-calibrated, easy to transport and to install even in small rooms, relatively low cost. Thus, it can open the possibility to spread the quantitative 3D posture and spine shape assessment by the stereo-photogrammetric approach, even on small diagnostic and rehabilitation centres. Screenings in different populations are also possible.

^1^Bioengineering & Biomedicine Company srl - Pescara

## P22 Role of recreational sports activities on posture and spine shape characteristics in adolescents

### Moreno D’Amico^1,2^, Edyta Kinel^3^, Piero Roncoletta^1^

#### ^1^SMART Lab - Bioengineering & Biomedicine Company, Via C.D. Spiga 10, 65124 Pescara, Italy; ^2^ Department of Neuroscience, Imaging and Clinical Sciences, University G. D’Annunzio, Pescara, Italy; ^3^Department of Rheumatology and Rehabilitation, Clinic of Rehabilitation, University of Medical Sciences, Poznan, Poland

**Introduction**

The relationship between sports and spinal disease (spinal deformities and back pain), is a continuous debate. The International Society on Scoliosis Orthopedic and Rehabilitation Treatment (SOSORT) guidelines for the conservative treatment of scoliosis highlight the benefits of sports practice but clearly state that sports cannot be considered a treatment. In contrast, for many years some sport has been recommended and even prescribed by specialists as a specific treatment for spinal disorders, while others have been banned as dangerous.

**Objectives**

To verify if the practice of a recreational sports discipline at a rate of 2-3 hours/week induces significant postural changes in healthy adolescents.

**Methods**

To this aim, the postural characteristics of 4 groups of teenagers (11-17 years old) have been compared. A no sport control group (n = 32); 3 groups of adolescents practicing regular training at a rate of 2-3 hours/week, respectively soccer (n = 30), karate (n = 17) and swimming (n = 13). All such groups were homogeneous by age and BMI.

Subject’s full body posture including 3D spine shape reconstruction, using a well-established 27 retro-reflective markers suitably located on anatomical landmarks protocol, has been measured by using a non-ionising 3D optoelectronic stereo-photogrammetric system named *G.O.A.L.S*. The 3D raw data acquired have been processed with the ASAP 3D Skeleton Model biomechanical and clinical software. A total of 9 postural and spine shape parameters have been considered for the comparison.

A set of one-way unbalanced ANOVA p< 0.05 has been applied to check differences in each considered postural parameter among the groups. When the ANOVA gave a significant result, post-hoc comparisons have been performed.

**Results and discussion**

No differences were detected among the 4 groups for all postural parameters, except for the sagittal Global Offset (p = 0.000546). Post-hoc comparisons revealed a statistically significant difference between the control group and either the karate group (p=0.000451) or the swimming group (p=0.041) presenting these latter a more forward global offset (μcontrol = -5.8, μkarate = -32.3, μswimming = -24.7). Conversely, no differences were found between control and soccer group.

**Conclusions and significance**

The considered recreational sports activities at a rate of 2-3 hours/week do not produce significant benefits or drawbacks in postural and spine shape parameters. The results confirm the SOSORT guideline in that, sports practice produce benefits, but as we found these studied activities are not specific for posture, so, they should not be considered a treatment. At the same time, no one of the investigated sport should be seen as contraindicated or dangerous for the posture or spine disorders. The found results have to be confirmed with larger population samples and different sports. Future study will involve sports activity performed at a higher intensity for the agonistic level.

